# Neural Mechanisms Generating Orientation Selectivity in the Retina

**DOI:** 10.1016/j.cub.2016.05.035

**Published:** 2016-07-25

**Authors:** Paride Antinucci, Oniz Suleyman, Clinton Monfries, Robert Hindges

**Affiliations:** 1 MRC Centre for Developmental Neurobiology, King’s College London, Guy’s Campus, London SE1 1UL, UK

## Abstract

The orientation of visual stimuli is a salient feature of visual scenes. In vertebrates, the first neural processing steps generating orientation selectivity take place in the retina. Here, we dissect an orientation-selective circuit in the larval zebrafish retina and describe its underlying synaptic, cellular, and molecular mechanisms. We genetically identify a class of amacrine cells (ACs) with elongated dendritic arbors that show orientation tuning. Both selective optogenetic ablation of ACs marked by the cell-adhesion molecule Teneurin-3 (Tenm3) and pharmacological interference with their function demonstrate that these cells are critical components for orientation selectivity in retinal ganglion cells (RGCs) by being a source of tuned GABAergic inhibition. Moreover, our morphological analyses reveal that Tenm3^+^ ACs and orientation-selective RGCs co-stratify their dendrites in the inner plexiform layer, and that Tenm3^+^ ACs require Tenm3 to acquire their correct dendritic stratification. Finally, we show that orientation tuning is present also among bipolar cell presynaptic terminals. Our results define a neural circuit underlying orientation selectivity in the vertebrate retina and characterize cellular and molecular requirements for its assembly.

## Highlights

• We identify Tenm3^+^ ACs with elongated dendritic arbors showing orientation tuning
• Tenm3^+^ AC GABAergic inhibition is crucial for orientation-selective RGC tuning
• Orientation tuning is present also among some bipolar cell presynaptic terminals
• We propose a model of how orientation selectivity is generated in ganglion cells

## Graphical Abstract

**Figure:**
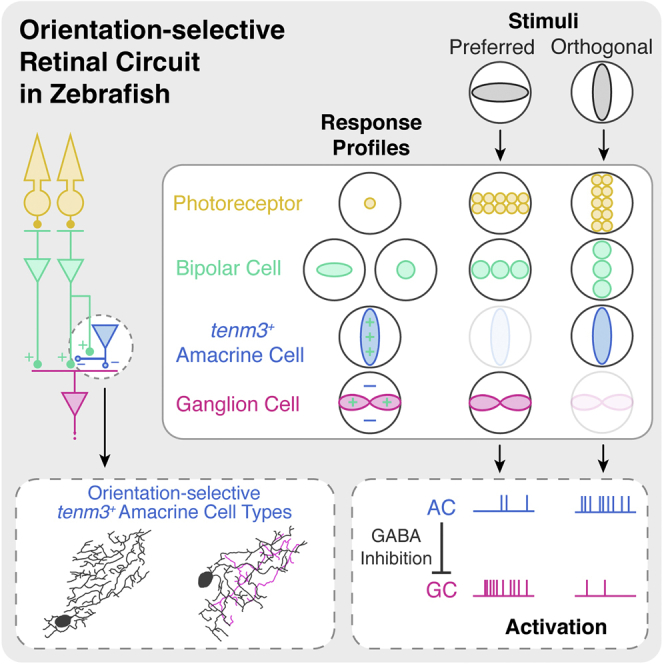

## Teaser

The neural mechanisms underlying orientation selectivity in the vertebrate retina are still poorly understood. Antinucci et al. identify a class of orientation-tuned amacrine cells characterized by elongated dendritic arbors that are essential for generating orientation selectivity in retinal ganglion cells.

## Introduction

The detection of oriented visual stimuli is a key neural computation performed by visual systems of many animals. Neurons performing this task are known as orientation selective (OS) since they respond preferentially to elongated stimuli oriented along a specific axis in the visual field but respond weakly to stimuli oriented orthogonally to their preferred axis. Orientation selectivity was first discovered in cat primary visual cortex by Hubel and Wiesel over 50 years ago [1]. Since then, numerous studies described OS neurons in visual systems of vertebrates and invertebrates, including primates [2], rodents [3], fish [4], and insects [5]. Work in several vertebrate species identified OS cells in regions upstream of primary visual cortex, like the lateral geniculate nucleus [6, 7, 8] and the retina [9, 10, 11, 12], suggesting that the first steps in the processing of oriented stimuli take place early along the vertebrate visual pathway. In the retina, orientation selectivity is present among retinal ganglion cells (RGCs) [10, 13], the sole retinal output neurons, and amacrine cells (ACs) [13, 14], a class of inhibitory neurons that modulate and shape RGC responses. However, it is presently unclear how orientation selectivity emerges in these cells and whether they form a distinct retinal circuit, partially due to the lack of specific molecular markers allowing targeted labeling and manipulations.

**Figure 1:**
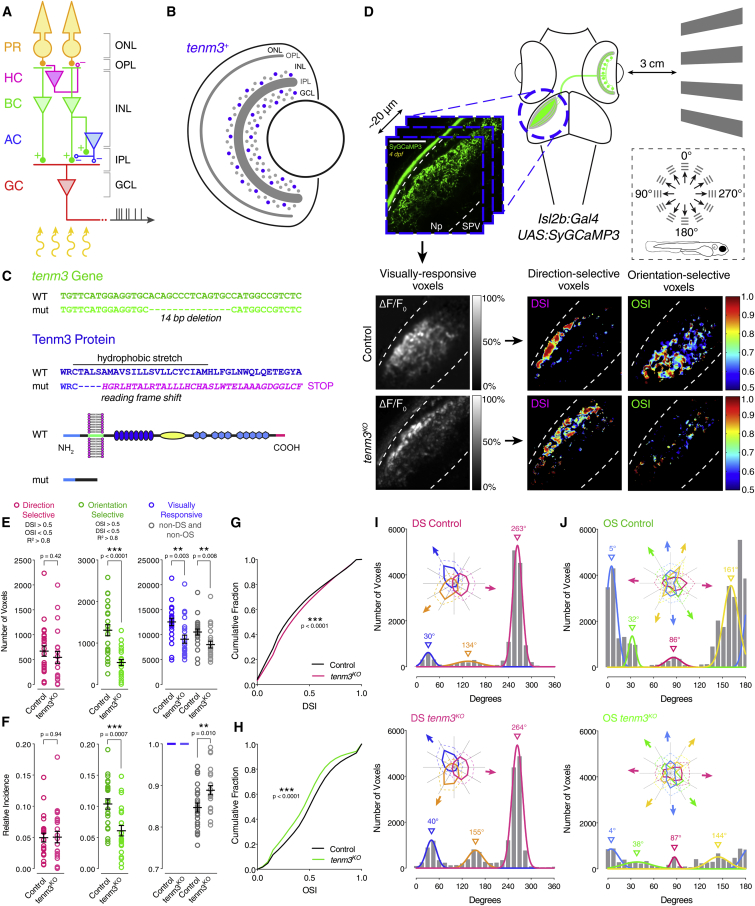
Tenm3 Is Required for RGC Orientation Selectivity (A) Basic neural circuit structure of the vertebrate retina. Cell classes are represented in colors, whereas layers are shown in black. Excitatory synapses are indicated by “+” (filled circles), inhibitory synapses are labeled with “–“ (empty circles). PR, photoreceptor; HC, horizontal cell; BC, bipolar cell; AC, amacrine cell; RGC, retinal ganglion cell; ONL, outer nuclear layer; INL, inner nuclear layer; GCL, ganglion cell layer; OPL, outer plexiform layer; IPL, inner plexiform layer. (B) Schematic showing *tenm3* mRNA expression in the retina of zebrafish larvae. Blue circles indicate *tenm3*^*+*^ ACs and RGCs. Adapted from Antinucci et al. [17]. (C) TALEN-mediated *tenm3* gene knockout (top) and consequent structural changes in the Tenm3 protein (bottom). (D) Functional calcium imaging of RGC axon terminals expressing SyGCaMP3 (green) in 4-dpf *Tg(isl2b:Gal4;UAS:SyGCaMP3)* larvae. Distance of right eye from projection screen is 3 cm. Recordings are performed from two to four Z-planes (approximately 20 μm total volume thickness) in the contralateral optic tectum. Dashed box shows the angles of moving bars relative to zebrafish larva orientation. Mean ΔF/F_0_ images of calcium recordings in control and *tenm3*^*KO*^ larvae followed by mapping of DS and OS voxels are displayed. Np, neuropil; SPV, stratum periventriculare; DSI, direction selectivity index; OSI, orientation selectivity index. Scale bar, 40 μm. (E and F) Average number (E) and relative frequency (F) of DS, OS, visually responsive, and non-DS/non-OS voxels per Z-plane in control (n = 23 larvae) and *tenm3*^*KO*^ (n = 22 larvae) 4-dpf larvae. Criteria used to identify DS and OS voxels are reported at the top. Error bars, ±SEM. ^∗∗^p < 0.01, ^∗∗∗^p < 0.001, unpaired two-tailed Student’s t test. (G and H) Cumulative distributions of DSI values (R^2^ > 0) across voxels with OSI < 0.5 (G) and OSI values (R^2^ > 0) across voxels with DSI < 0.5 (H) in control and *tenm3*^*KO*^ larvae. ^∗∗∗^p < 0.001, two-sample Kolmogorov-Smirnov test. (I and J) Cumulative histograms summarizing the incidence of preferred angles for identified DS (I) and OS voxels (J) in control (n = 23; top) and *tenm3*^*KO*^ (n = 22; bottom) 4-dpf larvae. Overlaid curves are the fitted Gaussian distributions for each DS or OS subtype. Polar plots illustrate the mean (+1 SD) normalized response profiles for each DS or OS subtype. See also Figure S1, Table S1, and Movie S1.

The vertebrate retina consists of more than 70 neuron types [15]. Its primary function is to detect light stimuli, convert them into electrochemical signals, and, subsequently, send the processed information to higher visual nuclei through parallel feature-specific neural pathways. Most of the information processing takes place in a layered neuropil structure called the inner plexiform layer (IPL) [16]. Essential neural substrates underlying the computations performed in the IPL are the specific and stereotypic synaptic connections between three classes of neurons, namely, bipolar cells (BCs), ACs, and RGCs (Figure 1A). Recent developmental studies have shown that cell-adhesion molecules selectively expressed in specific retinal cell types mediate the matching between defined pre- and postsynaptic partners to establish this complex wiring pattern [18, 19, 20]. While several cell types and molecules crucial for establishing direction selectivity in the retina have been identified [20, 21], the equivalents for orientation selectivity are largely unknown to date.

Teneurins are a family of transmembrane cell-adhesion proteins that play a synaptic matching role in the *Drosophila* olfactory system [22] and neuromuscular junction [23]. In vertebrates, teneurins are highly expressed in several interconnected regions of the brain, including the visual system [24, 25]. In vitro and in vivo data suggest that *trans*-synaptic interactions are possible both homophilically through their five NHL domains [22, 26], and heterophilically with the cell-adhesion G-protein-coupled receptors latrophilins [27, 28]. We previously showed that *teneurin-3* (*tenm3*) is expressed in zebrafish ACs and RGCs during a period of intense synaptic formation (Figure 1B) and that, when *tenm3* is knocked down, RGC dendrites fail to correctly stratify in the IPL [17]. We also reported a functional link between *tenm3* and RGC orientation selectivity.

Here, using *tenm3* as a marker, we identify crucial cellular players and mechanisms generating orientation selectivity in the larval zebrafish retina. First, we reveal that *tenm3*-expressing (*tenm3*^*+*^) ACs co-stratify their neurites with orientation-selective RGC (OSGC) dendrites and that, upon *tenm3* knockout, they fail to correctly stratify their neurites in the IPL. Second, we show evidence suggesting that *tenm3*^*+*^ ACs generate OSGC tuning by providing γ-aminobutyric acid (GABA) feedforward inhibitory input. Third, we identify and characterize orientation-tuned *tenm3*^*+*^ AC types with elongated dendritic arbors. Fourth, we find that a fraction of BC presynaptic terminals show orientation tuning. Finally, we present a circuit model describing how OSGCs acquire their orientation selectivity by integrating tuned *tenm3*^*+*^ ACs and BC inputs.

## Results

### Generation of a Tenm3^KO^ Tool to Study Retinal Orientation Selectivity

Previous work using transient gene knockdown methods suggested that Tenm3 is involved in the development of orientation selectivity in zebrafish RGCs [17]. To confirm this role and, subsequently, use Tenm3 as a marker to identify cells generating RGC orientation selectivity, we generated a zebrafish *tenm3* knockout mutant (*tenm3*^*KO*^) using Transcription Activator-Like Effector Nuclease (TALEN)-based genome editing (see Supplemental Experimental Procedures). In *tenm3*^*KO*^ mutants, a 14-bp deletion in the exon encoding the transmembrane domain of Tenm3 leads to a reading frameshift and, subsequently, to a premature stop codon causing the loss of the entire extracellular domain (Figures 1C and S1A–S1F). We then examined the retinal functional output of *tenm3*^*KO*^ mutants as previously described [4, 17]. Briefly, drifting bars moving in 12 different directions were presented to awake immobilized zebrafish larvae through a projection screen (Figure 1D). Using the RGC-specific transgenic line *Tg(isl2b:Gal4;UAS:SyGCaMP3)*, population visual responses were simultaneously recorded through calcium imaging of RGC axon terminals in the contralateral optic tectum (Movie S1). Voxel-wise analysis was then used to isolate visually responsive voxels and identify direction-selective (DS) and OS responses (Figures 1D and S1G) [29]. We found that 4 days post-fertilization (dpf) *tenm3*^*KO*^ mutants have a large decrease in both the number of OS voxels (Figure 1E) and the proportion of OSGC output relative to the whole population of responsive voxels (Figure 1F). As a consequence, the relative proportion of “non-tuned” (non-DS and non-OS) RGC output was increased in *tenm3*^*KO*^ mutants (Figure 1F). This impairment in the OSGC population is consistent with the lower degree of orientation selectivity, quantified by the orientation selectivity index (OSI), across visually responsive voxels in *tenm3*^*KO*^ mutants (Figure 1H). By contrast, the direction-selective RGC (DSGC) population of responses did not show any impairment in *tenm3*^*KO*^ mutants (Figures 1E–1G), suggesting that Tenm3 is not involved in the assembly of DS circuits. Equivalent results were obtained in 7-dpf *tenm3*^*KO*^ mutants (Figures S1H–S1K), indicating that the development of RGC orientation selectivity is not simply delayed. A modest but significant decrease in the number of visually responsive voxels was observed in *tenm3*^*KO*^ mutants at 4 dpf (Figure 1E), but not at 7 dpf (Figure S1H).

We next explored to what extent the subtypes of DSGCs and OSGCs previously described in zebrafish [29] were affected by Tenm3 loss of function. Different subpopulations of DSGC and OSGC responses were identified by fitting Gaussian distributions to the grouped population data of preferred angles [4, 29]. As expected, population sizes and relative proportions of the three subtypes of DSGCs were not altered in *tenm3*^*KO*^ mutants (Figures 1I and S1L), reinforcing the view that RGC direction selectivity develops through Tenm3-independent mechanisms. Interestingly, the decrease in OS responses in *tenm3*^*KO*^ mutants was not equally represented among the four OSGC subtypes, with the small OSGC subpopulation tuned to vertical bars moving along the horizontal axis being the least affected (magenta, Figures 1J and S1M). Overall, these results confirm and further elucidate the crucial role played by Tenm3 in generating RGC orientation selectivity during development [17]. Additionally, they provide a genetic access point to reveal the individual circuit components and mechanisms underlying retinal orientation selectivity.

### Neurite Stratification Pattern of Tenm3^+^ ACs and OSGCs

**Figure 2:**
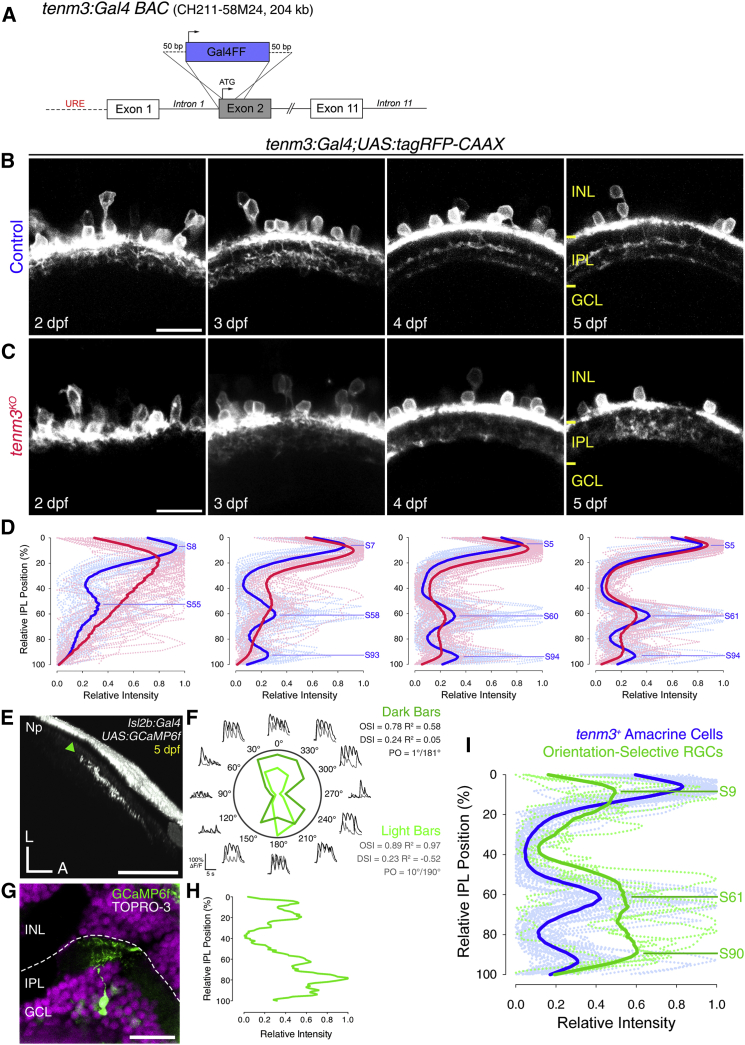
IPL Stratification Pattern of Tenm3^+^ ACs and OSGCs (A) Schematic of the bacterial artificial chromosome (BAC) DNA construct used to transgenically express Gal4FF in *tenm3*^*+*^ cells. URE, upstream regulatory elements. (B and C) Inner plexiform layer (IPL) stratification pattern of *tenm3*^*+*^ AC neurites in control (B) and *tenm3*^*KO*^ (C) *Tg(tenm3:Gal4;UAS:tagRFP-CAAX)* larvae from 2 to 5 dpf. INL, inner nuclear layer; GCL, ganglion cell layer. Scale bars, 20 μm. (D) IPL fluorescence intensity profiles of *tenm3*^*+*^ AC neurites in control (blue; n = 13 larvae) and *tenm3*^*KO*^ larvae (red; n = 13 larvae) from 2 to 5 dpf. Thin traces represent individual IPL profiles, whereas thick traces indicate average IPL profiles. 0% corresponds to the INL/IPL boundary, whereas 100% corresponds to the IPL/GCL boundary. Fluorescence peaks indicating IPL strata in control larvae are labeled with the letter “S” followed by their relative IPL position. (E and F) Visual responses to moving bars (F) recorded through calcium imaging of an individual orientation-selective RGC (OSGC) axon terminal expressing GCaMP6f (E, green arrowhead) in the optic tectum of a 5-dpf *UAS:GCaMP6f*-injected *Tg(isl2b:Gal4)* larva. Polar plots show the integral responses to moving dark and light bars (F; dark and light green, respectively). Black and gray traces represent the ΔF/F_0_ calcium responses to moving dark and light bars, respectively. Np, neuropil; L, lateral; A, anterior; PO, preferred orientation. Scale bar, 40 μm. (G and H) Immunostaining for GCaMP6f (green) showing the dendritic morphology (G) of the functionally identified OSGC in (E) and the corresponding normalized IPL fluorescence intensity profile (H). Cell bodies are labeled with the nuclear stain TO-PRO-3 (magenta). Scale bar, 20 μm. (I) IPL fluorescence intensity profiles of OSGCs (green; n = 5 cells) and *tenm3*^*+*^ AC neurites (blue; n = 13 larvae) at 5 dpf. 12.8% of functionally imaged RGCs were OS (five out of 39 cells in 39 larvae). Thin traces represent individual IPL profiles, whereas thick traces indicate average IPL profiles. Fluorescence peaks indicating IPL strata formed by OSGC dendrites are labeled with the letter “S” followed by their relative IPL position. See also Figures S2 and S3 and Movie S2.

Since *tenm3* is expressed not only in RGCs, but also in ACs [17], we asked whether the functional phenotype observed in *tenm3*^*KO*^ OSGCs results from structural defects in the presynaptic AC population. We thus generated a zebrafish bacterial artificial chromosome (BAC) transgenic line, *Tg(tenm3:Gal4)*, where Gal4FF is under transcriptional control of regulatory elements upstream and downstream of the *tenm3* start codon (Figure 2A) (see Supplemental Experimental Procedures). In this BAC line, Gal4 is expressed in brain regions where *tenm3* is endogenously expressed, including the retina and optic tectum (Figure S2) [17]. In the retina, the *Tg(tenm3:Gal4;UAS:tagRFP-CAAX)* line labels a subset of ACs (hereafter referred to as *tenm3*^*+*^ ACs) but fails to drive expression in RGCs (Figures 2B and S2A–S2C; Movie S2), possibly due to a lack of RGC-specific regulatory elements in the BAC construct used for transgenesis. We therefore used this line to selectively visualize the morphological development of *tenm3*^*+*^ ACs from 2 to 5 dpf. During this period, *tenm3* is highly expressed in the retina [17], and, at 3 dpf, RGCs start to show orientation selectivity [29]. Interestingly, *tenm3*^*+*^ ACs stratify their neurites in three precise IPL strata located at 5%, 61%, and 94% depth (0% corresponds to the inner nuclear layer (INL)/IPL border, 100% to the IPL/GCL border), which were named S5, S61, and S94, respectively (Figures 2B and 2D). This tri-laminar IPL stratification pattern is visible at 3 dpf and gradually refines over the following 2 developmental days. In *tenm3*^*KO*^ mutants, by contrast, *tenm3*^*+*^ AC neurites do not stratify correctly in the IPL (Figure 2C). This is particularly striking at 3 dpf when they fail to target the two innermost IPL strata and instead stratify diffusely across the IPL (Figure 2D).

An indication of potential synaptic connections between *tenm3*^*+*^ ACs and OSGCs would be their dendritic co-stratification in the IPL. Currently, no transgenic line exists to selectively label OSGCs and directly detect dendritic co-stratification with *tenm3*^*+*^ ACs. Therefore, we sparsely expressed GCaMP6f [30] in individual RGCs and, after functionally identifying OSGCs, we performed post hoc immunostaining to analyze their IPL stratification pattern (Figures 2E–2G). Then, we averaged fluorescence intensity profiles of dendritic stratification from multiple OSGCs (Figures 2H and S3) and overlaid the resulting mean profile (green, Figure 2I) to the IPL stratification profile of *tenm3*^*+*^ ACs (blue). We found that, as a population, OSGCs stratify their dendrites in three strata located at 9%, 61%, and 90% IPL depth and indeed show a high degree of overlap with *tenm3*^*+*^ AC neurites (Figure 2I). These results, together with the functional impairment in RGC orientation selectivity (Figure 1) and previously reported defects in RGC dendritic IPL stratification following Tenm3 loss of function [17] strongly suggest that *tenm3*^*+*^ ACs and OSGCs are part of the same circuit and that Tenm3 is involved in the morphological and functional development of these two neural populations.

### Tenm3^+^ ACs Generate OSGC Tuning through GABAergic Inhibition

**Figure 3:**
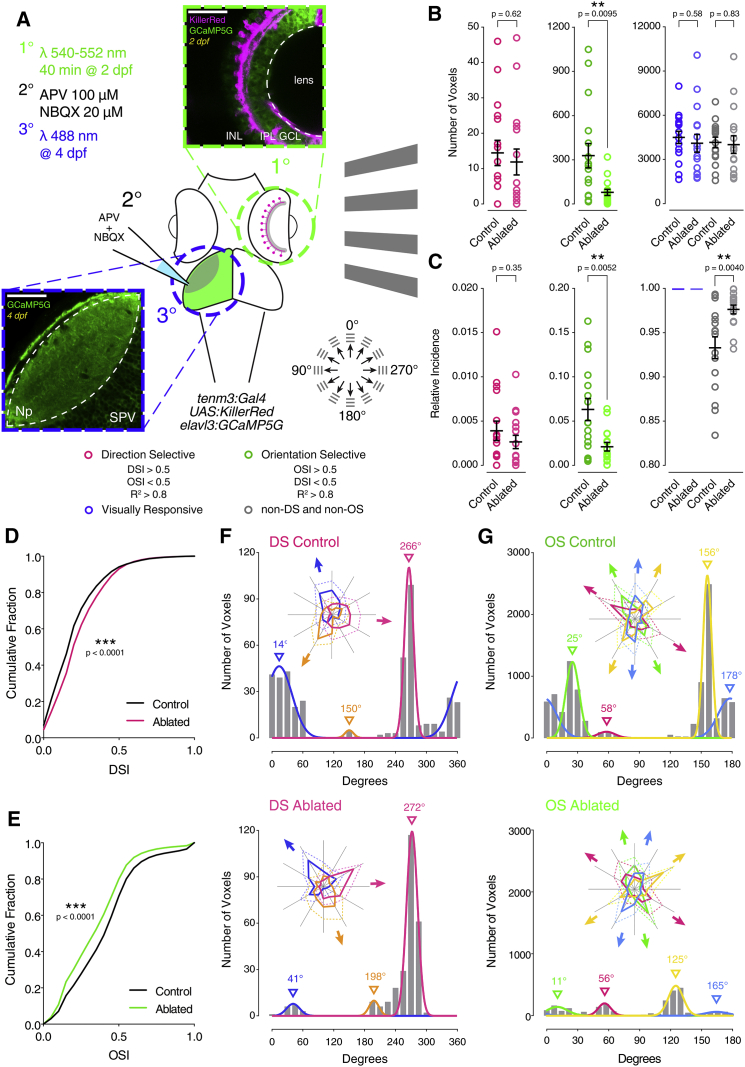
Tenm3^+^ ACs Generate Orientation Tuning in RGCs (A) Summary of the experimental procedures used to record visual responses from larvae where *tenm3*^*+*^ ACs were optogenetically ablated. At 2 dpf, the eyes of *Tg(tenm3:Gal4;UAS:KillerRed;elavl3:GCaMP5G)* larvae, where KillerRed is selectively expressed in *tenm3*^*+*^ ACs only (magenta), are illuminated with green light (540–552 nm) for 40 min. Then, at 4 dpf, visual responses to moving bars are recorded through calcium imaging of RGC axon terminals (expressing GCaMP5G; green) in the optic tectum contralateral to the illuminated eye. Local application of the glutamate receptor antagonists APV and NBQX (100 and 20 μM, respectively) is used to isolate RGC axonal calcium responses from tectal cell dendritic responses. INL, inner nuclear layer; GCL, ganglion cell layer; IPL, inner plexiform layer; Np, neuropil; SPV, stratum periventriculare. Scale bars, 40 μm. (B and C) Average number (B) and relative frequency (C) of DS, OS, visually responsive, and non-DS/non-OS voxels per Z-plane in control (n = 16 larvae) and *tenm3*^*+*^ AC ablated (n = 16 larvae) 4-dpf larvae. Error bars, ±SEM. ^∗∗^p < 0.01, unpaired two-tailed Student’s t test. (D and E) Cumulative distributions of DSI values (R^2^ > 0) across voxels with OSI < 0.5 (D) and OSI values (R^2^ > 0) across voxels with DSI < 0.5 (E) in control and *tenm3*^*+*^ AC ablated larvae. ^∗∗∗^p < 0.001, two-sample Kolmogorov-Smirnov test. (F and G) Cumulative histograms summarizing the incidence of preferred angles for identified DS (F) and OS voxels (G) in control (n = 16; top) and *tenm3*^*+*^ AC ablated (n = 16; bottom) 4-dpf larvae. Overlaid curves are the fitted Gaussian distributions for each DS or OS subtype. Polar plots illustrate the mean (+1 SD) normalized response profiles for each DS or OS subtype. See also Figure S4, Table S1, and Movie S3.

To investigate whether *tenm3*^*+*^ ACs play a role in the emergence of RGC orientation selectivity, we took advantage of the *Tg(tenm3:Gal4)* line to selectively ablate these cells and assess the functional consequences in RGCs. In the *Tg(tenm3:Gal4;UAS:KillerRed;elavl3:GCaMP5G)* line, the genetically encoded photosensitizer KillerRed [31] is expressed in *tenm3*^*+*^ ACs, whereas GCaMP5G is expressed pan-neuronally (Figure 3A) [32]. At 2 dpf, we optogenetically ablated *tenm3*^*+*^ ACs by illuminating the retina with intense green light (540–552 nm) for 40 min (Figure S4; see Supplemental Experimental Procedures). Subsequently, at 4 dpf, we recorded RGC visual responses to moving bars as described above (Movie S3). To isolate RGC axonal calcium responses from tectal cell dendritic responses, we locally applied the glutamate receptor antagonists APV and NBQX (100 and 20 μM, respectively) in the tectum [33]. Unlike KillerRed-positive larvae, control larvae subjected to the same procedures did not exhibit retinal cell death (Figure S4). Animals subjected to *tenm3*^*+*^ AC ablation showed a dramatic impairment in RGC orientation selectivity but no detrimental change in DSGC responses (Figures 3B–3E). The magnitude of the decrease in number of OS voxels, relative proportion of OSGC output and overall degree of RGC orientation selectivity was analogous to what we observed in *tenm3*^*KO*^ mutants. Moreover, the OSGC subpopulation tuned to vertical stimuli was the least affected by *tenm3*^*+*^ AC ablation (magenta, Figure 3G), matching our Tenm3 KO results. Compared to data acquired using the *Tg(isl2b:Gal4;UAS:SyGCaMP3)* line (Figures 1I and 1J), we observed differences in the relative proportions of DSGC and OSGC subtypes as well as in their preferred directions or orientations both in control and *tenm3*^*+*^ AC ablated groups (Figures 3F and 3G), likely resulting from the use of a different transgenic line or the pharmacological treatment used to isolate RGC responses.

**Figure 4:**
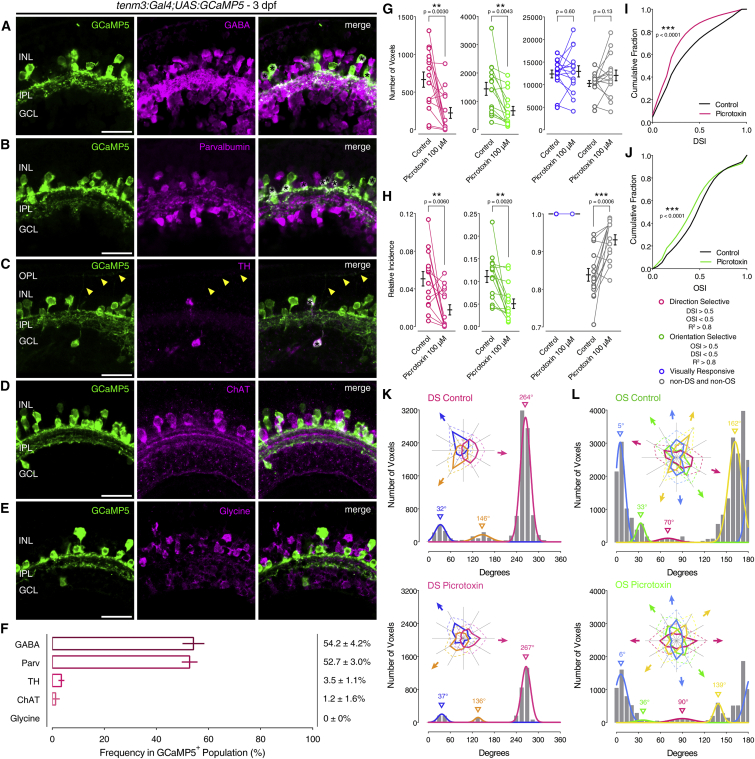
Role of Tenm3^+^ AC GABAergic Inhibition in RGC Orientation Selectivity (A–E) Immunostaining showing the expression of γ-aminobutyric acid (GABA; A), parvalbumin (B), tyrosine hydroxylase (TH; C), choline acetyltransferase (ChAT; D), and glycine (E) (all in magenta) in 3-dpf *Tg(tenm3:Gal4;UAS:GCaMP5)* larvae, where *tenm3*^*+*^ ACs are labeled with GCaMP5 (green). Yellow arrowheads indicate neurites of TH^+^ interplexiform ACs (C). INL, inner nuclear layer; GCL, ganglion cell layer; OPL, outer plexiform layer; IPL, inner plexiform layer. Scale bars, 20 μm. (F) Percentage of GCaMP5^+^ cells co-localizing with antigen^+^ cells (mean ± SD). GABA, n = 13 retinae; Parvalbumin, n = 10 retinae; TH, n = 9 retinae; ChAT, n = 10 retinae; glycine, n = 5 retinae. (G and H) Average number (G) and relative frequency (H) of DS, OS, visually responsive, and non-DS/non-OS voxels per Z-plane in 4-dpf *Tg(isl2b:Gal4;UAS:SyGCaMP3)* larvae (n = 15 larvae) before (control) and after (picrotoxin) the application of picrotoxin (100 μM). Error bars, ±SEM. ^∗∗^p < 0.01, ^∗∗∗^p < 0.001, paired two-tailed Student’s t test. (I and J) Cumulative distributions of DSI values (R^2^ > 0) across voxels with OSI < 0.5 (I) and OSI values (R^2^ > 0) across voxels with DSI < 0.5 (J) before (control) and after (picrotoxin) the application of picrotoxin (100 μM). ^∗∗∗^p < 0.001, two-sample Kolmogorov-Smirnov test. (K and L) Cumulative histograms summarizing the incidence of preferred angles for identified DS (K) and OS voxels (L) in 4-dpf larvae (n = 15 larvae) before (control) and after (picrotoxin) the application of picrotoxin (100 μM). Overlaid curves are the fitted Gaussian distributions for each DS or OS subtype. Polar plots illustrate the mean (+1 SD) normalized response profiles for each DS or OS subtype. See also Figure S5 and Table S1.

The results obtained by ablating *tenm3*^*+*^ ACs strongly support the idea that the output of *tenm3*^*+*^ ACs is crucial in generating RGC orientation selectivity. We therefore aimed to reveal the role played by *tenm3*^*+*^ AC neurotransmission in performing this neural computation. Immunohistochemical analyses showed that most *tenm3*^*+*^ ACs are GABAergic and express the calcium-binding protein Parvalbumin (Figures 4A, 4B, and 4F). *Tenm3*^*+*^ ACs also comprise dopaminergic ACs, which constitute a very small fraction of the whole AC population in zebrafish (Figure 4C) [34]. Negligible or no co-localization was observed between *tenm3*^*+*^ ACs and cholinergic or glycinergic ACs, respectively (Figures 4D–4F). Cholinergic ACs correspond to starburst ACs (SACs), which are key cellular players in generating RGC direction selectivity [35], consistent with the observation that neither Tenm3 KO nor *tenm3*^*+*^ AC ablation affected DSGC tuning. We next tested the role of GABA-mediated inhibition in producing OSGC tuning by blocking GABA_A_ receptors through picrotoxin (100 μM). RGC visual responses were recorded from the same *Tg(isl2b:Gal4;UAS:SyGCaMP3)* larvae before and after drug application. Notably, OSGCs were severely affected by GABA inhibition block, with a decrease in both OS responses and overall degree of RGC orientation selectivity comparable to the effects seen in *tenm3*^*KO*^ mutants and after *tenm3*^*+*^ AC ablation (Figures 4G, 4H, and 4J). Similarly to the knockout and ablation experiments, the small OSGC subpopulation tuned to vertical bars was the least affected by the pharmacological block (magenta, Figure 4L). As expected, RGC direction selectivity was also negatively impacted (Figures 4G–4I and 4K), since directionally tuned GABAergic inhibitory input from SACs plays an essential role in most DSGCs [35]. Compared to the impairments in RGC direction and orientation selectivity caused by blocking GABA_A_ receptors, the effects produced by blocking glycine receptors using strychnine (70 μM) were minimal (Figures S5A–S5F). Taken together, these results demonstrate that OSGCs require GABAergic inhibitory input, likely from *tenm3*^*+*^ ACs, to acquire their orientation tuning.

### Single-Cell Morphologies of Tenm3^+^ AC Types

**Figure 5:**
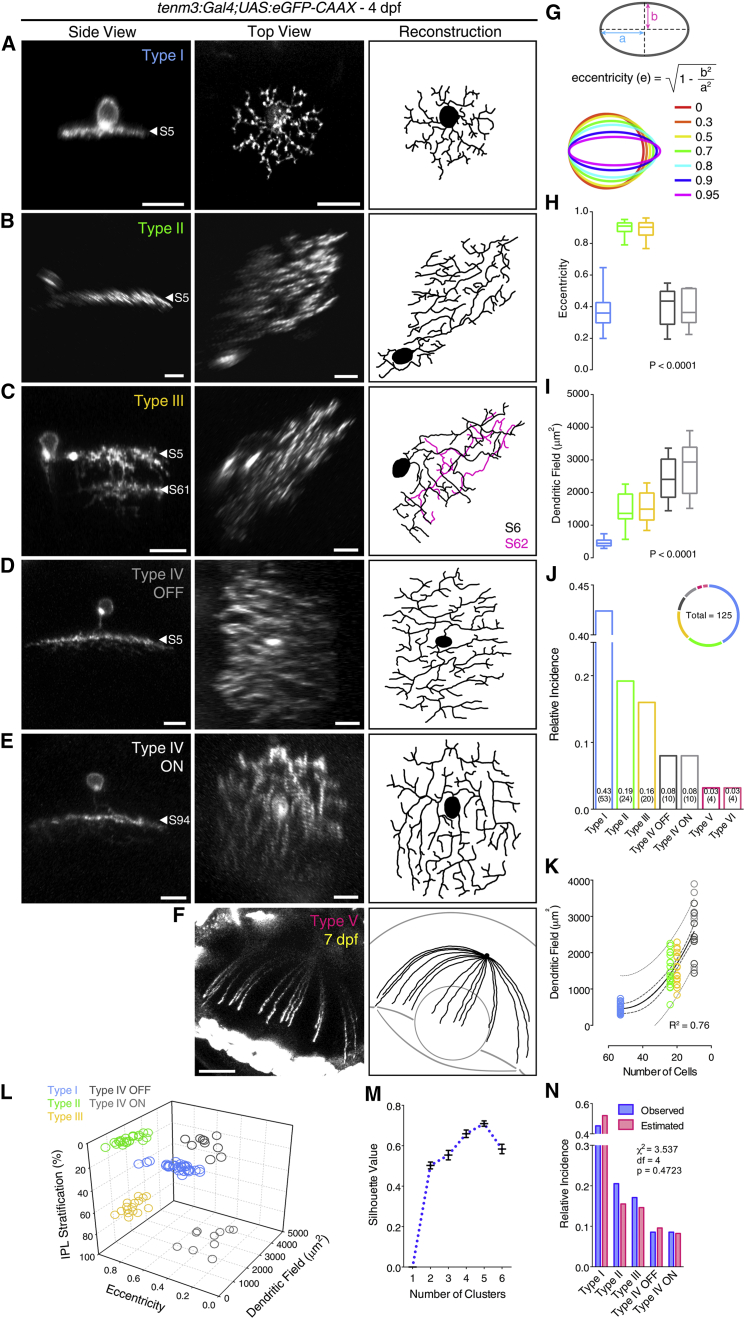
Morphological Diversity of Individual Tenm3^+^ AC Types (A–F) Morphologies of single *tenm3*^*+*^ ACs expressing eGFP-CAAX in 4-dpf *UAS:eGFP-CAAX*-injected *Tg(tenm3:Gal4)* larvae. The side views (left), top views (middle), and top view 3D reconstructions (right) are shown. IPL strata location of *tenm3*^*+*^ AC neurites is indicated by the letter “S” followed by the relative IPL position. The 3D reconstructed neurites of the bi-stratified type III *tenm3*^*+*^ AC are color-coded according to the stratum they are located. Note that the type V *tenm3*^*+*^ AC shown in (F) is from a 7-dpf larva. Scale bars, 10 μm in (A)–(E) and 40 μm in (F). (G) Diagram illustrating the quantification of dendritic field elongation by calculating the eccentricity of dendritic arbor profiles following ellipse fitting. “a” is the length of the semi-major axis, and “b” is the length of the semi-minor axis. (H–J) Dendritic field elongation (i.e., eccentricity; H), dendritic field area (I), and relative frequency (J) of identified *tenm3*^*+*^ AC types (n = 125 cells from 65 larvae). The number of observed cells for each *tenm3*^*+*^ AC type is reported in brackets (J). Boxplots indicate interquartile ranges (boxes), medians (lines in boxes), and ranges (min-max, whiskers). p values are the results of one-way ANOVA. (K) Relationship between dendritic field area of type I–IV *tenm3*^*+*^ ACs and their observed frequency (in number of cells). The continuous curve shows the nonlinear regression of the data with a second order polynomial function indicating a quadratic relationship between the two variables. Thick and thin dashed curves report the 95% confidence and prediction bands of the nonlinear fit, respectively. Goodness of fit value (R^2^) is reported at the bottom-right corner. (L and M) k-means clustering of type I–IV *tenm3*^*+*^ ACs based on their IPL stratification, dendritic field area, and dendritic field elongation. Individual cell data points are color coded according to which *tenm3*^*+*^ AC type they have been classified (L). Analysis of mean silhouette values for increasing number of clusters indicates that five clusters best describe the dataset (M). Importantly, the five cell clusters obtained by k-means are consistent with the classification of the most frequent *tenm3*^*+*^ ACs into five different types. Error bars, ±SEM. (N) Observed (blue) and estimated (red; assuming a retinal coverage factor of 1) relative frequencies of type I–IV *tenm3*^*+*^ ACs. Results of the two-tailed chi-square test are reported. See also Table S1.

To explore a possible link between the morphology of *tenm3*^*+*^ ACs and the function they play in the OS circuit, we sparsely labeled *tenm3*^*+*^ ACs by injecting *UAS:eGFP-CAAX* DNA constructs into 1-4 cell-stage *Tg(tenm3:Gal4)* embryos. We identified seven types of *tenm3*^*+*^ ACs characterized by distinct morphological properties (Figures 5A–5F). These types differ in terms of their observed frequency, IPL dendritic stratification, dendritic field area (Figures 5I and 5J), and, interestingly, dendritic field elongation, quantified by calculating the eccentricity of their dendritic fields (Figures 5G and 5H). The most frequent *tenm3*^*+*^ AC type (type I, 43% of *tenm3*^*+*^ ACs) is a narrow-field AC with a dendritic arbor mono-stratified in S5 (Figure 5A). Type II and III *tenm3*^*+*^ ACs (19% and 16% of *tenm3*^*+*^ ACs, respectively) are medium-field ACs characterized by highly elongated dendritic fields (Figures 5B, 5C, and 5H). Their dendritic arbors stratify differently in the IPL with type II *tenm3*^*+*^ ACs having mono-stratified neurites in S5, and type III *tenm3*^*+*^ ACs showing a bi-stratified dendritic arbor in S5 and S61. Type IV-ON and IV-OFF *tenm3*^*+*^ ACs (each 8% of *tenm3*^*+*^ ACs) are mono-stratified medium-field ACs characterized by circular dendritic fields of similar area but different IPL stratification pattern, with the ON type stratifying in the innermost stratum (S94) and the OFF type in the outermost stratum (S5; Figures 5D and 5E). Finally, type V and VI *tenm3*^*+*^ ACs are the least frequent *tenm3*^*+*^ AC types (each 3% of *tenm3*^*+*^ ACs) and possess wide-field dendritic arbors. Type V has extensive, radially arranged neurites covering most of the retina (Figure 5F). Type VI corresponds to the dopaminergic interplexiform AC previously described in goldfish (Figure 4C) [36]. Importantly, k-means clustering based on IPL stratification, dendritic field area, and dendritic field elongation support the notion that the different *tenm3*^*+*^ ACs identified here are indeed defined AC types (Figures 5L and 5M; type V and VI ACs were not included in the clustering). Moreover, several lines of evidence suggest that type I–IV *tenm3*^*+*^ ACs are arranged in mosaics tiling the retina with a coverage factor close to 1: (1) their frequency scales quadratically as a function of their dendritic field area (Figure 5K); (2) their observed frequency does not differ significantly from the frequency estimated assuming a retinal coverage factor of 1 (Figure 5N). Interestingly, the high dendritic field elongation of type II and III *tenm3*^*+*^ ACs (Figures 5B, 5C, and 5H) is a feature previously described also in rabbit orientation-sensitive ACs [13, 14]. This led us to hypothesize that type II and III *tenm3*^*+*^ ACs could produce OS responses when stimulating the retina with elongated stimuli oriented along particular axes in the visual field and, consequently, constitute cellular elements underlying the emergence of retinal orientation selectivity.

### Tenm3^+^ ACs Show Orientation Tuning

**Figure 6:**
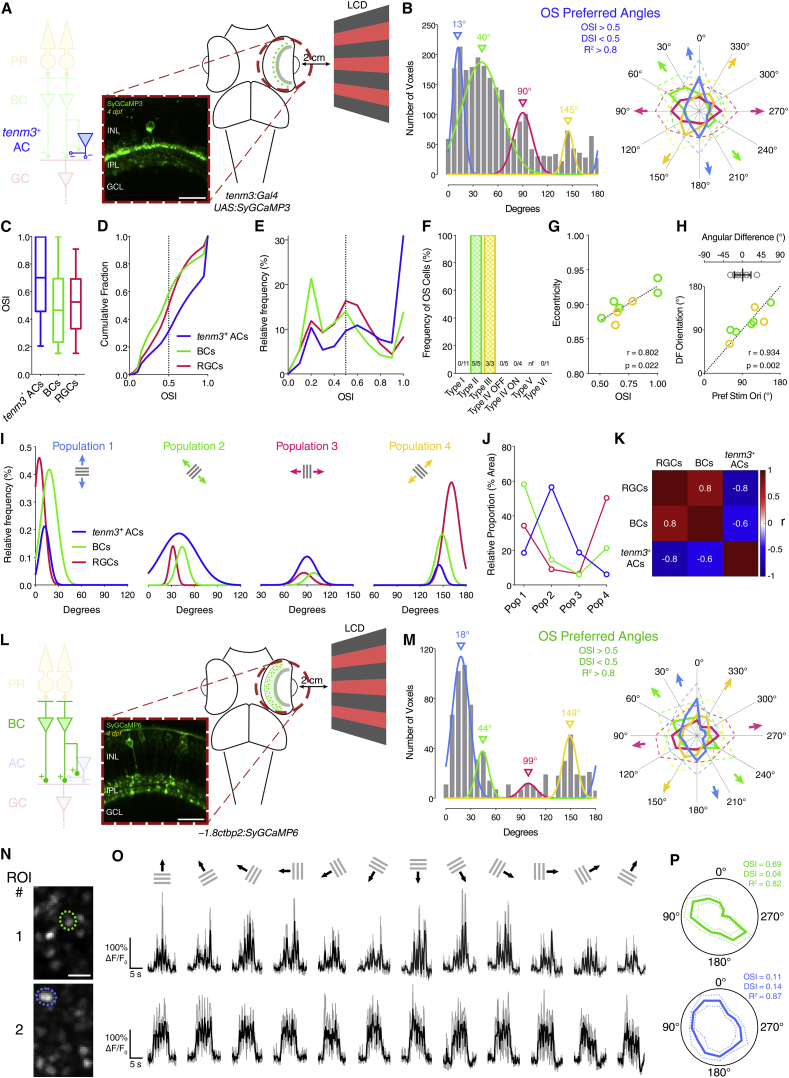
Orientation Selectivity in Tenm3^+^ ACs and BC Terminals (A) Two-photon functional calcium imaging of *tenm3*^*+*^ AC synaptic terminals expressing SyGCaMP3 (green) in 4-dpf *Tg(tenm3:Gal4;UAS:SyGCaMP3)* larvae. Distance of the eye from LCD screen is 2 cm. Recordings are performed from two to four Z-planes (approximately 20 μm total volume thickness). INL, inner nuclear layer; GCL, ganglion cell layer; IPL, inner plexiform layer. Scale bar, 20 μm. (B) Cumulative histogram summarizing the incidence of preferred angles for identified *tenm3*^*+*^ AC OS voxels in 4-dpf larvae (n = 20 larvae). Overlaid curves are the fitted Gaussian distributions for each OS subtype. Polar plots illustrate the mean (+1 SD) normalized response profiles for each OS subtype. (C–E) Degree of orientation selectivity (quantified by the OSI) across voxels with DSI < 0.5 and R^2^ > 0 in *tenm3*^*+*^ ACs (blue, n = 20 larvae), BCs (green, n = 20 larvae), and RGCs (red, n = 23 larvae; data from Figure 1H). Boxplots in (C) indicate interquartile ranges (boxes), medians (lines in boxes), and 10–90 percentiles (whiskers). The black dotted lines in (D) and (E) indicate the OSI threshold used to identify OS responses (OSI > 0.5). (F) Bar histogram summarizing the frequency of OS cells among *tenm3*^*+*^ ACs in 4-dpf *Tg(tenm3:Gal4)* larvae injected with *UAS:GCaMP6f* DNA constructs (n = 29 cells from 27 larvae). The number of observed OS cells for each *tenm3*^*+*^ AC type is reported at the bottom. nf, not found. (G) Scatterplot representing the relationship between OSI and dendritic field eccentricity of OS type II and III *tenm3*^*+*^ ACs (II, n = 5 cells; III, n = 3 cell). Spearman’s correlation coefficient (r) with the corresponding p value is reported. Dotted line represents the linear regression fit to the data. (H) Scatterplot representing the relationship between preferred stimulus orientation and dendritic field orientation of OS type II and III *tenm3*^*+*^ ACs (II, n = 5 cells; III, n = 3 cell). Spearman’s correlation coefficient (r) with the corresponding p value is reported. Dotted reference line indicates x = y. Top graph shows the angular difference between preferred stimulus orientation and dendritic field orientation (mean ± SD). (I) Normalized frequency distributions of preferred stimulus orientations in OS *tenm3*^*+*^ ACs (blue, n = 20 larvae), BCs (green, n = 20 larvae), and RGCs (red, n = 23 larvae; data from Figure 1J). The Gaussian distributions of the four different OS subpopulations are reported in separate graphs. (J) Relative proportions of the four different OS subpopulations (Pop 1–4) in *tenm3*^*+*^ ACs (blue), BCs (green), and RGCs (red). Values are obtained by calculating the relative proportion (%) of the area under the normalized Gaussian curves in (I). (K) Correlation matrix showing Spearman’s correlation coefficients (r) between the frequency distribution of the four OS subpopulations in *tenm3*^*+*^ ACs, BCs, and RGCs. (L) Two-photon functional calcium imaging of BC ribbon synapses expressing SyGCaMP6 (green) in 4-dpf *Tg(−1.8ctbp2:SyGCaMP6)* larvae. Distance of the eye from LCD screen is 2 cm. Recordings are performed from two to four Z-planes (approximately 20 μm total volume thickness). Scale bar, 20 μm. (M) Cumulative histogram summarizing the incidence of preferred angles for identified BC OS voxels in 4-dpf larvae (n = 20 larvae). Overlaid curves are the fitted Gaussian distributions for each OS subtype. Polar plots illustrate the mean (+1 SD) normalized response profiles for each OS subtype. (N–P) Examples of visual responses to moving gratings from two BC terminals. Images showing the mean fluorescence across tuning experiments with identified regions of interest (ROIs) are reported in (N). (O) shows calcium responses of the two ROIs in (N) with black traces representing the average responses across three trials (gray traces) for each stimulus epoch. Polar plots in (P) illustrate the mean response profile (±SD, dotted lines) of each ROI with corresponding OSI, DSI, and R^2^ values. Note that ROI #1 shows orientation selectivity. Scale bar, 5 μm. See also Figures S6 and S7, Table S1, and Movies S4 and S5.

To analyze tuning in the *tenm3*^*+*^ AC population, we performed in vivo two-photon calcium imaging in the retinae of *Tg(tenm3:Gal4;UAS:SyGCaMP3)* larvae (Figure 6A; see Supplemental Experimental Procedures). We found that *tenm3*^*+*^ ACs show stimulus-locked responses to moving square-wave gratings (Movie S4). Notably, analyses using different metrics of orientation selectivity (i.e., OSI and circular variance) and progressively higher tuning stringency levels revealed a large fraction of *tenm3*^*+*^ ACs tuned to elongated stimuli (Figures S6A–S6D). The distribution of preferred stimulus orientations across *tenm3*^*+*^ ACs indicated the presence of four subpopulations of OS responses tuned to gratings oriented along the cardinal (13°, 90°) and diagonal axes (40°, 145°; Figure 6B), similar to what we observed in OSGCs (Figure 1J). Compared to RGCs, however, *tenm3*^*+*^ ACs exhibited a higher degree of orientation selectivity (Figures 6C–6E). If activation of *tenm3*^*+*^ ACs along a particular axis in the visual field leads to inhibition of OSGC responses along that axis, one would expect that the relationship between their population distributions is inversely correlated. We therefore analyzed the frequency distribution of the four OS subpopulations in *tenm3*^*+*^ ACs and RGCs (Figures 6I and 6J). We found that they are indeed anti-correlated (Figure 6K), suggesting that the OS inhibitory input provided by *tenm3*^*+*^ ACs to OSGCs is orthogonally tuned (i.e., tuned to the orientation orthogonal to the OSGC-preferred orientation).

Given the presence of different *tenm3*^*+*^ AC types (Figure 5), we asked which ones display high orientation selectivity. We thus performed functional imaging of individually GCaMP6f-labeled *tenm3*^*+*^ ACs, followed by analyses of their tuning and dendritic field morphology (Figure S7A). Strikingly, the only *tenm3*^*+*^ ACs that showed stimulus-locked visual responses characterized by high orientation tuning were type II or III ACs (Figure 6F). Their degree of orientation selectivity was correlated with the elongation of their dendritic fields (Figure 6G), and the angular difference between their preferred stimulus orientation and dendritic field orientation was close to zero (Figure 6H), indicating that type II and III *tenm3*^*+*^ ACs respond maximally when the stimulus orientation coincides with the orientation of their dendritic fields. Additionally, the distribution of dendritic field orientations across sparsely eGFP-labeled type II and III *tenm3*^*+*^ ACs revealed that they fully cover the orientation space (Figures S7B and S7C). Since blocking GABA_A_ receptors leads to impaired RGC orientation selectivity (Figures 4G–4L), we investigated whether these two AC types are GABAergic by performing anti-GABA immunostaining of sparsely eGFP-labeled *tenm3*^*+*^ ACs. We observed that both type II and III *tenm3*^*+*^ ACs do indeed express the neurotransmitter GABA (Figures S7D and S7E), consistent with the results in Figure 4. Together, these data show that type II and III *tenm3*^*+*^ ACs constitute a source of orientation-tuned GABAergic inhibition in the retina.

### Orientation-Selective Responses in Bipolar Cell Presynaptic Terminals

Since ACs have been shown to modulate BC output at the level of individual presynaptic terminals [37], tuned inhibitory input from type II and III *tenm3*^*+*^ ACs could potentially generate orientation tuning in BC presynaptic terminals. We started investigating this idea by performing calcium imaging in the retinae of *Tg(−1.8ctbp2:SyGCaMP6)* larvae, where BC ribbon synapses are selectively labeled with SyGCaMP6 (Figure 6L; Movie S5) [38]. Interestingly, we observed that a fraction of BC responses (∼5% of visually responsive voxels) is indeed highly orientation selective (OSI > 0.5, DSI < 0.5, R^2^ > 0.8; Figures 6N–6P and S6E–S6H). Similarly to OSGCs and *tenm3*^*+*^ ACs, the preferred stimulus orientations of OS responses fall into four subpopulations tuned to gratings oriented along the cardinal (18°, 99°) and diagonal axes (44°, 149°; Figure 6M). The degree of orientation selectivity across the whole population of BC terminals appeared to be more similar to RGCs than *tenm3*^*+*^ ACs (Figures 6C–6E). Furthermore, the frequency distribution of the four OS BC subpopulations is highly correlated with that of OSGCs but inversely correlated with *tenm3*^*+*^ ACs (Figures 6I–6K). This therefore suggests that orientation selectivity in BC terminals could be generated by orthogonal orientation-tuned inhibitory input from *tenm3*^*+*^ ACs, similarly to OSGCs.

## Discussion

The vertebrate retina extracts information from visual scenes and sends it to higher brain areas through feature-specific neural pathways. Crucial neural substrates underlying this information processing in the retina are the stereotypic synaptic connections between defined neural cell types. How specific elements of the retinal circuit perform computations is, however, largely unknown. The data presented here define cellular and molecular building blocks of a circuit in the larval zebrafish retina capable of detecting the orientation of elongated visual stimuli. In particular, we take advantage of the functional link between RGC orientation selectivity and the cell-adhesion molecule Tenm3 to genetically identify a class of ACs with elongated dendritic arbors that show orientation tuning. We reveal that these *tenm3*^*+*^ ACs and their GABAergic inhibitory output are crucial for the tuning of orientation-selective RGCs. Moreover, we show that Tenm3 is a key molecular player in both the morphological and functional development of the circuit, and that orientation selectivity is also present among bipolar cell presynaptic terminals. Our study represents, to our knowledge, the most extensive characterization of the retinal orientation-selective circuit in a single tractable system. By collecting functional and structural data from amacrine, bipolar, and ganglion cells at cellular and population levels, we provide a mechanistic explanation of how defined neural cell types in the retina generate a fundamental property of visual perception—i.e., orientation selectivity. Additionally, our results elucidate the functional role of two novel AC types, therefore shedding some light on the most diverse and least understood retinal cell class [39].

### A Circuit Model of Orientation Selectivity in the Retina

**Figure 7:**
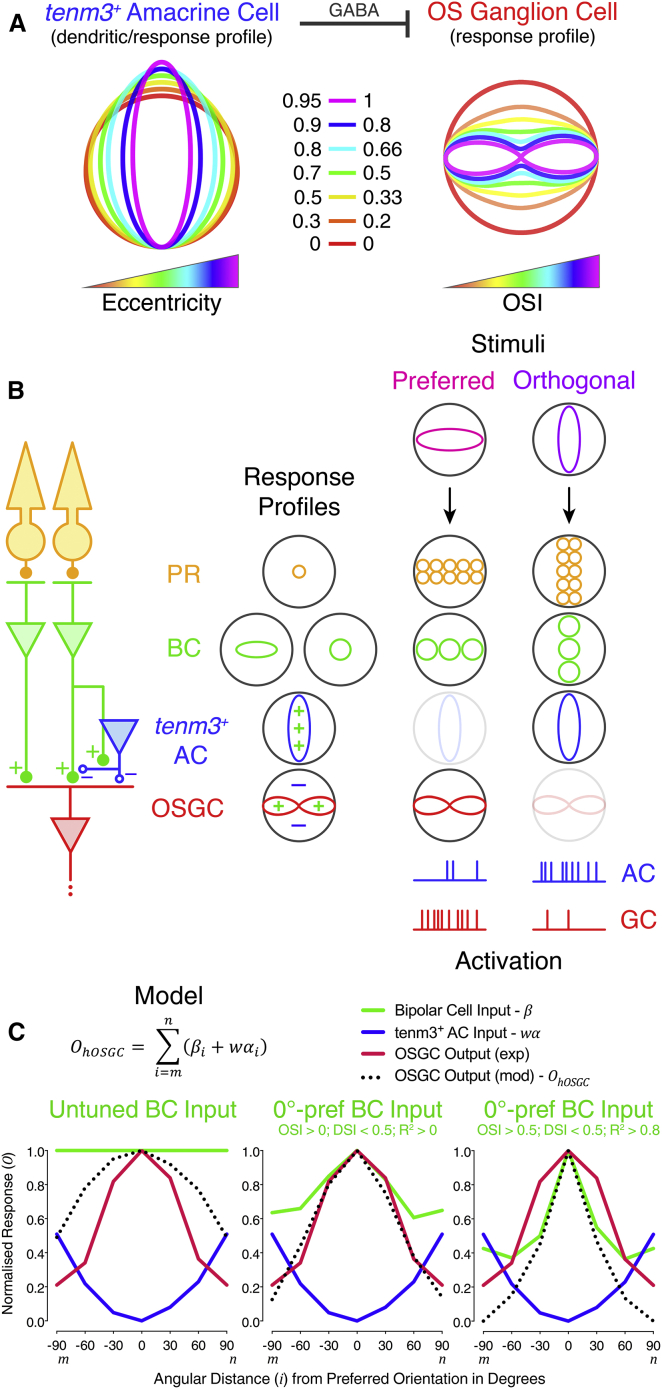
Circuit Model of Orientation Selectivity in the Retina (A) Hypothesized principles underlying the emergence of orientation selectivity in the retina. The high dendritic field elongation (quantified by the eccentricity of fitted elliptic profiles) of defined *tenm3*^*+*^ AC types is at the basis of their high orientation tuning (left). Maximal activation of tuned AC types is obtained when the orientation of elongated visual stimuli coincides with the orientation of their dendritic fields. As a result, these tuned *tenm3*^*+*^ AC types generate orientation selectivity in RGCs (quantified by the OSI, right) by providing orthogonal orientation-tuned GABAergic inhibitory input. The color code describes the different levels of dendritic field elongation (left) and orientation selectivity (right). (B) Examples of retinal OS circuit activation patterns for horizontal orientation-tuned OSGC preferred (magenta) and orthogonal (purple) stimuli. Excitatory input is indicated by “+” (full circles), whereas inhibitory input is indicated by “–” (empty circles). Putative synapses between OS *tenm3*^*+*^ ACs and BC terminals are also represented. Tuning profiles of example photoreceptor (PR), bipolar cells (BCs), OS *tenm3*^*+*^ amacrine cell (AC), and orientation-selective ganglion cell (OSGC) are also reported. (C) Simulation of the OSGC tuning profile (*Ο*_*hOSGC*_, black dotted line) using experimentally observed average response profiles of orthogonally tuned OS *tenm3*^*+*^ ACs (α, blue line; n = 20 larvae) and BC terminals (β, green line; n = 20 larvae). Three different orientation-tuning levels of excitatory BC input were used: untuned (left), weakly tuned to preferred orientation (middle) and highly tuned to preferred orientation (right). The experimentally observed average response profile of OSGCs (n = 23 larvae) is shown in red. The algorithm used for the simulation is reported at the top with the related legend. Note that, since the OS *tenm3*^*+*^ AC input (α) is inhibitory, a negative synaptic weight factor (*w*) is used in the algorithm. The orientation space ranges from “*m*” to “*n*”, which are negative (−90°) and positive (90°) angles orthogonal to the preferred orientation (0°), respectively. Exp, experimental; mod, model; pref, preferred. See also Figure S5.

To integrate our data into a general framework describing the computation of orientation selectivity in RGCs, we outlined a model of the retinal OS circuit. The model is based on the following principles: (1) the highly elongated dendritic fields of type II and III *tenm3*^*+*^ ACs identified in this study (Figure 5) underlie their orientation tuning (Figures 6 and 7A). Specifically, these defined AC types respond maximally when the orientation of elongated visual stimuli coincides with the orientation of their dendritic fields. Interestingly, ACs characterized by elongated dendritic fields and orientation selectivity have been found also in the rabbit retina, although their genetic identity is still unknown [13, 14]. (2) Type II and III *tenm3*^*+*^ ACs provide orthogonal orientation-tuned inhibitory input to OSGCs and, potentially, BC presynaptic terminals (Figures 6I–6K). This feedforward inhibition is mediated by GABA and generates orientation selectivity in OSGCs (Figures 3, 4, and 7A). Interestingly, pharmacological block of synaptic inhibition onto zebrafish BC terminals indicates that orientation selectivity in BC ribbon synapses is generated through AC inhibitory input (J. Johnston and L. Lagnado, personal communication), therefore supporting the idea that the OS inhibitory output of type II and III *tenm3*^*+*^ ACs could be at the basis of orientation selectivity in both BCs and OSGCs. Studies in the rabbit and mouse retina showed that OSGCs receive preferred orientation-tuned excitatory input and orthogonal orientation-tuned inhibitory input and, in rabbit, presynaptic GABAergic inhibition plays a pivotal role in the emergence of these OS inputs [9, 40]. Recent findings in *Drosophila* showed an analogous requirement of GABA signaling for orientation selectivity [5], revealing strikingly similar mechanisms between vertebrates and invertebrates. (3) Stimulus orientation, not the axis of stimulus movement, is the visual feature OSGCs are selective to. This is supported by our observation that static gratings, even though less effective in eliciting RGC responses, produce results analogous to those obtained using moving gratings (Figures S5G–S5J). Again, such property has been observed in rabbit and mouse OSGCs as well [10, 40]. Additional mechanisms to those described here may contribute to the emergence of RGC orientation selectivity.

In a schematic example of our model (Figure 7B), when the retina is stimulated with the OSGC-preferred stimulus orientation, the orthogonally tuned *tenm3*^*+*^ AC is weakly activated, therefore allowing the OSGC to fire action potentials. When the orthogonal stimulus orientation is presented, instead, the orthogonally tuned *tenm3*^*+*^ AC is strongly activated, and, consequently, OSGC firing is inhibited. To further evaluate our model, we implemented the basic principles described above into a simple simulation of OSGC output (Figure 7C). To simulate the OSGC tuning profile (black dotted line), we used our experimentally observed average response profiles of OS *tenm3*^*+*^ ACs (blue line) and BC presynaptic terminals (green line). We assumed the OS inhibitory input provided by *tenm3*^*+*^ ACs has a subtractive effect on OSGC output and tested three different orientation-tuning levels of excitatory BC input. Interestingly, we found that the average OSGC response profile observed experimentally (red line) was best reproduced when linearly integrating highly OS (OSI > 0.5, DSI < 0.5, R^2^ > 0.8) orthogonal orientation-tuned inhibitory input from *tenm3*^*+*^ ACs and weakly OS (OSI > 0, DSI < 0.5, R^2^ > 0) preferred orientation-tuned excitatory input from BCs (Figure 7C), indicating that OSGCs may receive BC input characterized by a substantial degree of heterogeneity in orientation tuning. This simulation also implies that OSGCs potentially integrate tuned input from both ACs and BCs to obtain the orientation selectivity observed in vivo. Our results show that the tuned GABAergic inhibitory output of *tenm3*^*+*^ ACs is necessary to generate normal RGC orientation selectivity. However, further experiments are needed to precisely determine the relative contribution played by inhibitory AC versus excitatory BC tuned inputs in OSGCs. The strong similarities found between the OS circuit we characterize in the zebrafish retina and previous descriptions of orientation selectivity in mammalian retinae [9, 13, 40] suggest that our model can be generalized to other vertebrate species.

### Functional Significance of Orientation Selectivity

The widespread presence of orientation-selective cells in visual systems of many animals highlights the prominent functional role of orientation selectivity in visual perception. Studies on the statistical properties of natural scenes indicate that natural images can be described by local, oriented filters similar to the receptive fields of OS cells found in visual systems [41]. One striking example in humans is the key role played by horizontal visual information in the identification of faces [42]. However, a central question is, where does orientation selectivity emerge in visual circuits? Interestingly, both in vertebrates and invertebrates the detection of elongated visual stimuli takes place early in visual processing [5, 10]. Even in mammalian species, including mice and monkeys, where for long time it was thought that orientation selectivity is an emergent property generated in primary visual cortex [1, 43], OS cells have been found in non-cortical areas such as the lateral geniculate nucleus [6, 7, 8] and superior colliculus [44], as well as in the retina [11, 12, 40, 45].

In our study, we found orientation selectivity in presynaptic terminals of BCs and ACs (Figures 6 and S6), which are neurons only one and two synapses away from photoreceptors, respectively. Additionally, we observed OS responses in these cells as early as 4 dpf, when zebrafish larvae start performing visually guided behaviors, such as the optokinetic reflex. Importantly, our data show that the cells and mechanisms underlying RGC orientation selectivity are different from those generating direction selectivity, in line with the notion that parallel retinal circuits process these two distinct visual features. This idea is further supported by the fact that zebrafish OSGC and DSGC axonal projections terminate in different, non-overlapping neuropil laminae of the optic tectum [4, 29].

### Role of Teneurins in Neural Circuit Wiring

Teneurins are phylogenetically conserved type II transmembrane proteins with large extracellular domains that are highly expressed in neural tissues [24, 25]. In vertebrates, the teneurin family comprises four members, Tenm1–4, whereas in invertebrates fewer members have been identified (one in *C. elegans*, two in *Drosophila*). In both vertebrate and invertebrate species, teneurins interact in *trans* through both homo- and heterophilic mechanisms [22, 23, 26, 27]. Notably, these trans-interactions are crucial in mediating cell-cell recognition and adhesion. Elegant studies in *Drosophila* demonstrated that teneurins play an instructive role in the synaptic matching between specific pre- and postsynaptic cells [22, 23]. In addition, teneurins regulate other fine-scale neural wiring processes in vivo, such as cell-type-specific dendrite morphogenesis [17, 46], synapse organization [23, 47], and axon projection topography and lamination [17, 48]. The precise roles played by homo- versus heterophilic trans-interactions during these wiring events are still unclear. However, it appears that homophilic interactions are crucial for the initial recognition and matching between specific subsets of neurons [22, 26], whereas heterophilic interactions are involved in subsequent steps of synapse adhesion and organization [27, 28, 47]. Since teneurins can control these distinct processes even between the same sets of neurons [22, 47], sophisticated genetic manipulations will be required to disentangle the contribution of homo- versus heterophilic trans-interactions in neurons where a given teneurin and its heterophilic binding partners are simultaneously expressed.

Our data suggest that Tenm3 specifies the correct development of functionally and morphologically defined subsets of ACs and RGCs forming a circuit underlying retinal orientation selectivity. Even though our results are suggestive of direct synaptic matching between *tenm3*^*+*^ ACs and OSGCs, the technical limitations of our study do not allow to unequivocally demonstrate the physical synaptic connections between these two neural populations, and, therefore, future experiments will be required to further elucidate this point. Given that Tenm3 is expressed in both ACs and RGCs (Figures S1C–S1F, S2B, and S2C) [17] and that Tenm3 loss of function leads to defects in *tenm3*^*+*^ AC neurite IPL stratification (Figures 2B–2D) as well as specific morphological and functional impairments in RGCs (Figures 1 and S1) [17], one possible explanation of Tenm3 mechanism of action could be through *trans*-synaptic homophilic interactions. However, loss of selective trans-interactions with other cell-adhesion molecules known to bind heterophilically with teneurins, such as latrophilins [27, 28], may as well explain the phenotypes we observed in *tenm3*^*KO*^ mutants. Interestingly, some latrophilins are expressed in the zebrafish eye at larval and adult stages, although it is not clear whether they exhibit a cell-type-specific expression pattern [49, 50]. Thus, the retinal orientation-selective circuit characterized in this study represents a tractable in vivo vertebrate system to test the specific roles played by teneurin homo- and heterophilic trans-interactions during neural circuit wiring.

In conclusion, our findings constitute a significant advancement in the understanding of how orientation selectivity emerges in the vertebrate retina, bringing together molecular markers, cell morphologies, pharmacology, and function. Moreover, the in vivo system and relative genetic tools established in this study will allow investigations of the precise functional role played by retinal orientation selectivity in higher visual areas of the brain as well as its role in performing visually guided behaviors.

## Experimental Procedures

Statistical test results are reported in the figures and figure legends. Statistical analyses and tests were carried out using Prism 6 (GraphPad), SigmaPlot 11 (Systat Software), or MATLAB R2014b (MathWorks). A comprehensive description of the statistical analyses and tests performed in this study can be found in Table S1. Before performing statistical tests, descriptive statistics (e.g., normality tests to see whether values come from a Gaussian distribution or F-test to compare variances) were used to choose the appropriate statistical test (reported in Table S1). The criterion for statistical significance was set at p < 0.05. In order to quantitatively measure and assess the effects of treatments or genetic manipulations between animal groups, the effect size (Cohen’s *d*) and its 95% confidence interval were also calculated (see Table S1). See Supplemental Experimental Procedures for detailed methods and zebrafish lines used in this study. All animal procedures were approved by the local Animal Welfare and Ethics Review Body (King’s College London) and were carried out in accordance with the Animals (Scientific Procedures) Act 1986 under license from the United Kingdom Home Office.

## Author Contributions

P.A. and R.H. designed the study. P.A. performed the experiments and analyzed the data. O.S. and C.M. generated the TALEN mutant zebrafish. P.A. and R.H. wrote the manuscript.

## Supplemental Information

Document S1. Figures S1–S7 and Supplemental Experimental Procedures

Table S1. Summary of Statistical Analyses Used in the Study, Related to Figures 1, 3, 4, 5, 6, S1, and S5

Movie S1. RGC Visual Responses to Moving Bars in Control and tenm3KO 4-dpf Zebrafish Larvae, Related to Figure 1 Time-lapse functional calcium imaging of representative control (top) and tenm3KO (bottom) 4 dpf Tg(isl2b:Gal4;UAS:SyGCaMP3) larvae. The movies encompass entire tuning experiments during which bars moving in 12 directions plus a blank screen null stimulus are presented to the right eye of immobilised zebrafish larvae. Unprocessed SyGCaMP3 responses are shown on the left, whereas ΔF/F0 calcium responses are reported on the right. The orientation of stimuli (°) is displayed when bars are projected onto the screen. Time is given in min:sec. The acquisition rate is 4.1 Hz, and the movie speed is approximately 5x. Anterior is up, posterior is down.

Movie S2. Tenm3^+^ ACs in the Retina of a 4-dpf Zebrafish Larva, Related to Figure 2 Z-stack showing the tenm3+ AC population in the retina of a 4 dpf Tg(tenm3:Gal4;UAS:tagRFPCAAX) larva. Relative depth is displayed in μm (46 μm total volume thickness with 1 μm Z resolution). Outer retina is up, inner retina is down.

Movie S3. RGC Visual Responses to Moving Bars in Control and tenm3^+^ AC-Ablated 4-dpf Zebrafish Larvae, Related to Figure 3 Time-lapse functional calcium imaging of representative control (top) and *tenm3+* AC-ablated (bottom) 4 dpf *Tg(tenm3:Gal4;UAS:KillerRed;elavl3:GCaMP5G)* larvae. The movies encompass entire tuning experiments during which bars moving in 12 directions plus a blank screen null stimulus are presented to the right eye of immobilised zebrafish larvae. Unprocessed GCaMP5G responses are shown on the left, whereas ΔF/F0 calcium responses are reported on the right. The orientation of stimuli (°) is displayed when bars are projected onto the screen. Local application of the glutamate receptor antagonists APV and NBQX (100 μM and 20 μM, respectively) in the optic tectum effectively blocks tectal cell calcium responses, therefore isolating RGC axonal responses. Time is given in min:sec. The acquisition rate is 4.1 Hz, and the movie speed is approximately 5x. Anterior is up, posterior is down.

Movie S4. Visual Responses to Moving Gratings of Tenm3^+^ AC Synaptic Terminals in a 4-dpf Zebrafish Larva, Related to Figure 6 Time-lapse two-photon calcium imaging of a representative 4 dpf *Tg(tenm3:Gal4;UAS:SyGCaMP3)* larva. The movie encompasses an entire tuning experiment during which gratings moving in 12 directions plus a null stimulus are presented to one eye of the immobilised zebrafish larva. SyGCaMP3 fluorescence responses are shown at the top, whereas ΔF/F0 calcium responses are reported at the bottom. The orientation of stimuli (°) is reported when gratings are displayed on the LCD screen. Time is given in min:sec. The acquisition rate is 7.8 Hz, and the movie speed is approximately 5x.

Movie S5. Visual Responses to Moving Gratings of BC Ribbon Synapses in a 4-dpf Zebrafish Larva, Related to Figure 6 Time-lapse two-photon calcium imaging of a representative 4 dpf *Tg(-1.8ctbp2:SyGCaMP6)* larva. The movie encompasses an entire tuning experiment during which gratings moving in 12 directions plus a null stimulus are presented to one eye of the immobilised zebrafish larva. SyGCaMP6 fluorescence responses are shown at the top, whereas ΔF/F0 calcium responses are reported at the bottom. The orientation of stimuli (°) is reported when gratings are displayed on the LCD screen. Time is given in min:sec. The acquisition rate is 7.8 Hz, and the movie speed is approximately 5x.

Document S2. Article plus Supplemental Information
