# Supplementary material for: Neural Mechanisms Generating Orientation Selectivity in the Retina
Source: Curr Biol. 2016 Jul 25;26(14):1802–15. doi: 10.1016/j.cub.2016.05.035 (PMC4963213; doi:10.1016/j.cub.2016.05.035)
Supplement: Document S1. Figures S1–S7 and Supplemental Experimental Procedures [file mmc1.pdf]

Current Biology, Volume *26*

Supplemental Information

Neural Mechanisms Generating

Orientation Selectivity in the Retina

Paride Antinucci, Oniz Suleyman, Clinton Monfries, and Robert Hindges

SUPPLEMENTAL FIGURES AND LEGENDS

Figure S1

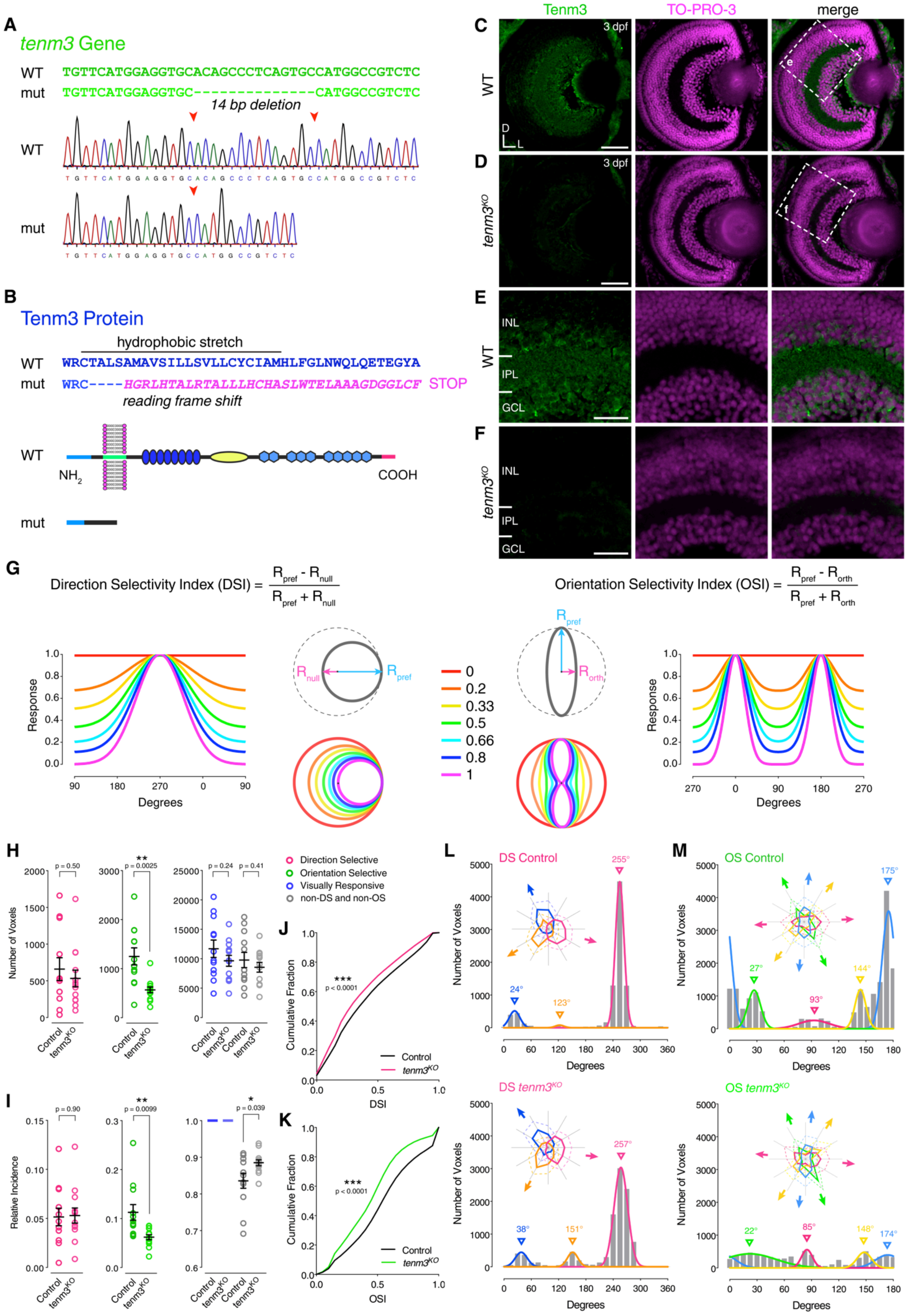

Figure S1. Tenm3 Knock-out, Quantification of Direction and Orientation Selectivity, and Tenm3 Functional Role in Late Development, Related to Figure 1 and Movie S1

(A and B) TALEN-mediated *tenm3* gene knock-out (A) and consequent structural changes in the Tenm3 protein (B). DNA sequencing chromatograms show the 14-base pair (bp) deletion present in *tenm3*^{KO} mutant (mut) larvae. This deletion in the gene region encoding the transmembrane domain of Tenm3 generates a reading frame shift and subsequent premature stop codon, therefore leading to the production of a non-functional Tenm3 protein missing its entire extracellular domain.

(C and D) Immunostaining showing the expression of Tenm3 (green) in retinae of 3-dpf WT (C) and *tenm3*^{KO} larvae (D). Cell bodies are labelled with the nuclear stain TO-PRO-3 (magenta). D, dorsal; L, lateral. Scale bars are 40 μm.

(E and F) Insets in (C) and (D) showing that, in the WT retina (E), Tenm3 is expressed in the inner nuclear layer (INL) and ganglion cell layer (GCL), whereas no Tenm3 expression is present in the *tenm3*^{KO} retina (F). IPL, inner plexiform layer. Scale bars are 20 μm. Importantly, all images in (C-F) were obtained using the same acquisition settings.

(G) Diagram illustrating the quantification of direction selectivity (left) and orientation selectivity (right) using the direction selectivity index (DSI) and orientation selectivity index (OSI), respectively. The algorithms used to calculate DSI and OSI of visual responses are reported at the top. For DSI, the responses to the preferred direction of moving bars (R_{pref}) and opposite, or null, direction (R_{null}; 180° angular distance) are used. For OSI, the responses to the preferred axis (R_{pref}) and orthogonal axis (R_{orth}; 90° angular distance) are used. Note that the arrows indicate the motion direction of orthogonally oriented bars or gratings. A single Gaussian or von-Mises distribution is used to fit responses of direction-selective cells and estimate their preferred direction of motion from the centre of the fitted curve. The sum of two Gaussian or von-Mises distributions (180° angular distance apart) is used to fit responses of orientation-selective cells in order to estimate their preferred axis and stimulus orientation from the centres of the fitted curves. The colour code describes the different levels of direction (left) and orientation (right) selectivity.

(H and I) Average number (H) and relative frequency (I) of DS, OS, visually responsive and non-DS/non-OS voxels per Z-plane in control (n = 12 larvae) and *tenm3*^{KO} (n = 13 larvae) 7-dpf *Tg(isl2b:Gal4;UAS:SyGCaMP3)* larvae. Error bars are ± SEM. *$p < 0.05$, **$p < 0.01$, unpaired two-tailed Student's t test.

(J and K) Cumulative distributions of DSI values ($R^2 > 0$) across voxels with OSI < 0.5 (J) and OSI values ($R^2 > 0$) across voxels with DSI < 0.5 (K) in control and *tenm3*^{KO} larvae. ***$p < 0.001$, two-sample Kolmogorov-Smirnov test.

(L and M) Cumulative histograms summarising the incidence of preferred angles for identified DS (L) and OS voxels (M) in control (n = 12; top) and *tenm3*^{KO} (n = 13; bottom) 7-dpf larvae. Overlaid curves are the fitted Gaussian distributions for each DS or OS subtype. Polar plots illustrate the mean (+ 1 SD) normalised response profiles for each DS or OS subtype.

Figure S2

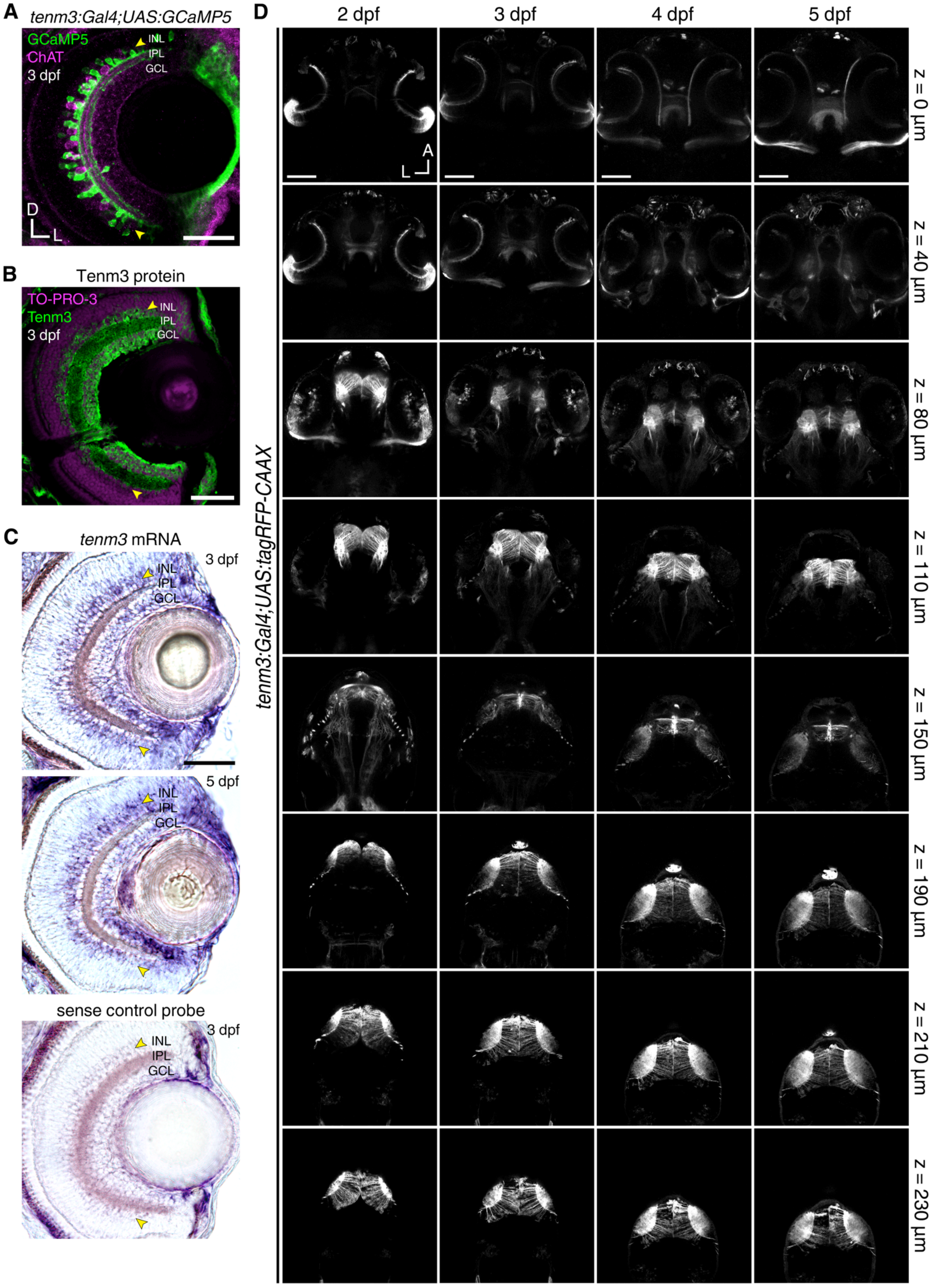
A
tenm3:Gal4;UAS:GCaMP5
GCaMP5
ChAT
3 dpf
INL
IPL
GCL
D
L
B
Tenm3 protein
TO-PRO-3
Tenm3
3 dpf
INL
IPL
GCL
C
tenm3 mRNA
3 dpf
INL
IPL
GCL
5 dpf
INL
IPL
GCL
sense control probe
3 dpf
INL
IPL
GCL
D
2 dpf
3 dpf
4 dpf
5 dpf
A
L
tenm3:Gal4;UAS:tagRFP-CAAX
z = 0 µm
z = 40 µm
z = 80 µm
z = 110 µm
z = 150 µm
z = 190 µm
z = 210 µm
z = 230 µm

Figure S2. ***Tg(tenm3:Gal4)*** **BAC Transgenic Labelling in the Retina and Brain, Related to Figure 2 and Movie S2**

(A) Immunostaining showing *tenm3* BAC transgenic labelling of a subset of amacrine cells in a 3-dpf *Tg(tenm3:Gal4;UAS:GCaMP5G)* larva. GCaMP5 expression is showed in green, whereas choline acetyltransferase (ChAT) expression is showed in magenta as a reference. Yellow arrowheads indicate *tenm3*$^{+}$ amacrine cells in the inner portion of the inner nuclear layer (INL). GCL, ganglion cell layer; IPL, inner plexiform layer. D, dorsal; L, lateral. Scale bar is 40 μm.

(B) Immunostaining showing the expression of Tenm3 (green) in the retina of a 3-dpf WT larva. Cell bodies are labelled with the nuclear stain TO-PRO-3 (magenta). Yellow arrowheads indicate Tenm3^{+} amacrine cells in the inner portion of the INL. Scale bar is 40 μm.

(C) *In situ* hybridisations showing *tenm3* mRNA expression in the retinae of 3- and 5-dpf WT larvae (top). A control *in situ* hybridisation at 3 dpf using sense *tenm3* riboprobe is reported at the bottom. Yellow arrowheads indicate the inner portion of the INL. Scale bar is 40 μm. From Antinucci, et al. [S1].

(D) Overview of the brain regions labelled by the *Tg(tenm3:Gal4;UAS:tagRFP-CAAX)* BAC transgenic zebrafish line from 2 to 5 dpf. Eight Z-planes are displayed per developmental stage (approximately 230 μm total volume thickness). The most ventral regions (z = 0 μm) are displayed at the top, whereas the most dorsal ones (z = 230 μm) are at the bottom. Note that tagRFP-CAAX is expressed in the retina (0-80 μm Z-axis depth), thalamus (80-150 μm Z-axis depth), and optic tectum (150-230 μm Z-axis depth), which are brain regions where *tenm3* is highly expressed. A, anterior; L, left. Scale bars are 100 μm.

Figure S3

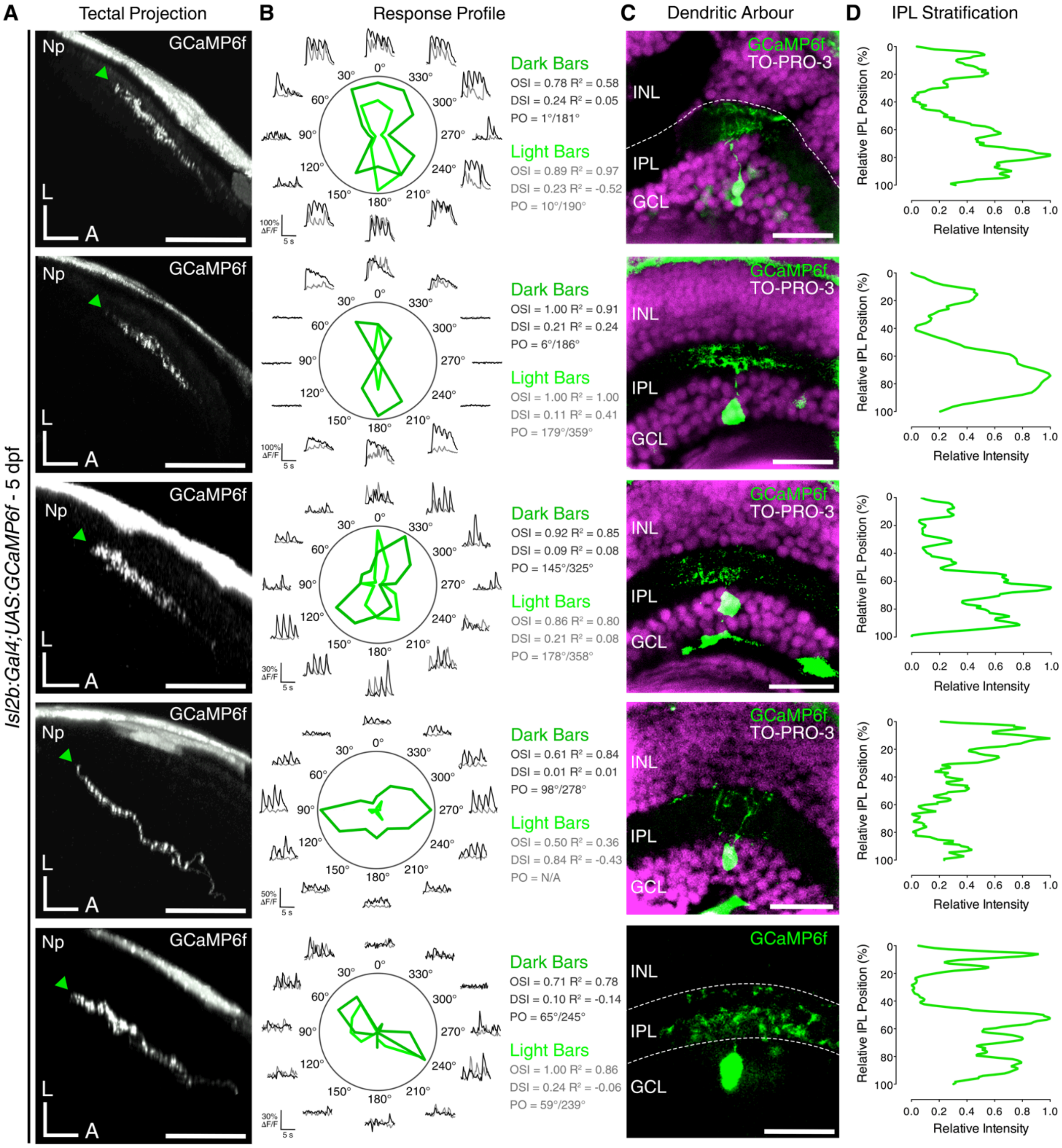

A
Tectal Projection
B
Response Profile
C
Dendritic Arbour
D
IPL Stratification
Isl2b:Gal4;UAS:GCaMP6f - 5 dpf
Np
GCaMP6f
L
A
Dark Bars
OSI = 0.78 R² = 0.58
DSI = 0.24 R² = 0.05
PO = 1°/181°
Light Bars
OSI = 0.89 R² = 0.97
DSI = 0.23 R² = -0.52
PO = 10°/190°
Dark Bars
OSI = 1.00 R² = 0.91
DSI = 0.21 R² = 0.24
PO = 6°/186°
Light Bars
OSI = 1.00 R² = 1.00
DSI = 0.11 R² = 0.41
PO = 179°/359°
Dark Bars
OSI = 0.92 R² = 0.85
DSI = 0.09 R² = 0.08
PO = 145°/325°
Light Bars
OSI = 0.86 R² = 0.80
DSI = 0.21 R² = 0.08
PO = 178°/358°
Dark Bars
OSI = 0.61 R² = 0.84
DSI = 0.01 R² = 0.01
PO = 98°/278°
Light Bars
OSI = 0.50 R² = 0.36
DSI = 0.84 R² = -0.43
PO = N/A
Dark Bars
OSI = 0.71 R² = 0.78
DSI = 0.10 R² = -0.14
PO = 65°/245°
Light Bars
OSI = 1.00 R² = 0.86
DSI = 0.24 R² = -0.06
PO = 59°/239°
0°
30°
60°
90°
120°
150°
180°
210°
240°
270°
300°
330°
100% ΔF/F
50% ΔF/F
30% ΔF/F
5 s
GCaMP6f
TO-PRO-3
INL
IPL
GCL
Relative IPL Position (%)
Relative Intensity

Figure S3. IPL Dendritic Stratification Pattern of Individual OSGCs, Related to Figure 2

(A) Side views of axon projections (green arrowheads) of individual OSGCs expressing GCaMP6f in the optic tectum of 5-dpf *UAS:GCaMP6f*-injected *Tg(isl2b:Gal4)* larvae. 12.8% of functionally imaged RGCs were OS (5 out of 39 cells in 39 larvae). Np, neuropil; A, anterior; L, lateral. Scale bars are 40 μm.

(B) Functional identification of individual OSGCs through analyses of their visual responses to moving bars. Polar plots represent the tuning profiles (obtained by calculating the integral response to each stimulus) of OSGCs for dark and light moving bars (dark and light green, respectively). Black and grey traces represent the $\Delta F/F_0$ calcium responses to moving dark and light bars, respectively. OSI, orientation selectivity index; DSI, direction selectivity index; R^2, Gaussian goodness of fit; PO, preferred orientation.

(C and D) Immunostaining for GCaMP6f (green) showing the dendritic morphology of functionally identified OSGCs (C) and corresponding normalised IPL fluorescence intensity profiles (D). 0% corresponds to the INL/IPL boundary, whereas 100% corresponds to the IPL/GCL boundary. Cell bodies are labelled with the nuclear stain TO-PRO-3 (magenta). INL, inner nuclear layer; GCL, ganglion cell layer; IPL, inner plexiform layer. Scale bars are 20 μm.

Figure S4

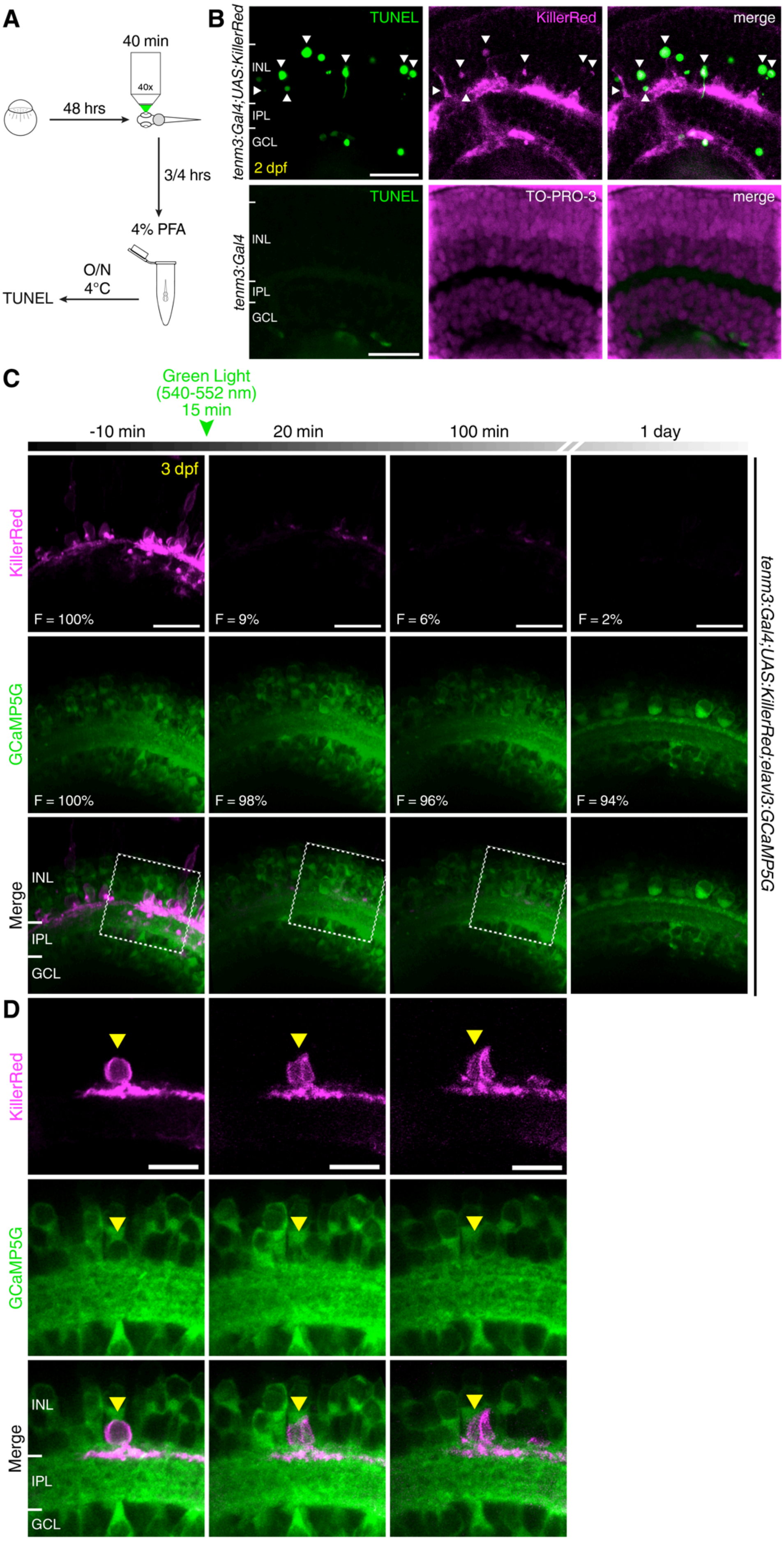
A
40 min
40x
48 hrs
3/4 hrs
4% PFA
O/N
4°C
TUNEL
B
tenm3:Gal4;UAS:KillerRed
TUNEL
KillerRed
merge
INL
IPL
GCL
2 dpf
tenm3:Gal4
TUNEL
TO-PRO-3
merge
C
Green Light
(540-552 nm)
15 min
-10 min
20 min
100 min
1 day
3 dpf
KillerRed
F = 100%
F = 9%
F = 6%
F = 2%
GCaMP5G
F = 100%
F = 98%
F = 96%
F = 94%
Merge
tenm3:Gal4;UAS:KillerRed;elavl3:GCaMP5G
D
KillerRed
GCaMP5G
Merge
INL
IPL
GCL

Figure S4. Optogenetic Ablation of Tenm3$^+$ ACs using KillerRed, Related to Figure 3 and Movie S3

(A) Schematic summarising the experimental procedure used to validate the optogenetic ablation of *tenm3*$^+$ ACs. At 2 dpf, the eyes of *Tg(tenm3:Gal4;UAS:KillerRed)* and *Tg(tenm3:Gal4)* control larvae were illuminated with intense green light (540-552 nm) for 40 minutes using a wide-field fluorescence microscope equipped with a 40× objective. After 3-4 hours, the larvae were fixed in 4% paraformaldehyde (PFA) at 4°C overnight (O/N). Subsequently, larvae were cryosectioned and fluorometric terminal deoxynucleotidyl transferase dUTP nick end labeling (TUNEL) assays were performed on sections following standard protocols.

(B) TUNEL staining (green) shows the presence of apoptotic nuclei characteristic of dying cells in the retinae of 2-dpf *Tg(tenm3:Gal4;UAS:KillerRed)* larvae following 40 minutes of green light illumination (top, n = 3 larvae). In these larvae KillerRed (magenta) is selectively expressed in *tenm3*$^+$ ACs. White arrowheads indicate the sites of co-localisation between KillerRed$^+$ cell bodies and TUNEL$^+$ apoptotic nuclei in the inner nuclear layer (INL). Importantly, no cell death is detected in the retinae of 2-dpf *Tg(tenm3:Gal4)* control larvae that were subjected the same illumination procedure (bottom, n = 3 larvae). In the bottom panel, cell bodies are labelled with the nuclear stain TO-PRO-3 (magenta). GCL, ganglion cell layer; IPL, inner plexiform layer. Scale bars are 20 μm.

(C) Proof-of-principle experiment showing the effects of 15 minutes illumination with green light on the KillerRed (magenta) fluorescence level (F) in the retina of a 3-dpf *Tg(tenm3:Gal4; UAS:KillerRed;elavl3:GCaMP5G)* larva. Strikingly, strong photobleaching (~90% decrease in fluorescence) is observed after green light illumination, indicating effective KillerRed phototoxicity. This large decrease in KillerRed fluorescence is visible even one day after illumination. No significant decrease in the overall GCaMP5G fluorescence (green) is detected after illumination. Importantly, all images were obtained using the same acquisition settings. Scale bars are 20 μm.

(D) Inset of (C) showing the dramatic morphological changes of a single KillerRed-expressing *tenm3*$^+$ AC (yellow arrowhead) following green light illumination. Notably, also the GCaMP5G fluorescence appears to decrease in this cell, probably due to the production of phototoxic reactive oxygen species in the cytoplasm. The magenta channel signal has been increased post-acquisition to compensate for the ~90% decrease in fluorescence resulting from the illumination procedure. Scale bars are 10 μm.

Figure S5

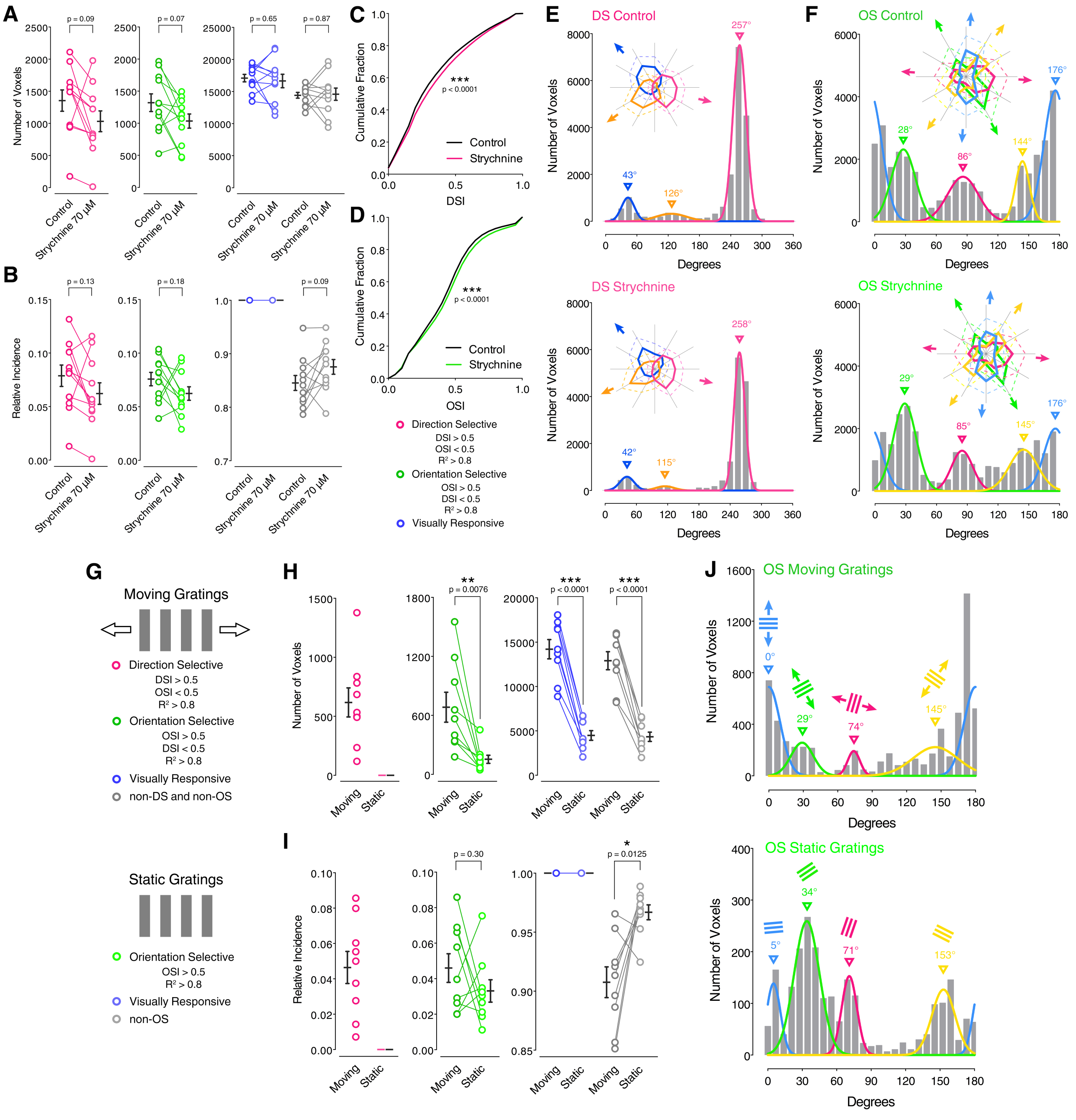
A
B
C
D
E
F
G
H
I
J
Number of Voxels
Relative Incidence
Control
Strychnine 70 µM
p = 0.09
p = 0.07
p = 0.65
p = 0.87
p = 0.13
p = 0.18
p = 0.09
Cumulative Fraction
DSI
OSI
***
p < 0.0001
Control
Strychnine
Direction Selective
DSI > 0.5
OSI < 0.5
R² > 0.8
Orientation Selective
OSI > 0.5
DSI < 0.5
R² > 0.8
Visually Responsive
DS Control
DS Strychnine
OS Control
OS Strychnine
Degrees
257°
43°
126°
258°
42°
115°
28°
86°
144°
176°
29°
85°
145°
176°
Moving Gratings
non-DS and non-OS
Static Gratings
non-OS
Moving
Static
**
p = 0.0076
p < 0.0001
p = 0.30
*
p = 0.0125
OS Moving Gratings
OS Static Gratings
0°
29°
74°
145°
5°
34°
71°
153°

Figure S5. Role of Glycinergic Inhibitory Input in Generating RGC Orientation Selectivity, and RGC Orientation-selective Responses Using Moving or Static Gratings, Related to Figure 4

(A and B) Average number (A) and relative frequency (B) of DS, OS, visually responsive and non-DS/non-OS voxels per Z-plane in 5-dpf *Tg(isl2b:Gal4;UAS:SyGCaMP3)* larvae (n = 11 larvae) before (control) and after (strychnine) the application of strychnine (70 µM). Error bars are ± SEM. Paired two-tailed Student's t test.

(C and D) Cumulative distributions of DSI values ($R^2 > 0$) across voxels with OSI < 0.5 (C) and OSI values ($R^2 > 0$) across voxels with DSI < 0.5 (D) before (control) and after (strychnine) the application of strychnine (70 µM). ***p < 0.001, two-sample Kolmogorov-Smirnov test.

(E and F) Cumulative histograms summarising the incidence of preferred angles for identified DS (E) and OS voxels (F) in 5-dpf larvae (n = 11 larvae) before (control) and after (strychnine) the application of strychnine (70 µM). Overlaid curves are the fitted Gaussian distributions for each DS or OS subtype. Polar plots illustrate the mean (+ 1 SD) normalised response profiles for each DS or OS subtype.

(G) Diagram showing the criteria used to identify DS and OS voxels when using moving (top) or static gratings (bottom). The DSI cannot be calculated when using static stimuli, therefore only OS, visually responsive and non-OS voxels can be identified when larvae are stimulated with static gratings.

(H and I) Average number (H) and relative frequency (I) of DS, OS, visually responsive and non-DS/non-OS RGC voxels per Z-plane in 5-dpf *Tg(isl2b:Gal4;UAS:SyGCaMP3)* larvae (n = 9 larvae) using either moving or static gratings. Individual larvae are tested using both stimuli. Error bars are ± SEM. *p < 0.05, **p < 0.01, ***p < 0.001, paired two-tailed Student's t test.

(J) Cumulative histograms summarising the incidence of preferred angles for identified OS voxels in 5-dpf larvae (n = 9 larvae) using either moving (left) or static gratings (right). Overlaid curves are the fitted Gaussian distributions for each OS subtype, with the colour-coded bars indicating the preferred orientation of gratings. Note that, in both stimulus groups, OS populations with similar preferred angles are present, indicating that OSGCs are tuned to the stimulus orientation not its axis of movement.

Figure S6

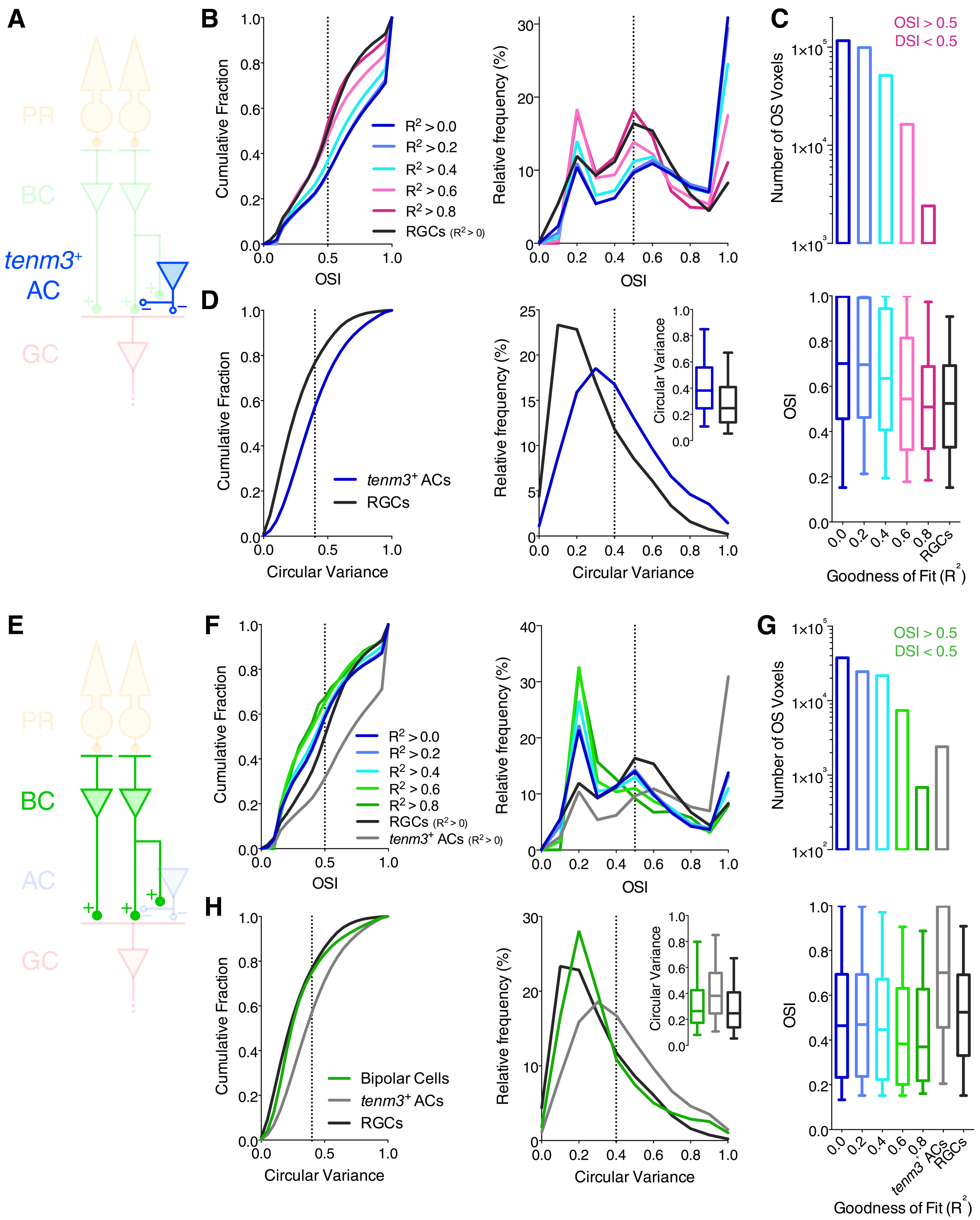

A
PR
BC
tenm3+ AC
GC
B
Cumulative Fraction
OSI
R2 > 0.0
R2 > 0.2
R2 > 0.4
R2 > 0.6
R2 > 0.8
RGCs (R2 > 0)
Relative frequency (%)
C
OSI > 0.5
DSI < 0.5
Number of OS Voxels
D
tenm3+ ACs
RGCs
Circular Variance
Relative frequency (%)
OSI
Goodness of Fit (R2)
RGCs
E
PR
BC
AC
GC
F
R2 > 0.0
R2 > 0.2
R2 > 0.4
R2 > 0.6
R2 > 0.8
RGCs (R2 > 0)
tenm3+ ACs (R2 > 0)
G
OSI > 0.5
DSI < 0.5
H
Bipolar Cells
tenm3+ ACs
RGCs
Circular Variance
tenm3+ ACs
Goodness of Fit (R2)

Figure S6. Orientation Selectivity in Tenm3+ ACs and BC Ribbon synapses, Related to Figure 6 and Movies S4 and S5

(A) Selective recording of *tenm3*+ AC synaptic terminals through two-photon calcium imaging in 4-dpf *Tg(tenm3:Gal4;UAS:SyGCaMP3)* larvae.

(B) Cumulative distribution (left) and relative frequency (right) of orientation selectivity index (OSI) values across voxels with DSI < 0.5 in *tenm3*+ ACs (n = 20 larvae) and RGCs (black, n = 23 larvae; data from Figure 1H). The colour code indicates the progressively higher goodness of fit (R^2 of the fitted von-Mises profiles) criteria applied to the data in order to identify voxels characterised by high orientation selectivity. The black dotted lines indicate the OSI threshold used to identify OS responses (OSI > 0.5). Depending on the R^2 value chosen as threshold, the number of voxels that satisfy the criteria, and therefore are represented in the distributions, varies.

(C) Total number of OS voxels (top) and distribution of OSI values (bottom) using progressively higher R^2 values in *tenm3*+ ACs and RGCs (black, $R^2 > 0$). Boxplots indicate interquartile ranges (boxes), medians (lines in boxes) and 10-90 percentiles (whiskers).

(D) Cumulative distribution (left) and relative frequency (right) of circular variance values across all voxels in *tenm3*+ ACs (blue, n = 20 larvae) and RGCs (black, n = 23 larvae). The black dotted lines indicate the circular variance threshold used to identify OS responses (circular variance < 0.4). Boxplots in the right inset indicate interquartile ranges (boxes), medians (lines in boxes) and 5-95 percentiles (whiskers).

(E) Selective recording of BC ribbon synapses through two-photon calcium imaging in 4-dpf *Tg(-1.8ctbp2:SyGCaMP6)* larvae.

(F) Cumulative distribution (left) and relative frequency (right) of orientation selectivity index (OSI) values across voxels with DSI < 0.5 in BCs (n = 20 larvae), *tenm3*+ ACs (grey, n = 20 larvae) and RGCs (black, n = 23 larvae; data from Figure 1H). The colour code indicates the progressively higher goodness of fit (R^2 of the fitted von-Mises profiles) criteria applied to the data in order to identify voxels characterised by high orientation selectivity. The black dotted lines indicate the OSI threshold used to identify OS responses (OSI > 0.5). Depending on the R^2 value chosen as threshold, the number of voxels that satisfy the criteria, and therefore are represented in the distributions, varies.

(G) Total number of OS voxels (top) and distribution of OSI values (bottom) using progressively higher R^2 values in BCs, *tenm3*+ ACs (grey, $R^2 > 0$) and RGCs (black, $R^2 > 0$). Boxplots indicate interquartile ranges (boxes), medians (lines in boxes) and 10-90 percentiles (whiskers).

(H) Cumulative distribution (left) and relative frequency (right) of circular variance values across all voxels in BCs (green, n = 20 larvae), *tenm3*+ ACs (grey, n = 20 larvae) and RGCs (black, n = 23 larvae). The black dotted lines indicate the circular variance threshold used to identify OS responses (circular variance < 0.4). Boxplots in the right inset indicate interquartile ranges (boxes), medians (lines in boxes) and 5-95 percentiles (whiskers).

Figure S7

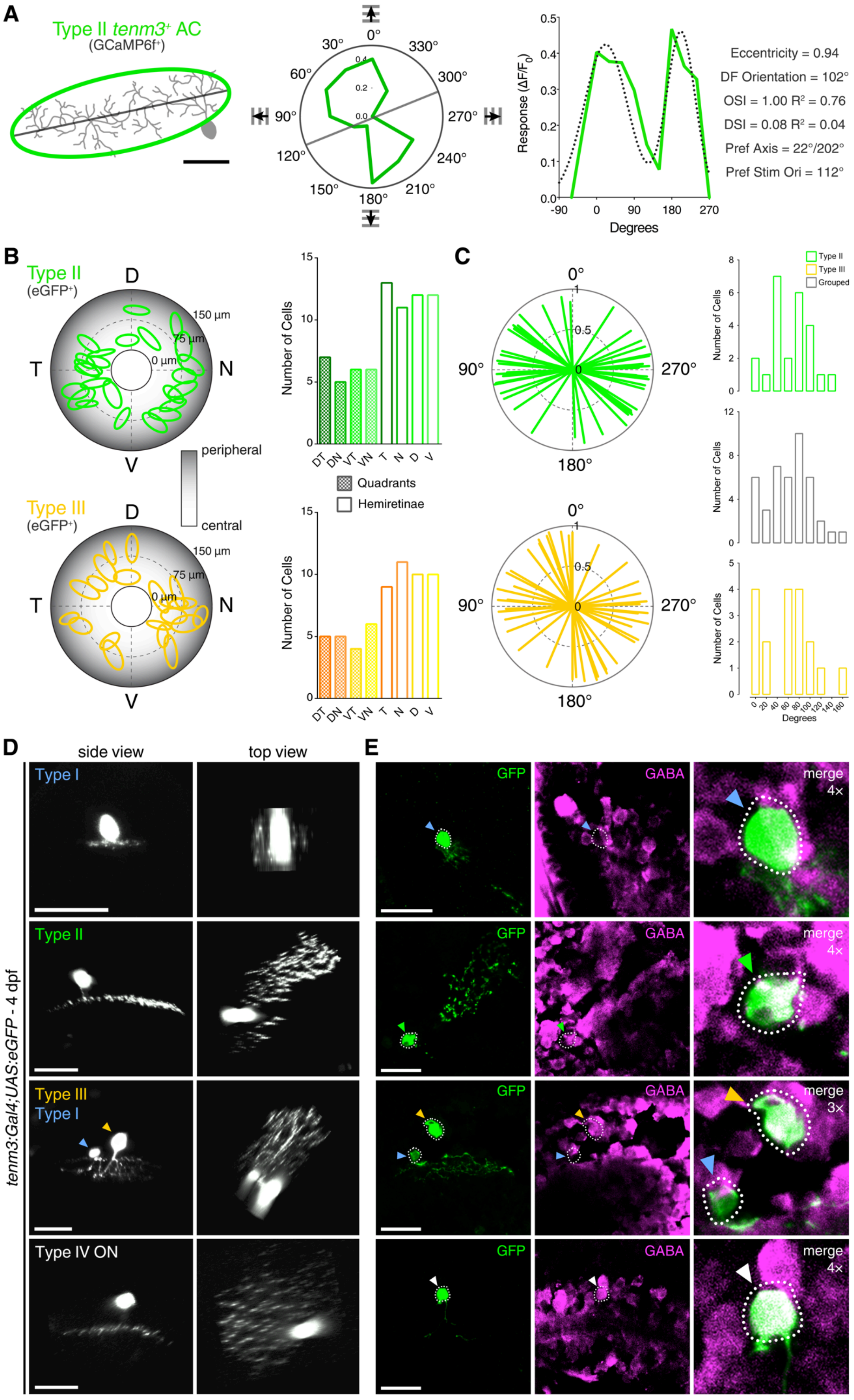
A
Type II tenm3+ AC
(GCaMP6f+)
Eccentricity = 0.94
DF Orientation = 102°
OSI = 1.00 R² = 0.76
DSI = 0.08 R² = 0.04
Pref Axis = 22°/202°
Pref Stim Ori = 112°
Response (ΔF/F0)
Degrees
B
Type II
(eGFP+)
Type III
(eGFP+)
D
T
N
V
150 µm
75 µm
0 µm
peripheral
central
Number of Cells
Quadrants
Hemiretinae
DT DN VT VN T N D V
C
Type II
Type III
Grouped
Degrees
D
side view
top view
tenm3:Gal4;UAS:eGFP - 4 dpf
Type I
Type II
Type III
Type IV ON
E
GFP
GABA
merge
4×
3×

Figure S7. Functional Imaging, Dendritic Field Orientation and Neurotransmitter Identity of Single Tenm3$^+$ ACs, Related to Figure 6

(A) Summary of morphological and functional analyses of single GCaMP6f-labelled *tenm3*$^+$ ACs in 4-dpf *UAS:GCaMP6f*-injected *Tg(tenm3:Gal4)* larvae. An example of a type II *tenm3*$^+$ AC that showed stimulus-locked responses and high orientation selectivity is displayed. Left: morphological reconstruction of the cell (grey, top view) with corresponding fitted ellipse profile (green) and dendritic field (DF) orientation (black line). Scale bar is 20 μm. Middle: Polar plot representing the tuning profile of the cell (green, obtained by calculating the integral response to each stimulus) in response to gratings moving in different directions. Grey line indicates preferred stimulus orientation (Pref Stim Ori). Radial axis scale shows $\Delta F/F_0$ of integral calcium responses. Right: Tuning profile (green) with fitted sum of two Gaussians (dotted black line). Measurements of morphological and functional characteristics of the example cell are reported on the right. R^2, goodness of fit of the sum of two Gaussians distribution.

(B) Retinal location mapping of individual eGFP-CAAX-labelled type II (green, top) and type III (yellow, bottom) *tenm3*$^+$ ACs in 4-dpf *UAS:eGFP-CAAX*-injected *Tg(tenm3:Gal4)* larvae (n = 24 and 20 cells in 39 larvae, respectively). Frequencies of type II and III *tenm3*$^+$ ACs in number of cells per retinal quadrant or hemiretina are reported on the right. D, dorsal; V, ventral; N, nasal; T, temporal.

(C) Grouped data summarising dendritic field orientation and elongation (quantified by the eccentricity of dendritic fields; radial axis) of individual eGFP-CAAX-labelled type II (green, top) and type III (yellow, bottom) *tenm3*$^+$ ACs (n = 24 and 20 cells in 39 larvae, respectively). Radial axis scale of the polar plots shows the eccentricity of dendritic fields. Frequencies of type II and III *tenm3*$^+$ ACs in number of cells per orientation (20° bin width; 0°-180° orientation space) are reported on the right.

(D) Mosaically labelled *tenm3*$^+$ ACs expressing eGFP in 4-dpf *UAS:eGFP*-injected *Tg(tenm3:Gal4)* larvae. Both side views (left) and top views (right) are shown. Scale bars are 20 μm.

(E) Immunostaining showing the expression of γ-aminobutyric acid (GABA; magenta) in the identified eGFP-expressing *tenm3*$^+$ ACs (green) reported in (D). Arrowheads are colour-coded according to the *tenm3*$^+$ AC type each cell is classified into. Note that both type II (green arrowheads) and type III (yellow arrowheads) *tenm3*$^+$ ACs express the neurotransmitter GABA. Interestingly, type I *tenm3*$^+$ ACs (cyan arrowheads) do not appear to express GABA. Note that the merged images (right) have been enlarged by the reported magnification factors to help visualisation. Scale bars are 20 μm.

SUPPLEMENTAL EXPERIMENTAL PROCEDURES

Animals

Zebrafish were maintained at 28.5°C on a 14 hr ON/10 hr OFF light cycle in Danieau solution [58 mM NaCl, 0.7 mM KCl, 0.4 mM $MgSO_4$, 0.6 mM $Ca(NO_3)_2$ 5.0 mM HEPES, pH 7.6]. Transgenic lines used in this study include *Tg(isl2b:Gal4)* [S2], *Tg(UAS:SyGCaMP3)* [S3], *Tg(UAS:tagRFP-CAAX)* [S4], *Tg(UAS:KillerRed)* [S5], *Tg(elavl3:GCaMP5G)* [S6], *Tg(UAS:GCaMP5)* (gift from Dr. Elena Dreosti, Department of Cell and Developmental Biology, University College London, London, UK), and *Tg(-1.8ctbp2:SyGCaMP6)* [S7]. The *Tg(tenm3:Gal4)* zebrafish line was generated through BAC transgenic insertion (*kg302Tg*) by the authors (see *BAC Transgenesis*). The $tenm3^{KO}$ mutant line was generated through transcription activator-like effector nuclease (TALEN)-mediated genome editing ($tenm3^{kg69/kg69}$) from Ekkwill zebrafish by the authors (see *TALEN-mediated tenm3 Knock-out*). WT retinae used in Figures S1C-S1F and S2B were obtained from Ekkwill larvae.

Confocal functional imaging experiments were performed in the pigmentation mutant, *nacre*, which lacks all neural crest-derived melanophores [S8]. Two-photon functional imaging experiments in the retina were performed in a triple pigmentation mutant ($alb^{b4/b4}$;$nacre^{w2/w2}$;$roy^{a9/a9}$) which is characterized by a general decrease in melanin synthesis ($alb^{b4/b4}$) [S9] and lacks all neural crest-derived melanophores ($nacre^{w2/w2}$) [S8] and iridophores ($roy^{a9/a9}$) [S10]. The rationale behind this strategy was to have an unobstructed optical access of the retina without raising fish in 200 µM phenylthiourea (PTU) [S11], which has been shown to impair both the normal development of zebrafish [S12, S13] and the retinal responses to light stimuli [S14]. Importantly, $alb^{b4/b4}$;$nacre^{w2/w2}$;$roy^{a9/a9}$ larvae and adult zebrafish are viable and do not display evident morphological, functional or behavioural abnormalities. Larvae used for *in vivo* and *ex vivo* imaging of morphological and molecular features (not involving functional experiments) were raised in 200 µM PTU (Sigma) in Danieau solution to avoid pigment formation. This work was approved by the local Animal Care and Use Committee (King's College London), and was carried out in accordance with the Animals (Experimental Procedures) Act, 1986, under license from the United Kingdom Home Office.

Genome Editing

TALEN-mediated tenm3 Knock-out

Zebrafish *tenm*3 knock-out mutants ($tenm3^{KO}$) were generated by TALEN-mediated genome editing. The exon encoding the transmembrane domain of the Tenm3 protein was targeted. The DNA sequences targeted by the TALEN constructs are the following (5'>3'): left, TCAAAGTACTGTTCATGG; right, GTGCCATGGCCGTCTCCA. TALEN constructs were designed using ZiFiT targeter version 4.2 [S15], and assembled following the REAL assembly protocol [S16]. The T7 transcription mMessage mMachine kit (Life Technologies) was used to produce the 5' capped TALEN mRNA, 200 pg of which was injected into Ekkwill zebrafish embryos at one-cell stage [S17]. Founders were identified using the Surveyor nuclease mismatch assay

(Transgenomic) and outcrossed to generate heterozygous F1 offspring, which were subsequently fin-clipped and genotyped by PCR amplifying and sequencing a 400 bp amplicon using primers that flanked the target site. The primers used for genotyping are the following (5'>3'): tenm3_fw CAGGCACTTTCTCTTTAAGACAGG; tenm3_rev CTCACGTGATCCTCTCTCAGC. Zebrafish were selected where we detected indels leading to frame shifts and consequent premature stop codons causing truncations in the transmembrane domain of Tenm3. The *tenm3*^{KO} mutant zebrafish line (*tenm3*$^{kg69/kg69}$) used for all subsequent experiments is characterized by a 14-bp deletion (5'-ACAGCCCTCAGTGC-3') in the following genomic location of *tenm3*: chromosome 1, 38,921,612-38,921,625.

BAC Transgenesis

The *Tg(tenm3:Gal4)* transgenic zebrafish line was generated through bacterial artificial chromosome (BAC) transgenic insertion (*kg302Tg*) following previously described methods [S18]. In summary, the recombineering procedure used to generate *tenm3:Gal4* BAC constructs consists of three steps. First, three different BAC clones (CH211-58M24, 200 kb; CH211-130I22, 155 kb; CH211-169K10, 150 kb; BACPAC Resources, CHORI) containing partially overlapping regions of the *tenm3* genomic sequence were transformed with the *pRedFlp4* plasmid. Second, the *iTol2* cassette with 50-bp homologies on each end (*iTol2-amp*) was inserted into *tenm3* BAC vector backbones. Third, the transcriptional activator Gal4FF gene together with a kanamycin resistance cassette possessing 50-bp homology arms (*Gal4FF-kan*) was inserted at the start ATG site of the *tenm3* gene (the exon containing the start ATG codon is in chromosome 1: 38,879,739-38,879,945).

To create first transient and subsequently stable transgenic zebrafish lines, isolated BAC DNA constructs (120 ng/μl) were co-injected with *Tol2* transposase mRNA (100 ng/μl) into *Tg(UAS:GCaMP5)* zebrafish embryos at the early one-cell stage. Transient expression, visible as GCaMP5 fluorescence, already gave a reliable indication of the final expression in the stable transgenic line. All three BAC constructs produced analogous transient expression patterns. The CH211-58M24 (200 kb) BAC construct was used to generate the *Tg(tenm3:Gal4)* zebrafish line because of its higher level of Gal4FF expression compared to the other two BAC constructs. The CH211-58M24 BAC construct contains genomic regions 100 kb upstream and 100 kb downstream of the *tenm3* start codon site, but does not cover the entire *tenm3* genomic locus (~400 kb). The BAC DNA preparation was obtained using the HiPure Midiprep kit (Invitrogen), with modifications for BAC DNA isolation as described by the manufacturer. *tol2* transposase mRNA was prepared by *in vitro* transcription from *Xba*I-linearized *pDB600* plasmid [S19] using the T3 transcription mMessage mMachine kit (Life Technologies). RNA was purified using the RNeasy MinElute Cleanup kit (Qiagen). Germ line transmission was identified by mating sexually mature adult fish to wild-type fish and, subsequently, examining their progeny for GCaMP5 fluorescence. Positive embryos were raised to adulthood and outcrossed to generate the *Tg(tenm3:Gal4)* single transgenic line, which was then used as driver line.

This is the list of primers used for recombineering (5’>3’).

**itol2 cassette amplification**	
ptarBac_itol2_fw	GCGTAAGCGGGGCACATTTCATTACCTCTTTCTCCGCACCCG ACATAGATCCCTGCTCGAGCCGGGCCCAAGTG
ptarBac_itol2_rev	CGCGGGGCATGACTATTGGCGCGCCGGATCGATCCTTAATTA AGTCTACTAATTATGATCCTCTAGATCAGATCT
**Gal4FF-Kan cassette amplification**	
tenm3BAC_Gal4FF_fw	ATTTTATTTTCTTCCTTTGGTTTAAAGTGATTTTTCTTTCTATAG GTTCTACCATGAAGCTACTGTCTTCTATCGAAC
tenm3BAC_KanR_rev	GGTTGAGAATGGGAATGCTCAGTGACTGATGGACTGGAGAG AGAGGATGGTCAGAAGAACTCGTCAAGAAGGCG
**itol2 cassette insertion control**	
pTarBAC_HA1_control_fw	CTGTCAAACATGAGAATTGGTC
amp_HA1_control_rev	ACATTTCCCCGAAAAGTGG
amp_HA2_control_fw	CTGAGATAGGTGCCTCACTG
pTarBAC_HA2_control_rev	GAGAGCCTTCAACCCAGTC
**Gal4FF-Kan cassette insertion control**	
tenm3BAC_control_fw	GCTACTCACCATTGCTGTGGCC
Gal4FF_HA1_control_rev	AGTAGCGACACTCCCAGTTG
kanR_HA2_control_fw	TCCTCGTGCTTTACGGTATC
tenm3BAC_control_rev	CGCCTGTGTGGTGGCATTAC

Imaging

Confocal Imaging

Imaging was performed using an LSM 710 confocal microscope equipped with a spectral detection scan head and a 20×/1.0 NA water-immersion objective (Carl Zeiss). Optical sections were obtained at 1 μm intervals through the Z axis. Maximum intensity projections and 3D rotated images were generated using either ImageJ [S20] or ZEN (Carl Zeiss).

In Vivo Calcium Imaging

<u>Confocal</u>

Functional time-series of visually evoked calcium responses in RGCs were acquired at a rate of 4.1 Hz and 0.415×0.415 μm resolution (256×256 pixels) and 1 AU pinhole aperture, using the confocal setup described above. The average diameter of a presynaptic bouton in zebrafish RGCs is ~0.8 μm. Thus, the physical X-Y dimensions of voxels are below that of a typical presynaptic bouton. Excitation was provided by 488 nm multi-line laser. Non-anaesthetised *Tg(isl2b:Gal4; UAS:SyGCaMP3)*, *Tg(tenm3:Gal4;UAS:KillerRed;elavl3:GCaMP5G)* and *UAS:GCaMP6f*-injected *Tg(isl2b:Gal4)* larvae were immobilized in 2% low melting point agarose (Sigma) prepared in Danieau solution and mounted dorsal side up on a raised glass platform that was placed in a custom-made Danieau-filled chamber. The agarose was sufficient to restrain the larvae so that anaesthesia was not required. Imaging was performed in the afternoon (1-8 pm).

Two-photon

Two-photon functional imaging in the retina was performed using an A1R MP microscope equipped with a 4-channel GaAsP NDD and an Apochromat 25×/1.1 NA water-immersion objective (Nikon). Excitation was provided by a Chameleon Ultra II Mode-locked titanium-sapphire laser (Coherent) tuned to 930 nm. Time-series of visually evoked calcium responses in *tenm3*$^{+}$ ACs and BCs were acquired at a rate of 7.8 Hz and 0.397×0.397 µm resolution (256×128 pixels). Following activation of the laser scanning, we waited 60 seconds before starting the visual stimulation to ensure the retina adapted to the background light level caused by the multi-photon laser. 4-dpf *Tg(tenm3:Gal4;UAS:SyGCaMP3)*, *Tg(-1.8ctbp2:SyGCaMP6)* and *UAS:GCaMP6f*-injected *Tg(tenm3:Gal4)* larvae were first paralysed for 10-15 minutes in α-bungarotoxin (1 mg/ml; Biotium) prepared in Danieau solution. Subsequently, larvae were immobilized in 2% low melting point agarose (Sigma) and mounted on a raised custom-made glass platform with the dorsal side up (45° angle) and one eye facing an LCD screen (see Visual Stimulation) that was placed underneath a custom-made Danieau-filled chamber. Imaging was performed in the afternoon (1-8 pm).

Visual Stimulation

Moving Bars and Moving vs. Static Gratings in Confocal Preparation

Moving bar stimuli were generated as previously described [S1, S21]. A diffusion filter (3026, Rosco) was bonded to one side of the chamber to serve as a projection screen. The agarose in front of the eye facing the projection screen was removed, allowing an unobstructed view of the projected image on the side of the chamber. Larvae were positioned 3 cm away from the screen, and the projected image filled a visual field of ~97° x 63°. Visual stimuli consisted of light (56 cd/m^{2}) or dark bars (8 cd/m^{2}) (175% and 25% of mean luminance, respectively) on a mean gray background (32 cd/m^{2}). As no qualitative differences between light and dark bars were noted, data obtained using the two stimuli were combined. Each bar was 10° in width moving at a speed of 20°/s and separated from the preceding bar by 30°, enabling more than one bar on the screen at any one time. The long axes of the bars were orthogonal to the direction of motion. For the moving vs. static gratings stimuli used to obtain data displayed in Figures S5G-S5J, either moving square-wave gratings (75% contrast, spatial frequency 0.05 cycles/degree equivalent to 10° bar width, temporal frequency 1 cycles/s) or static square-wave gratings with alternating contrast (75% contrast, spatial frequency 0.05 cycles/degree equivalent to 10° bar width) were used. Each of the 12 directions of motion was presented once (3 seconds) in a pseudo-random order unique to each slice in every animal imaged. Each inter-epoch interval was 10 seconds to enable SyGCaMP3 or GCaMP6f signals to return to baseline. A blank-screen null condition of 2 seconds was also interleaved. Visual experiments were generated and controlled using custom-written Labview and MATLAB code (MathWorks), implemented on a ViSaGe stimulus presenter (Cambridge Research Systems) and delivered via a DLP pico projector (Optoma).

Moving Gratings in Two-photon Preparation

Moving gratings stimuli in the two-photon preparation were generated and controlled using PsychoPy [S22], and delivered through an LCD screen (SKD5VA-3, GoodWell Technology) positioned underneath a custom-made Perspex chamber. A long-pass red glass filter (FGL610, Thorlabs) was positioned between the LCD screen and the chamber to allow for simultaneous imaging and visual stimulation. Larvae were positioned 2 cm away from the screen and the image on the LCD screen filled a visual field of ~140° x 100° (mean background luminance 30.4 cd/m^2). Visual stimuli consisted of square-wave gratings (100% contrast, spatial frequency 1.66 cycles/cm, temporal frequency 1 cycles/s). Each grating bar was 8.5° in width and the long axes of the bars were orthogonal to the direction of motion. Each of the 12 directions of motion was presented once (6 seconds) with and inter-epoch interval of 10 seconds to enable SyGCaMP3 or SyGCaMP6 signals to return to baseline. A blank-screen null condition of 6 seconds was also interleaved. TTL triggers (0-5-0 Volts) to record epoch time events where generated through a LabJack USB DAQ device (U3-LV, LabJack Corporation). Following activation of the laser scanning, we waited 60 seconds before starting the visual stimulation to ensure the retina adapted to the background light level caused by the multi-photon laser.

Single Cell Labelling

Labelling of Individual RGCs

To label individual RGCs, *UAS:GCaMP6f* DNA constructs [S23, S24] were injected into 1-4 cell-stage *Tg(isl2b:Gal4)* embryos. The *pGP-CMV-GCaMP6f* plasmid used to clone the *UAS:GCaMP6f* DNA construct [S24] was a gift from Douglas Kim (Addgene plasmid # 40755). Plasmid DNA was prepared using midiprep kits (Qiagen) and injected at a concentration of 30 ng/μl in Danieau solution. Only larvae showing single GCaMP6f-expressing RGC axons in the optic tectum were used for functional analyses. After functional identification of orientation-selective RGCs (OSGCs), larvae were fixed, cryosectioned and immunostained (see *Immunohistochemistry*) to visualize the morphology of GCaMP6f$^+$ dendritic arbours of OSGCs.

Mosaic Labelling of Tenm3$^+$ ACs

To mosaically label *tenm3*$^+$ ACs, *UAS:eGFP-CAAX* (gift from Dr. Andy Symonds, MRC Centre for Developmental Neurobiology, King's College London, London, UK), *UAS:eGFP* [S2] or *UAS:GCaMP6f* [S24] DNA constructs were injected into 1-4 cell-stage *Tg(tenm3:Gal4)* embryos. Plasmid DNA was prepared using midiprep kits (Qiagen) and injected at a concentration of 40-50 ng/μl in Danieau solution.

Optogenetic Ablation *of Tenm3$^+$ ACs*

The optogenetic ablation of *tenm3*$^+$ ACs was performed using the genetically encoded photosensitizer KillerRed [S5, S25] following previously described guidelines [S26, S27]. At 2 dpf, the eyes of KillerRed$^+$ *Tg(tenm3:Gal4;UAS:KillerRed;elavl3:GCaMP5G)* and *Tg(tenm3:Gal4;*

UAS:KillerRed) larvae, and KillerRed[-] *Tg(tenm3:Gal4;elavl3:GCaMP5G)* and *Tg(tenm3:Gal4)* control larvae were illuminated with continuous intense green light (540-552 nm; filter set 15, BP 546/12 nm) for 40 minutes using a wide-field fluorescence microscope (Zeiss Axioskop) equipped with a 40× objective and a 100 Watts mercury lamp employed at maximal light intensity and objective aperture. In KillerRed[+] larvae, strong photobleaching (~90% decrease in relative fluorescence) was observed after green light illumination, indicating effective KillerRed phototoxicity. To detect cell death, 3-4 hours following illumination the larvae were fixed in 4% paraformaldehyde (PFA) at 4°C overnight. Subsequently, larvae were cryosectioned and fluorometric terminal deoxynucleotidyl transferase dUTP nick end labeling (TUNEL; DeadEnd, Promega) assays were performed on sections following standard protocols.

Pharmacology

Block of Glutamate Receptors in the Optic Tectum

To isolate RGC axonal calcium responses from tectal cell dendritic responses in 4-dpf *Tg(tenm3:Gal4;UAS:KillerRed;elavl3:GCaMP5G)* and *Tg(tenm3:Gal4;elavl3:GCaMP5G)* larvae, we pressure-injected the NMDA and AMPA/kainate glutamate receptor antagonists D-2-amino-5-phosphonovaleric acid (APV; Sigma) and 2,3-dihydroxy-6-nitro-7-sulfamoyl-benzo[f]quinoxaline-2,3-dione (NBQX; Sigma), 100 μM and 20 μM in Danieau solution respectively, into one tectal hemisphere as previously described [S4]. Larvae were imaged immediately post-injection. Animals were only included in the analyses if visual responses were not detected in tectal cell bodies following pharmacological treatment.

$GABA_A$ Receptors Block

To block γ-aminobutyric acid (GABA)-mediated inhibition from amacrine cells, we used the $GABA_A$ receptor chloride channel blocker picrotoxin (Sigma) at a final concentration of 100 μM, as previously described [S28]. Functional calcium imaging was first performed in 4-dpf *Tg(isl2b:Gal4;UAS:SyGCaMP3)* larvae in Danieau solution with no drug. Subsequently, larvae were mounted in 2% low melting point agarose (Sigma) in Danieau solution with freshly made 100 μM picrotoxin and then imaged again. Importantly, also the Danieau solution in the imaging chamber contained 100 μM picrotoxin.

Glycine Receptors Block

To block glycine-mediated inhibition from amacrine cells, we used the glycine receptor antagonist strychnine (Sigma) at a final concentration of 70 μM [S29]. Functional calcium imaging was first performed in 5-dpf *Tg(isl2b:Gal4;UAS:SyGCaMP3)* larvae in Danieau solution with no drug. Subsequently, larvae were mounted in 2% low melting point agarose (Sigma) in Danieau solution with freshly made 70 μM strychnine and then imaged again. Importantly, also the Danieau solution in the imaging chamber contained 70 μM strychnine.

Immunohistochemistry

The primary antibodies used in the study were diluted in blocking solution as follows: chicken anti-GFP (1:500; Abcam, AB13970), rabbit anti-GABA (1:100; Sigma, A2052), guinea pig anti-GABA (1:500; Abcam, AB17413), mouse anti-Parvalbumin (1:300; Millipore, MAB1572), rabbit anti-TH (1:500; Millipore, AB152), goat anti-ChAT (1:100; Millipore, AB144P), rabbit anti-glycine (1:50; Abcam, AB9442). The primary antibody used to detect the Tenm3 protein in Figures S1C-S1F is a custom-made rabbit anti-tenm3 antibody (1:150 dilution in blocking solution; Eurogentec) designed to recognise the following protein sequence, 445-TIDTGEVDVGRRAVQ-459, which is located in the extracellular domain of the zebrafish Tenm3 protein. The secondary antibodies used in the study were goat or donkey anti-chicken, anti-mouse, anti-rabbit, anti-guinea pig and anti-goat conjugated to Alexa Fluor 488 or 568 fluorophores (1:1000 dilution in blocking solution; Life Technologies). Nuclei were counterstained with TO-PRO-3 Iodide (1:1000 dilution in blocking solution; Life Technologies, T3605).

Immunostainings of zebrafish larvae on cryosections were performed following standard procedures. Briefly, larvae were fixed in 4% PFA overnight, rehydrated with PBS for 3×10 minutes, cryoprotected in 15%, 30% sucrose at room temperature for 2 hours and, finally, 40% sucrose at 4°C overnight. Larvae used to immunostain Tenm3 in the retina were subjected to the following antigen retrieval procedure [S30]: the cryoprotected larvae were incubated in 150 mM Tris-HCl pH 9.0 at room temperature for 5 minutes, followed by heating at 70°C for 15 minutes and, finally, re-cryoprotected in 40% sucrose at 4°C overnight. The cryoprotected larvae were then embedded in molds using O.C.T. compound (VWR International) and cryosectioned (18 µm thickness). Cryosections were mounted on glass slides (Superfrost plus, Thermo Scientific) and dried overnight at room temperature. Subsequently, slides were equilibrated 3×5 minutes in PBST (0.1% Tween-20 in PBS) and then blocked with 3% goat or donkey serum + 1% BSA in PBST for 1 hour at room temperature. After blocking, the slides were incubated with the respective primary antibodies at 4°C overnight. Finally, slides were washed 3×30 minutes in PBST, incubated with the respective secondary antibodies at room temperature for 2-3 hours, and then washed 3×30 minutes in PBST. To preserve the immunostained cryosections, Fluoromount-G (eBioscience) was used. Slides were then covered with glass coverslips and stored at 4°C until use.

Analysis

Functional Analyses

In vivo calcium imaging data were analysed as previously described [S1, S21]. In summary, functional time-series were processed before analysis as follows: time-series images from each experiment were corrected for motion with a rigid-body algorithm (SPM12; http://www.fil.ion.ucl.ac.uk/spm/), median filtered with a kernel size of 1 voxel to remove dark and shot noise, and spatially smoothed with a 2D Gaussian kernel = 2 voxels to improve signal-to-noise. A baseline (B) that corrects for low-frequency drifts was determined using a cubic-spline algorithm extrapolating

between knots averaged from 5 s of the inter-epoch interval data. Both relative signal intensity changes (ΔF = F-B; where F = raw fluorescence) and normalised signal intensity changes [$\%\Delta F/F_0 = (F-B)/B$] were calculated at each voxel. ΔF was used for population functional data (voxel-wise analysis), whereas $\%\Delta F/F_0$ was used for single cell functional data (manually defined regions of interest, ROIs, of dendritic or axonal arbours of GCaMP6f-labelled cells). For each voxel or ROI the integral response over the epoch-interval was calculated to provide a single response metric of each presented direction of stimulus motion. The integral within each epoch window is a summary metric more resistant to saturation effects of the calcium probe than maximum signal change. A threshold for each voxel within an acquisition image sequence was determined from the variance of ΔF changes during the inter-epoch intervals and null condition, threshold = 5×SDs. All voxels that were supra-threshold within at least two visual presentation epochs were regarded as visually responsive and subjected to further characterization.

To analyse the direction and orientation selectivity of visually responsive voxels or ROIs, direction- and orientation-selective indices (DSI and OSI) [S31], based on fitted von-Mises or Gaussian profiles [S32], were calculated together with an estimate for their goodness of fit, R^2. The DSI was defined as $(R_{pref}-R_{null})/(R_{pref}+R_{null})$, where R_{pref}, the response to the preferred direction, was the integral response over the preferred direction epoch-interval. R_{null} was similarly calculated as the integral response evoked by the direction opposite to the preferred direction (180° angular distance). The OSI was defined as $(R_{pref}-R_{orth})/(R_{pref}+R_{orth})$, where R_{pref}, the response to the preferred orientation, was the integral response over the preferred orientation epoch-interval. R_{orth} was similarly calculated as the integral response evoked by the orientation orthogonal to the preferred orientation (90° angular distance). To minimize cross talk and over-fitting associated with DSI and OSI metrics, a stringent approach was undertaken. For a voxel or ROI to be regarded as direction-selective (DS) or orientation-selective (OS), mutually exclusive criteria were used: DS if DSI > 0.5 and OSI < 0.5; and OS if OSI > 0.5 and DSI < 0.5. In both cases, the goodness of fit (R^2) for DSI and OSI, respectively, had to be > 0.8; thus, the fitted curves explained at least 80% of the integral responses. A single von Mises distribution was used to fit responses of DS voxels and estimate their preferred direction of motion angle from the centre of the fitted curve, respectively. The sum of two von Mises or Gaussian distributions (180° angular distance apart) was used to fit responses of OS voxels or OS ROIs, respectively, and estimate their preferred orientation of motion angles from the centres of the fitted curves. Grouped cumulative histograms of the preferred direction or orientation of motion angles were generated from the calculated preferred angles of identified DS or OS voxel. The distribution of preferred angles for each identified DS or OS subpopulation of voxels was fitted by a Gaussian distribution with peak centre (mean), height (amplitude) and standard deviation being free dimensions. Circular variance was also calculated for comparison as an alternative metric of orientation selectivity (Circular variance < 0.4) [S33].

Morphological Analyses

To determine the IPL stratification profile of *tenm3*$^{+}$ AC neurites and OSGC dendrites, rectangular 10 µm-wide ROIs were drawn across the IPL. The Plot Profile function in ImageJ [S20] was applied to the ROIs to calculate fluorescence intensity traces across the IPL depth. The traces obtained from multiple larvae were then normalised and averaged using SigmaPlot (Systat Software). The dendritic field area and orientation angle of individual eGFP-CAAX- or GCaMP6f-labelled *tenm3*$^{+}$ ACs were measured using ImageJ [S20] in 3D rotated images generated through ZEN (Carl Zeiss). To quantify the dendritic field elongation of individual *tenm3*$^{+}$ ACs, we calculated the eccentricity [$e = \sqrt{(1-b^2/a^2)}$; a = length of the ellipse semi-major axis, b = length of the ellipse semi-minor axis] of dendritic arbour profiles following ellipse fitting in ImageJ [S20]. 3D morphological reconstructions of individual *tenm3*$^{+}$ ACs were obtained using the Simple Neurite Tracer plugin in FIJI [S34]. To estimate the total number of type I-IV *tenm3*$^{+}$ ACs required to tile the retina with a coverage factor of 1, the mean IPL area (49,903 ± 3,707 µm^2, mean ± SD; n = 14 retinae from 14 larvae at 4 dpf) was divided by the observed mean dendritic field area of each *tenm3*$^{+}$ AC type and, subsequently, the obtained values were used to estimate the respective relative frequency.

Statistical Analyses

Statistical analyses and tests were carried out using Prism 6 (GraphPad), SigmaPlot 11 (Systat Software) or MATLAB R2014b (MathWorks). A comprehensive description of the statistical analyses and tests performed in this study can be found in Table S1. Before performing statistical tests, descriptive statistics (e.g., normality tests to see whether values come from a Gaussian distribution or F-test to compare variances) were used to choose the appropriate statistical test (reported in Table S1). The criterion for statistical significance was set at $p < 0.05$. In order to quantitatively measure and assess the effects of treatments or genetic manipulations between animal groups, the effect size (Cohen's *d*) and its 95% confidence interval were also calculated (see Table S1). No statistical method was used to predetermine sample sizes, but our sample sizes are similar to those generally employed in the field and in most cases provided a high statistical power (i.e., power > 0.8).

Simulation of OSGC Output

To simulate the tuning profile of orientation-selective ganglion cells (OSGCs) we used the following algorithm:

$$O_{hOSGC} = \sum_{i=m}^{n} (\beta_i + w\alpha_i)$$

The tuning profile of OSGCs tuned to horizontal bars moving along the vertical axis (0°; hence, O_{hOSGC}) was simulated by summating normalised bipolar cell (BC) excitatory input (β) and OS *tenm3*$^{+}$ amacrine cell (AC) inhibitory input (α) for each stimulus orientation (i) across the whole orientation space. The orientation space ranges from ‘m’ to ‘n’, which are negative (-90°) and

positive (90°) angles orthogonal to the preferred orientation (0°), respectively. Values used for ‘α’ are the experimentally observed average responses of orthogonal orientation-tuned *tenm3*$^+$ ACs (i.e., *tenm3*$^+$ ACs tuned to vertical bars moving along the horizontal axis; OSI > 0.5, DSI < 0.5, R^2 > 0.8; n = 20 larvae). Note that, since the OS *tenm3*$^+$ AC input is inhibitory, a negative synaptic weight factor (w) is used in the algorithm. As a consequence, the OS inhibitory input provided by *tenm3*$^+$ ACs has a subtractive effect on OSGC output. Values used for ‘β’ are the experimentally observed average responses of BC terminals (n = 20 larvae). Three different orientation-tuning levels of excitatory BC input were tested, namely: a) untuned BC input (average responses of all visually responsive BC voxels), (b) weakly OS (OSI > 0, DSI < 0.5, R^2 > 0) preferred orientation-tuned BC input; c) highly OS (OSI > 0.5, DSI < 0.5, R^2 > 0.8) preferred orientation-tuned BC input. The experimentally observed average response profile of OSGCs tuned to horizontal bars moving along the vertical axis (n = 23 larvae) was used as a comparison to evaluate the accuracy of the simulated OSGC response profile.

SUPPLEMENTAL REFERENCES

S1. Antinucci, P., Nikolaou, N., Meyer, M.P., and Hindges, R. (2013). Teneurin-3 specifies morphological and functional connectivity of retinal ganglion cells in the vertebrate visual system. Cell Rep *5*, 582-592.

S2. Ben Fredj, N., Hammond, S., Otsuna, H., Chien, C.B., Burrone, J., and Meyer, M.P. (2010). Synaptic activity and activity-dependent competition regulates axon arbor maturation, growth arrest, and territory in the retinotectal projection. J Neurosci *30*, 10939-10951.

S3. Nikolaou, N., Lowe, A.S., Walker, A.S., Abbas, F., Hunter, P.R., Thompson, I.D., and Meyer, M.P. (2012). Parametric functional maps of visual inputs to the tectum. Neuron *76*, 317-324.

S4. Hunter, P.R., Lowe, A.S., Thompson, I.D., and Meyer, M.P. (2013). Emergent properties of the optic tectum revealed by population analysis of direction and orientation selectivity. J Neurosci *33*, 13940-13945.

S5. Del Bene, F., Wyart, C., Robles, E., Tran, A., Looger, L., Scott, E.K., Isacoff, E.Y., and Baier, H. (2010). Filtering of visual information in the tectum by an identified neural circuit. Science *330*, 669-673.

S6. Ahrens, M.B., Orger, M.B., Robson, D.N., Li, J.M., and Keller, P.J. (2013). Whole-brain functional imaging at cellular resolution using light-sheet microscopy. Nat Methods *10*, 413-420.

S7. Johnston, J., Ding, H., Seibel, S.H., Esposti, F., and Lagnado, L. (2014). Rapid mapping of visual receptive fields by filtered back projection: application to multi-neuronal electrophysiology and imaging. J Physiol *592*, 4839-4854.

S8. Lister, J.A., Robertson, C.P., Lepage, T., Johnson, S.L., and Raible, D.W. (1999). nacre encodes a zebrafish microphthalmia-related protein that regulates neural-crest-derived pigment cell fate. Development *126*, 3757-3767.

S9. Streisinger, G., Singer, F., Walker, C., Knauber, D., and Dower, N. (1986). Segregation analyses and gene-centromere distances in zebrafish. Genetics *112*, 311-319.

S10. White, R.M., Sessa, A., Burke, C., Bowman, T., LeBlanc, J., Ceol, C., Bourque, C., Dovey, M., Goessling, W., Burns, C.E., et al. (2008). Transparent adult zebrafish as a tool for in vivo transplantation analysis. Cell Stem Cell *2*, 183-189.

S11. Karlsson, J., von Hofsten, J., and Olsson, P.E. (2001). Generating transparent zebrafish: a refined method to improve detection of gene expression during embryonic development. Mar Biotechnol (NY) *3*, 522-527.

S12. Elsalini, O.A., and Rohr, K.B. (2003). Phenylthiourea disrupts thyroid function in developing zebrafish. Dev Genes Evol *212*, 593-598.

S13. Li, Z., Ptak, D., Zhang, L., Walls, E.K., Zhong, W., and Leung, Y.F. (2012). Phenylthiourea specifically reduces zebrafish eye size. PLoS One *7*, e40132.

S14. Page-McCaw, P.S., Chung, S.C., Muto, A., Roeser, T., Staub, W., Finger-Baier, K.C., Korenbrot, J.I., and Baier, H. (2004). Retinal network adaptation to bright light requires tyrosinase. Nat Neurosci *7*, 1329-1336.

S15. Sander, J.D., Maeder, M.L., Reyon, D., Voytas, D.F., Joung, J.K., and Dobbs, D. (2010). ZiFiT (Zinc Finger Targeter): an updated zinc finger engineering tool. Nucleic Acids Res *38*, W462-468.

S16. Reyon, D., Khayter, C., Regan, M.R., Joung, J.K., and Sander, J.D. (2012). Engineering designer transcription activator-like effector nucleases (TALENs) by REAL or REAL-Fast assembly. Curr Protoc Mol Biol *Chapter 12*, Unit 12.15.

S17. Sander, J.D., Cade, L., Khayter, C., Reyon, D., Peterson, R.T., Joung, J.K., and Yeh, J.R. (2011). Targeted gene disruption in somatic zebrafish cells using engineered TALENs. Nat Biotechnol *29*, 697-698.

S18. Bussmann, J., and Schulte-Merker, S. (2011). Rapid BAC selection for tol2-mediated transgenesis in zebrafish. Development *138*, 4327-4332.

S19. Balciunas, D., Wangensteen, K.J., Wilber, A., Bell, J., Geurts, A., Sivasubbu, S., Wang, X., Hackett, P.B., Largaespada, D.A., McIvor, R.S., et al. (2006). Harnessing a high cargo-capacity transposon for genetic applications in vertebrates. PLoS Genet *2*, e169.

S20. Schneider, C.A., Rasband, W.S., and Eliceiri, K.W. (2012). NIH Image to ImageJ: 25 years of image analysis. Nat Methods *9*, 671-675.

S21. Lowe, A.S., Nikolaou, N., Hunter, P.R., Thompson, I.D., and Meyer, M.P. (2013). A systems-based dissection of retinal inputs to the zebrafish tectum reveals different rules for different functional classes during development. J Neurosci *33*, 13946-13956.

S22. Peirce, J.W. (2008). Generating Stimuli for Neuroscience Using PsychoPy. Front Neuroinform *2*, 10.

S23. Chen, T.W., Wardill, T.J., Sun, Y., Pulver, S.R., Renninger, S.L., Baohan, A., Schreiter, E.R., Kerr, R.A., Orger, M.B., Jayaraman, V., et al. (2013). Ultrasensitive fluorescent proteins for imaging neuronal activity. Nature *499*, 295-300.

S24. Nikolaou, N., and Meyer, M.P. (2015). Lamination Speeds the Functional Development of Visual Circuits. Neuron *88*, 999-1013.

S25. Bulina, M.E., Chudakov, D.M., Britanova, O.V., Yanushevich, Y.G., Staroverov, D.B., Chepurnykh, T.V., Merzlyak, E.M., Shkrob, M.A., Lukyanov, S., and Lukyanov, K.A. (2006). A genetically encoded photosensitizer. Nat Biotechnol *24*, 95-99.

S26. Teh, C., Chudakov, D.M., Poon, K.L., Mamedov, I.Z., Sek, J.Y., Shidlovsky, K., Lukyanov, S., and Korzh, V. (2010). Optogenetic in vivo cell manipulation in KillerRed-expressing zebrafish transgenics. BMC Dev Biol *10*, 110.

S27. Bulina, M.E., Lukyanov, K.A., Britanova, O.V., Onichtchouk, D., Lukyanov, S., and Chudakov, D.M. (2006). Chromophore-assisted light inactivation (CALI) using the phototoxic fluorescent protein KillerRed. Nat Protoc *1*, 947-953.

S28. Nikolaev, A., Leung, K.M., Odermatt, B., and Lagnado, L. (2013). Synaptic mechanisms of adaptation and sensitization in the retina. Nat Neurosci *16*, 934-941.

S29. Hirata, H., Ogino, K., Yamada, K., Leacock, S., and Harvey, R.J. (2013). Defective escape behavior in DEAH-box RNA helicase mutants improved by restoring glycine receptor expression. J Neurosci *33*, 14638-14644.

S30. Inoue, D., and Wittbrodt, J. (2011). One for all--a highly efficient and versatile method for fluorescent immunostaining in fish embryos. PLoS One *6*, e19713.

S31. Niell, C.M., and Stryker, M.P. (2008). Highly selective receptive fields in mouse visual cortex. J Neurosci *28*, 7520-7536.

S32. Swindale, N.V. (1998). Orientation tuning curves: empirical description and estimation of parameters. Biol Cybern *78*, 45-56.

S33. Batschelet, E. (1981). Circular statistics in biology (London: Academic Press).

S34. Schindelin, J., Arganda-Carreras, I., Frise, E., Kaynig, V., Longair, M., Pietzsch, T., Preibisch, S., Rueden, C., Saalfeld, S., Schmid, B., et al. (2012). Fiji: an open-source platform for biological-image analysis. Nat Methods *9*, 676-682.
