# Supplementary figures and images for: Neural Mechanisms Generating Orientation Selectivity in the Retina
Source: Curr Biol. 2016 Jul 25;26(14):1802–15. doi: 10.1016/j.cub.2016.05.035 (PMC4963213; doi:10.1016/j.cub.2016.05.035)

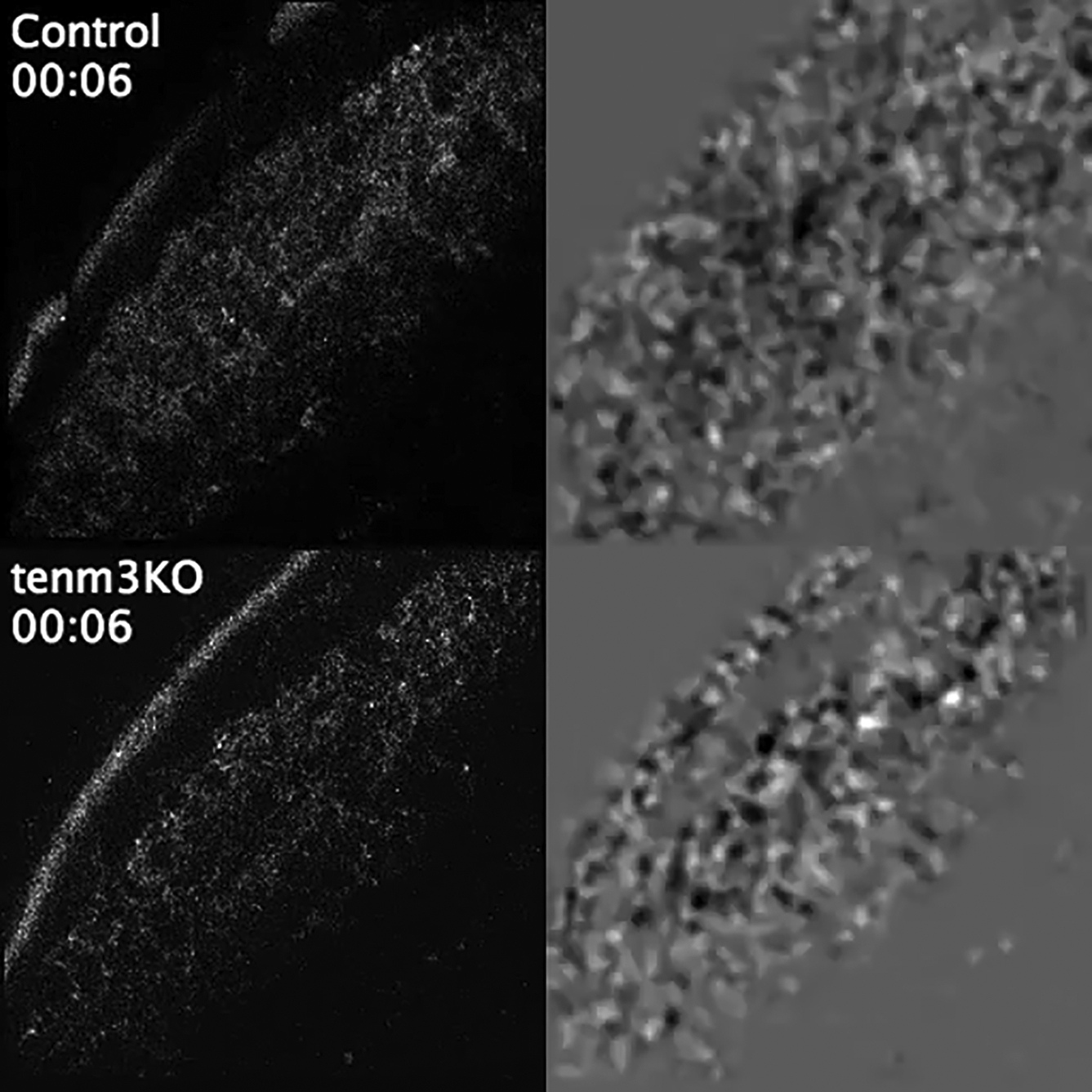

Supplement: Movie S1. RGC Visual Responses to Moving Bars in Control and tenm3KO 4-dpf Zebrafish Larvae, Related to Figure 1 — Time-lapse functional calcium imaging of representative control (top) and tenm3KO (bottom) 4 dpf Tg(isl2b:Gal4;UAS:SyGCaMP3) larvae. The movies encompass entire tuning experiments during which bars moving in 12 directions plus a blank screen null stimulus are presented to the right eye of immobilised zebrafish larvae. Unprocessed SyGCaMP3 responses are shown on the left, whereas ΔF/F0 calcium responses are reported on the right. The orientation of stimuli (°) is displayed when bars are projected onto the screen. Time is given in min:sec. The acquisition rate is 4.1 Hz, and the movie speed is approximately 5x. Anterior is up, posterior is down. [file mmc3.jpg]

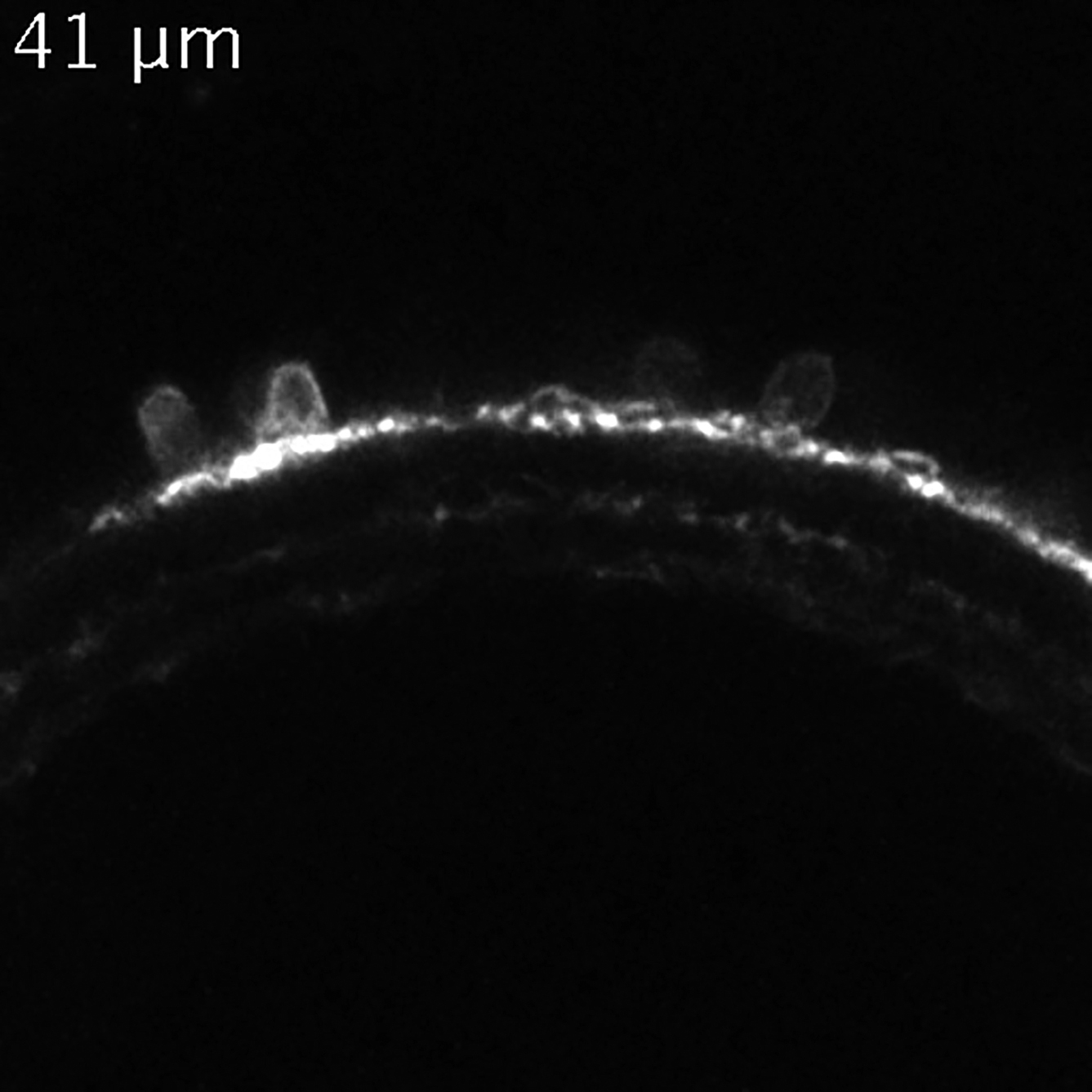

Supplement: Movie S2. Tenm3+ ACs in the Retina of a 4-dpf Zebrafish Larva, Related to Figure 2 — Z-stack showing the tenm3+ AC population in the retina of a 4 dpf Tg(tenm3:Gal4;UAS:tagRFPCAAX) larva. Relative depth is displayed in μm (46 μm total volume thickness with 1 μm Z resolution). Outer retina is up, inner retina is down. [file mmc4.jpg]

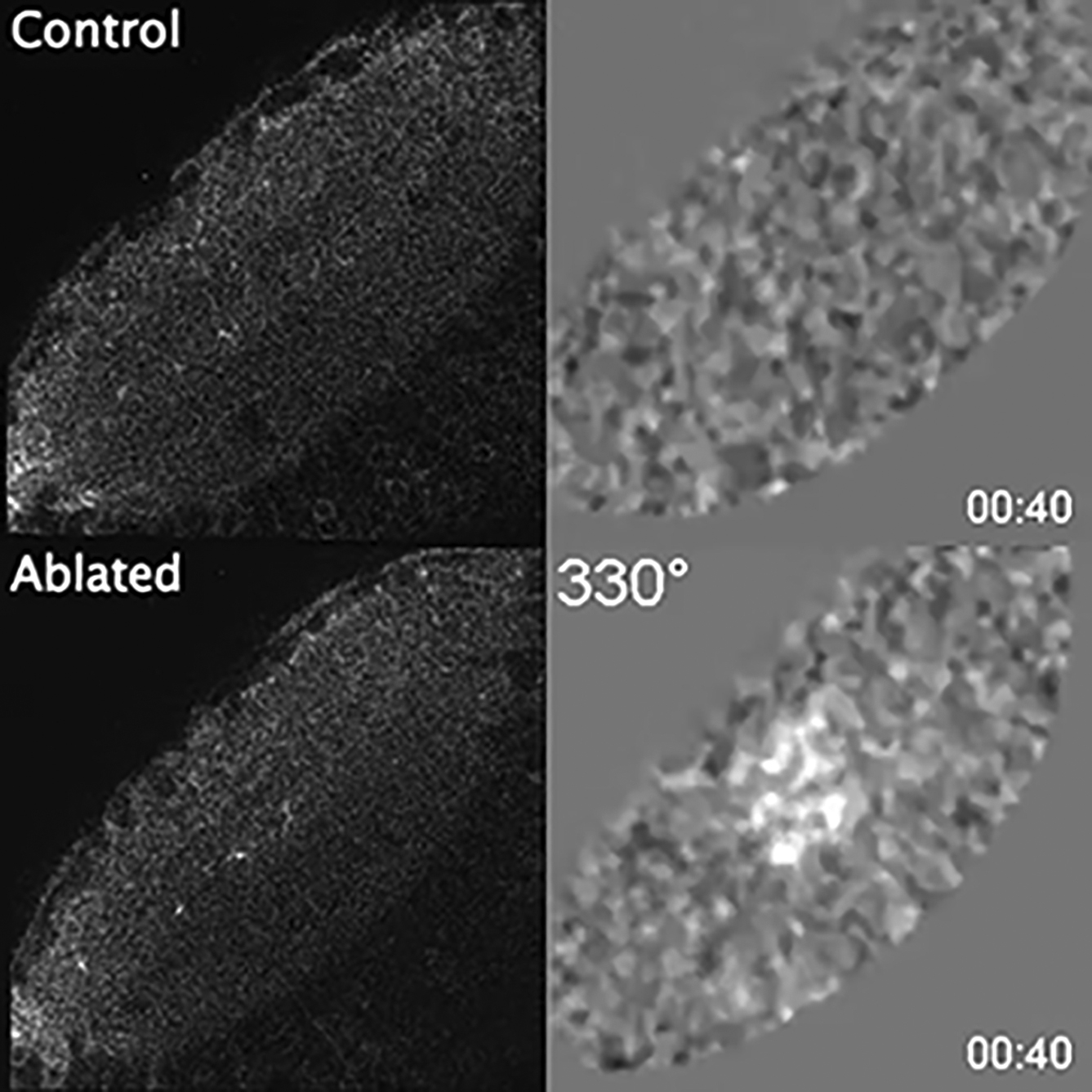

Supplement: Movie S3. RGC Visual Responses to Moving Bars in Control and tenm3+ AC-Ablated 4-dpf Zebrafish Larvae, Related to Figure 3 — Time-lapse functional calcium imaging of representative control (top) and tenm3+ AC-ablated (bottom) 4 dpf Tg(tenm3:Gal4;UAS:KillerRed;elavl3:GCaMP5G) larvae. The movies encompass entire tuning experiments during which bars moving in 12 directions plus a blank screen null stimulus are presented to the right eye of immobilised zebrafish larvae. Unprocessed GCaMP5G responses are shown on the left, whereas ΔF/F0 calcium responses are reported on the right. The orientation of stimuli (°) is displayed when bars are projected onto the screen. Local application of the glutamate receptor antagonists APV and NBQX (100 μM and 20 μM, respectively) in the optic tectum effectively blocks tectal cell calcium responses, therefore isolating RGC axonal responses. Time is given in min:sec. The acquisition rate is 4.1 Hz, and the movie speed is approximately 5x. Anterior is up, posterior is down. [file mmc5.jpg]

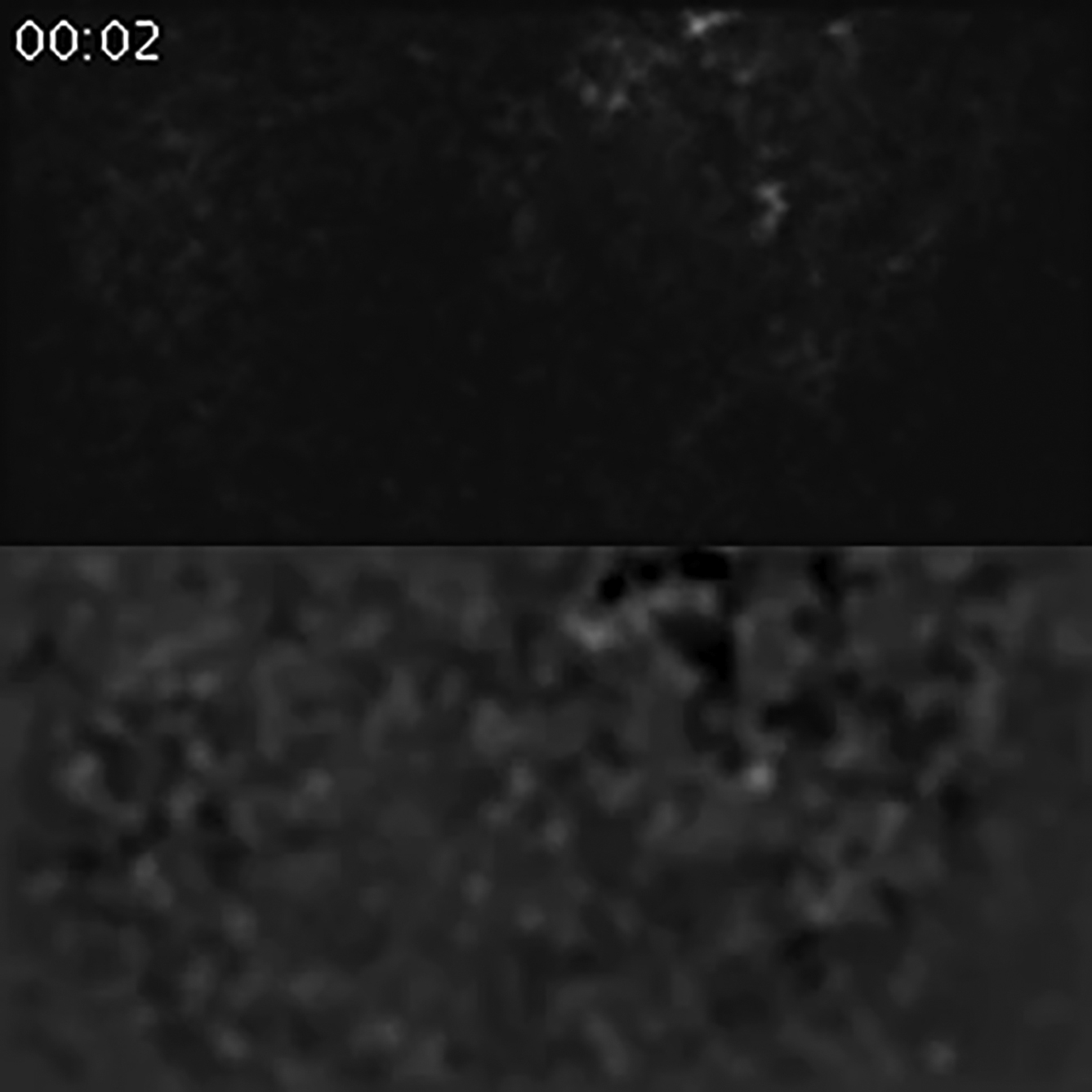

Supplement: Movie S4. Visual Responses to Moving Gratings of Tenm3+ AC Synaptic Terminals in a 4-dpf Zebrafish Larva, Related to Figure 6 — Time-lapse two-photon calcium imaging of a representative 4 dpf Tg(tenm3:Gal4;UAS:SyGCaMP3) larva. The movie encompasses an entire tuning experiment during which gratings moving in 12 directions plus a null stimulus are presented to one eye of the immobilised zebrafish larva. SyGCaMP3 fluorescence responses are shown at the top, whereas ΔF/F0 calcium responses are reported at the bottom. The orientation of stimuli (°) is reported when gratings are displayed on the LCD screen. Time is given in min:sec. The acquisition rate is 7.8 Hz, and the movie speed is approximately 5x. [file mmc6.jpg]

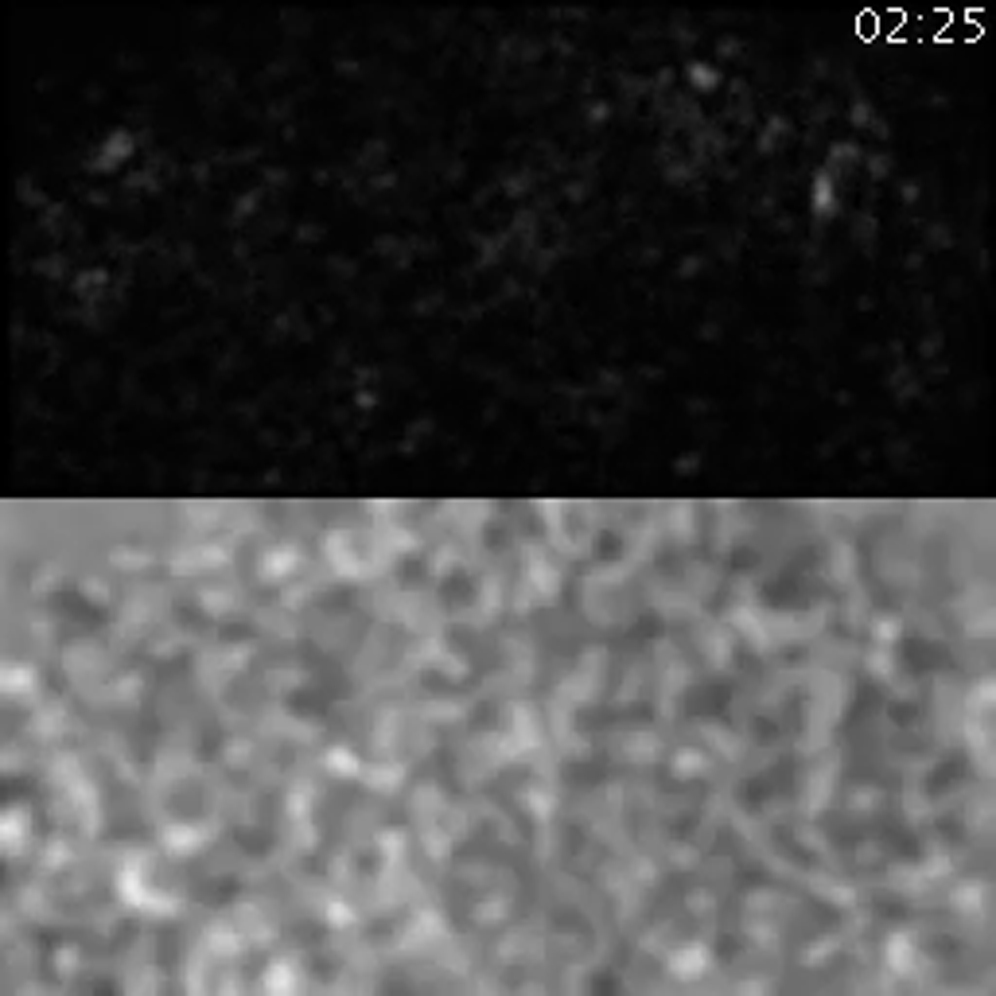

Supplement: Movie S5. Visual Responses to Moving Gratings of BC Ribbon Synapses in a 4-dpf Zebrafish Larva, Related to Figure 6 — Time-lapse two-photon calcium imaging of a representative 4 dpf Tg(-1.8ctbp2:SyGCaMP6) larva. The movie encompasses an entire tuning experiment during which gratings moving in 12 directions plus a null stimulus are presented to one eye of the immobilised zebrafish larva. SyGCaMP6 fluorescence responses are shown at the top, whereas ΔF/F0 calcium responses are reported at the bottom. The orientation of stimuli (°) is reported when gratings are displayed on the LCD screen. Time is given in min:sec. The acquisition rate is 7.8 Hz, and the movie speed is approximately 5x. [file mmc7.jpg]
