# Supplementary material for: Neural Mechanisms Generating Orientation Selectivity in the Retina
Source: Curr Biol. 2016 Jul 25;26(14):1802–15. doi: 10.1016/j.cub.2016.05.035 (PMC4963213; doi:10.1016/j.cub.2016.05.035)
Supplement: Document S2. Article plus Supplemental Information [file mmc8.pdf]

Article

Current Biology

Neural Mechanisms Generating Orientation Selectivity in the Retina

Graphical Abstract

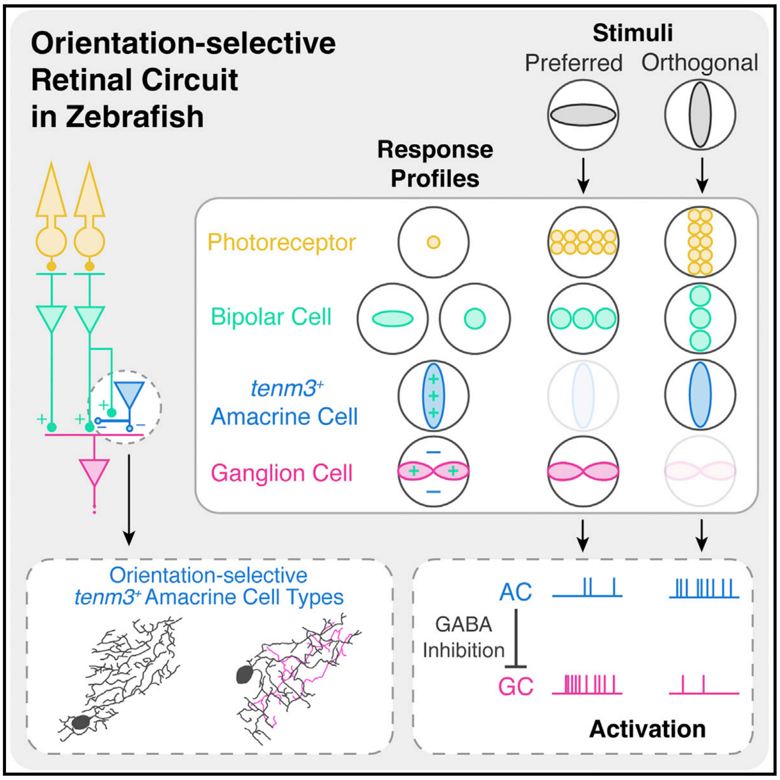

Highlights

- We identify Tenm3+ ACs with elongated dendritic arbors showing orientation tuning
- Tenm3+ AC GABAergic inhibition is crucial for orientation-selective RGC tuning
- Orientation tuning is present also among some bipolar cell presynaptic terminals
- We propose a model of how orientation selectivity is generated in ganglion cells

Authors

Paride Antinucci, Oniz Suleyman, Clinton Monfries, Robert Hindges

Correspondence

robert.hindges@kcl.ac.uk

In Brief

The neural mechanisms underlying orientation selectivity in the vertebrate retina are still poorly understood. Antinucci et al. identify a class of orientation-tuned amacrine cells characterized by elongated dendritic arbors that are essential for generating orientation selectivity in retinal ganglion cells.

Antinucci et al., 2016, Current Biology *26*, 1802–1815
July 25, 2016 © 2016 The Author(s). Published by Elsevier Ltd.
http://dx.doi.org/10.1016/j.cub.2016.05.035

Current Biology
Article

Neural Mechanisms Generating Orientation Selectivity in the Retina

Paride Antinucci,[1] Oniz Suleyman,[1] Clinton Monfries,[1] and Robert Hindges[1,*]
[1]MRC Centre for Developmental Neurobiology, King's College London, Guy's Campus, London SE1 1UL, UK
*Correspondence: robert.hindges@kcl.ac.uk
http://dx.doi.org/10.1016/j.cub.2016.05.035

SUMMARY

The orientation of visual stimuli is a salient feature of visual scenes. In vertebrates, the first neural processing steps generating orientation selectivity take place in the retina. Here, we dissect an orientation-selective circuit in the larval zebrafish retina and describe its underlying synaptic, cellular, and molecular mechanisms. We genetically identify a class of amacrine cells (ACs) with elongated dendritic arbors that show orientation tuning. Both selective optogenetic ablation of ACs marked by the cell-adhesion molecule Teneurin-3 (Tenm3) and pharmacological interference with their function demonstrate that these cells are critical components for orientation selectivity in retinal ganglion cells (RGCs) by being a source of tuned GABAergic inhibition. Moreover, our morphological analyses reveal that Tenm3$^+$ ACs and orientation-selective RGCs co-stratify their dendrites in the inner plexiform layer, and that Tenm3$^+$ ACs require Tenm3 to acquire their correct dendritic stratification. Finally, we show that orientation tuning is present also among bipolar cell presynaptic terminals. Our results define a neural circuit underlying orientation selectivity in the vertebrate retina and characterize cellular and molecular requirements for its assembly.

INTRODUCTION

The detection of oriented visual stimuli is a key neural computation performed by visual systems of many animals. Neurons performing this task are known as orientation selective (OS) since they respond preferentially to elongated stimuli oriented along a specific axis in the visual field but respond weakly to stimuli oriented orthogonally to their preferred axis. Orientation selectivity was first discovered in cat primary visual cortex by Hubel and Wiesel over 50 years ago [1]. Since then, numerous studies described OS neurons in visual systems of vertebrates and invertebrates, including primates [2], rodents [3], fish [4], and insects [5]. Work in several vertebrate species identified OS cells in regions upstream of primary visual cortex, like the lateral geniculate nucleus [6–8] and the retina [9–12], suggesting that the first steps in the processing of oriented stimuli take place early along the vertebrate visual pathway. In the retina, orientation selectivity is present among retinal ganglion cells (RGCs) [10, 13], the sole retinal output neurons, and amacrine cells (ACs) [13, 14], a class of inhibitory neurons that modulate and shape RGC responses. However, it is presently unclear how orientation selectivity emerges in these cells and whether they form a distinct retinal circuit, partially due to the lack of specific molecular markers allowing targeted labeling and manipulations.

The vertebrate retina consists of more than 70 neuron types [15]. Its primary function is to detect light stimuli, convert them into electrochemical signals, and, subsequently, send the processed information to higher visual nuclei through parallel feature-specific neural pathways. Most of the information processing takes place in a layered neuropil structure called the inner plexiform layer (IPL) [16]. Essential neural substrates underlying the computations performed in the IPL are the specific and stereotypic synaptic connections between three classes of neurons, namely, bipolar cells (BCs), ACs, and RGCs (Figure 1A). Recent developmental studies have shown that cell-adhesion molecules selectively expressed in specific retinal cell types mediate the matching between defined pre- and postsynaptic partners to establish this complex wiring pattern [18–20]. While several cell types and molecules crucial for establishing direction selectivity in the retina have been identified [20, 21], the equivalents for orientation selectivity are largely unknown to date.

Teneurins are a family of transmembrane cell-adhesion proteins that play a synaptic matching role in the *Drosophila* olfactory system [22] and neuromuscular junction [23]. In vertebrates, teneurins are highly expressed in several interconnected regions of the brain, including the visual system [24, 25]. In vitro and in vivo data suggest that *trans*-synaptic interactions are possible both homophilically through their five NHL domains [22, 26], and heterophilically with the cell-adhesion G-protein-coupled receptors latrophilins [27, 28]. We previously showed that *teneurin-3* (*tenm3*) is expressed in zebrafish ACs and RGCs during a period of intense synaptic formation (Figure 1B) and that, when *tenm3* is knocked down, RGC dendrites fail to correctly stratify in the IPL [17]. We also reported a functional link between *tenm3* and RGC orientation selectivity.

Here, using *tenm3* as a marker, we identify crucial cellular players and mechanisms generating orientation selectivity in the larval zebrafish retina. First, we reveal that *tenm3*-expressing (*tenm3*$^+$) ACs co-stratify their neurites with orientation-selective RGC (OSGC) dendrites and that, upon *tenm3* knockout, they fail to correctly stratify their neurites in the IPL. Second, we show evidence suggesting that *tenm3*$^+$ ACs generate OSGC tuning by providing γ-aminobutyric acid (GABA) feedforward inhibitory input. Third, we identify and characterize orientation-tuned *tenm3*$^+$ AC types with elongated dendritic arbors. Fourth, we find

 © 2016 The Author(s). Published by Elsevier Ltd.
This is an open access article under the CC BY license (http://creativecommons.org/licenses/by/4.0/).

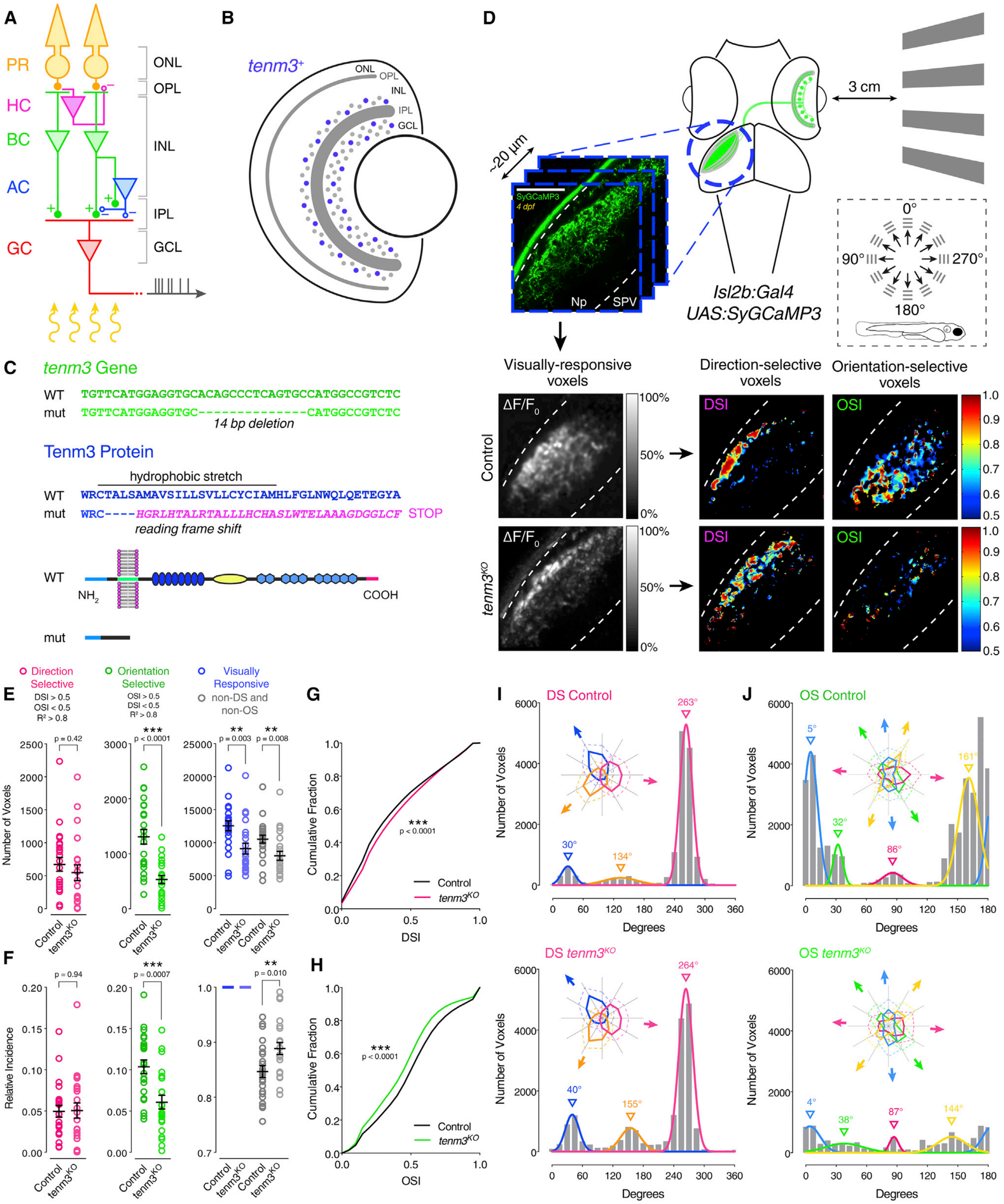

Figure 1. Tenm3 Is Required for RGC Orientation Selectivity

(A) Basic neural circuit structure of the vertebrate retina. Cell classes are represented in colors, whereas layers are shown in black. Excitatory synapses are indicated by "+" (filled circles), inhibitory synapses are labeled with "–" (empty circles). PR, photoreceptor; HC, horizontal cell; BC, bipolar cell; AC, amacrine cell; RGC, retinal ganglion cell; ONL, outer nuclear layer; INL, inner nuclear layer; GCL, ganglion cell layer; OPL, outer plexiform layer; IPL, inner plexiform layer.

(legend continued on next page)

that a fraction of BC presynaptic terminals show orientation tuning. Finally, we present a circuit model describing how OSGCs acquire their orientation selectivity by integrating tuned *tenm3*$^+$ ACs and BC inputs.

RESULTS

Generation of a Tenm3^{KO} Tool to Study Retinal Orientation Selectivity

Previous work using transient gene knockdown methods suggested that Tenm3 is involved in the development of orientation selectivity in zebrafish RGCs [17]. To confirm this role and, subsequently, use Tenm3 as a marker to identify cells generating RGC orientation selectivity, we generated a zebrafish *tenm3* knockout mutant (*tenm3*^{KO}) using Transcription Activator-Like Effector Nuclease (TALEN)-based genome editing (see Supplemental Experimental Procedures). In *tenm3*^{KO} mutants, a 14-bp deletion in the exon encoding the transmembrane domain of Tenm3 leads to a reading frameshift and, subsequently, to a premature stop codon causing the loss of the entire extracellular domain (Figures 1C and S1A–S1F). We then examined the retinal functional output of *tenm3*^{KO} mutants as previously described [4, 17]. Briefly, drifting bars moving in 12 different directions were presented to awake immobilized zebrafish larvae through a projection screen (Figure 1D). Using the RGC-specific transgenic line *Tg(isl2b:Gal4;UAS:SyGCaMP3)*, population visual responses were simultaneously recorded through calcium imaging of RGC axon terminals in the contralateral optic tectum (Movie S1). Voxel-wise analysis was then used to isolate visually responsive voxels and identify direction-selective (DS) and OS responses (Figures 1D and S1G) [29]. We found that 4 days post-fertilization (dpf) *tenm3*^{KO} mutants have a large decrease in both the number of OS voxels (Figure 1E) and the proportion of OSGC output relative to the whole population of responsive voxels (Figure 1F). As a consequence, the relative proportion of "non-tuned" (non-DS and non-OS) RGC output was increased in *tenm3*^{KO} mutants (Figure 1F). This impairment in the OSGC population is consistent with the lower degree of orientation selectivity, quantified by the orientation selectivity index (OSI), across visually responsive voxels in *tenm3*^{KO} mutants (Figure 1H). By contrast, the direction-selective RGC (DSGC) population of responses did not show any impairment in *tenm3*^{KO} mutants (Figures 1E–1G), suggesting that Tenm3 is not involved in the assembly of DS circuits. Equivalent results were obtained in 7-dpf *tenm3*^{KO} mutants (Figures S1H–S1K), indicating that the development of RGC orientation selectivity is not simply delayed. A modest but significant decrease in the number of visually responsive voxels was observed in *tenm3*^{KO} mutants at 4 dpf (Figure 1E), but not at 7 dpf (Figure S1H).

We next explored to what extent the subtypes of DSGCs and OSGCs previously described in zebrafish [29] were affected by Tenm3 loss of function. Different subpopulations of DSGC and OSGC responses were identified by fitting Gaussian distributions to the grouped population data of preferred angles [4, 29]. As expected, population sizes and relative proportions of the three subtypes of DSGCs were not altered in *tenm3*^{KO} mutants (Figures 1I and S1L), reinforcing the view that RGC direction selectivity develops through Tenm3-independent mechanisms. Interestingly, the decrease in OS responses in *tenm3*^{KO} mutants was not equally represented among the four OSGC subtypes, with the small OSGC subpopulation tuned to vertical bars moving along the horizontal axis being the least affected (magenta, Figures 1J and S1M). Overall, these results confirm and further elucidate the crucial role played by Tenm3 in generating RGC orientation selectivity during development [17]. Additionally, they provide a genetic access point to reveal the individual circuit components and mechanisms underlying retinal orientation selectivity.

Neurite Stratification Pattern of Tenm3$^+$ ACs and OSGCs

Since *tenm3* is expressed not only in RGCs, but also in ACs [17], we asked whether the functional phenotype observed in *tenm3*^{KO} OSGCs results from structural defects in the presynaptic AC population. We thus generated a zebrafish bacterial artificial chromosome (BAC) transgenic line, *Tg(tenm3:Gal4)*, where Gal4FF is under transcriptional control of regulatory elements upstream and downstream of the *tenm3* start codon (Figure 2A) (see Supplemental Experimental Procedures). In this BAC line, Gal4 is expressed in brain regions where *tenm3* is endogenously expressed, including the retina and optic tectum (Figure S2) [17]. In the retina, the *Tg(tenm3:Gal4;UAS:tagRFP-CAAX)* line labels a subset of ACs (hereafter referred to as *tenm3*$^+$ ACs) but fails to drive expression in RGCs (Figures 2B and S2A–S2C; Movie S2), possibly due to a lack of RGC-specific regulatory elements

(B) Schematic showing *tenm3* mRNA expression in the retina of zebrafish larvae. Blue circles indicate *tenm3*$^+$ ACs and RGCs. Adapted from Antinucci et al. [17].

(C) TALEN-mediated *tenm3* gene knockout (top) and consequent structural changes in the Tenm3 protein (bottom).

(D) Functional calcium imaging of RGC axon terminals expressing SyGCaMP3 (green) in 4-dpf *Tg(isl2b:Gal4;UAS:SyGCaMP3)* larvae. Distance of right eye from projection screen is 3 cm. Recordings are performed from two to four Z-planes (approximately 20 μm total volume thickness) in the contralateral optic tectum. Dashed box shows the angles of moving bars relative to zebrafish larva orientation. Mean $\Delta F/F_0$ images of calcium recordings in control and *tenm3*^{KO} larvae followed by mapping of DS and OS voxels are displayed. Np, neuropil; SPV, stratum periventriculare; DSI, direction selectivity index; OSI, orientation selectivity index. Scale bar, 40 μm.

(E and F) Average number (E) and relative frequency (F) of DS, OS, visually responsive, and non-DS/non-OS voxels per Z-plane in control (n = 23 larvae) and *tenm3*^{KO} (n = 22 larvae) 4-dpf larvae. Criteria used to identify DS and OS voxels are reported at the top. Error bars, ±SEM. $^{**}p < 0.01$, $^{***}p < 0.001$, unpaired two-tailed Student's t test.

(G and H) Cumulative distributions of DSI values ($R^2 > 0$) across voxels with OSI < 0.5 (G) and OSI values ($R^2 > 0$) across voxels with DSI < 0.5 (H) in control and *tenm3*^{KO} larvae. $^{***}p < 0.001$, two-sample Kolmogorov-Smirnov test.

(I and J) Cumulative histograms summarizing the incidence of preferred angles for identified DS (I) and OS voxels (J) in control (n = 23; top) and *tenm3*^{KO} (n = 22; bottom) 4-dpf larvae. Overlaid curves are the fitted Gaussian distributions for each DS or OS subtype. Polar plots illustrate the mean (+1 SD) normalized response profiles for each DS or OS subtype.

See also Figure S1, Table S1, and Movie S1.

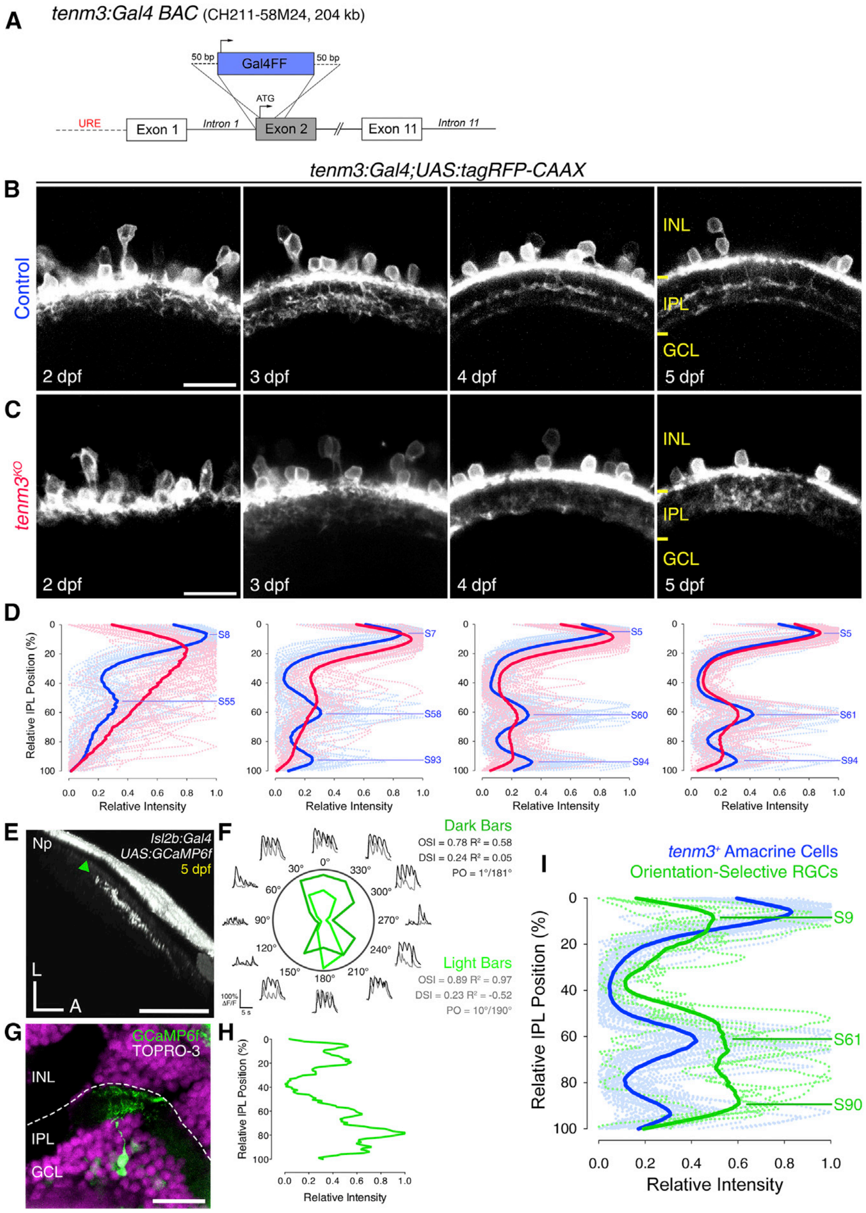

Figure 2. IPL Stratification Pattern of Tenm3⁺ ACs and OSGCs

(A) Schematic of the bacterial artificial chromosome (BAC) DNA construct used to transgenically express Gal4FF in *tenm3*⁺ cells. URE, upstream regulatory elements.

(B and C) Inner plexiform layer (IPL) stratification pattern of *tenm3*⁺ AC neurites in control (B) and *tenm3*^{KO} (C) *Tg(tenm3:Gal4;UAS:tagRFP-CAAX)* larvae from 2 to 5 dpf. INL, inner nuclear layer; GCL, ganglion cell layer. Scale bars, 20 μm.

(D) IPL fluorescence intensity profiles of *tenm3*⁺ AC neurites in control (blue; n = 13 larvae) and *tenm3*^{KO} larvae (red; n = 13 larvae) from 2 to 5 dpf. Thin traces represent individual IPL profiles, whereas thick traces indicate average IPL profiles. 0% corresponds to the INL/IPL boundary, whereas 100% corresponds to the IPL/GCL boundary. Fluorescence peaks indicating IPL strata in control larvae are labeled with the letter "S" followed by their relative IPL position.

(E and F) Visual responses to moving bars (F) recorded through calcium imaging of an individual orientation-selective RGC (OSGC) axon terminal expressing GCaMP6f (E, green arrowhead) in the optic tectum of a 5-dpf *UAS:GCaMP6f*-injected *Tg(isl2b:Gal4)* larva. Polar plots show the integral responses to moving dark and light bars (F; dark and light green, respectively). Black and gray traces represent the $\Delta F/F_0$ calcium responses to moving dark and light bars, respectively. Np, neuropil; L, lateral; A, anterior; PO, preferred orientation. Scale bar, 40 μm.

(G and H) Immunostaining for GCaMP6f (green) showing the dendritic morphology (G) of the functionally identified OSGC in (E) and the corresponding normalized IPL fluorescence intensity profile (H). Cell bodies are labeled with the nuclear stain TO-PRO-3 (magenta). Scale bar, 20 μm.

(I) IPL fluorescence intensity profiles of OSGCs (green; n = 5 cells) and *tenm3*⁺ AC neurites (blue; n = 13 larvae) at 5 dpf. 12.8% of functionally imaged RGCs were OS (five out of 39 cells in 39 larvae). Thin traces represent individual IPL profiles, whereas thick traces indicate average IPL profiles. Fluorescence peaks indicating IPL strata formed by OSGC dendrites are labeled with the letter "S" followed by their relative IPL position.

See also Figures S2 and S3 and Movie S2.

in the BAC construct used for transgenesis. We therefore used this line to selectively visualize the morphological development of *tenm3*⁺ ACs from 2 to 5 dpf. During this period, *tenm3* is highly expressed in the retina [17], and, at 3 dpf, RGCs start to show orientation selectivity [29]. Interestingly, *tenm3*⁺ ACs stratify their neurites in three precise IPL strata located at 5%, 61%, and 94% depth (0% corresponds to the inner nuclear layer (INL)/IPL border, 100% to the IPL/GCL border), which were named S5, S61, and S94, respectively (Figures 2B and 2D). This tri-laminar IPL stratification pattern is visible at 3 dpf and gradually refines over the following 2 developmental days. In *tenm3*^{KO} mutants, by contrast, *tenm3*⁺ AC neurites do not stratify correctly in the IPL (Figure 2C). This is particularly striking at 3 dpf when they fail to target the two innermost IPL strata and instead stratify diffusely across the IPL (Figure 2D).

An indication of potential synaptic connections between *tenm3*⁺ ACs and OSGCs would be their dendritic co-stratification in the IPL. Currently, no transgenic line exists to selectively label OSGCs and directly detect dendritic co-stratification with *tenm3*⁺ ACs. Therefore, we sparsely expressed GCaMP6f [30] in individual RGCs and, after functionally identifying OSGCs, we performed post hoc immunostaining to analyze their IPL stratification pattern (Figures 2E–2G). Then, we averaged fluorescence intensity profiles of dendritic stratification from multiple OSGCs (Figures 2H and S3) and overlaid the resulting mean profile (green, Figure 2I) to the IPL stratification profile of *tenm3*⁺ ACs (blue). We found that, as a population, OSGCs stratify their dendrites in three strata located at 9%, 61%, and 90% IPL depth and indeed show a high degree of overlap with *tenm3*⁺ AC neurites (Figure 2I). These results, together with the functional impairment

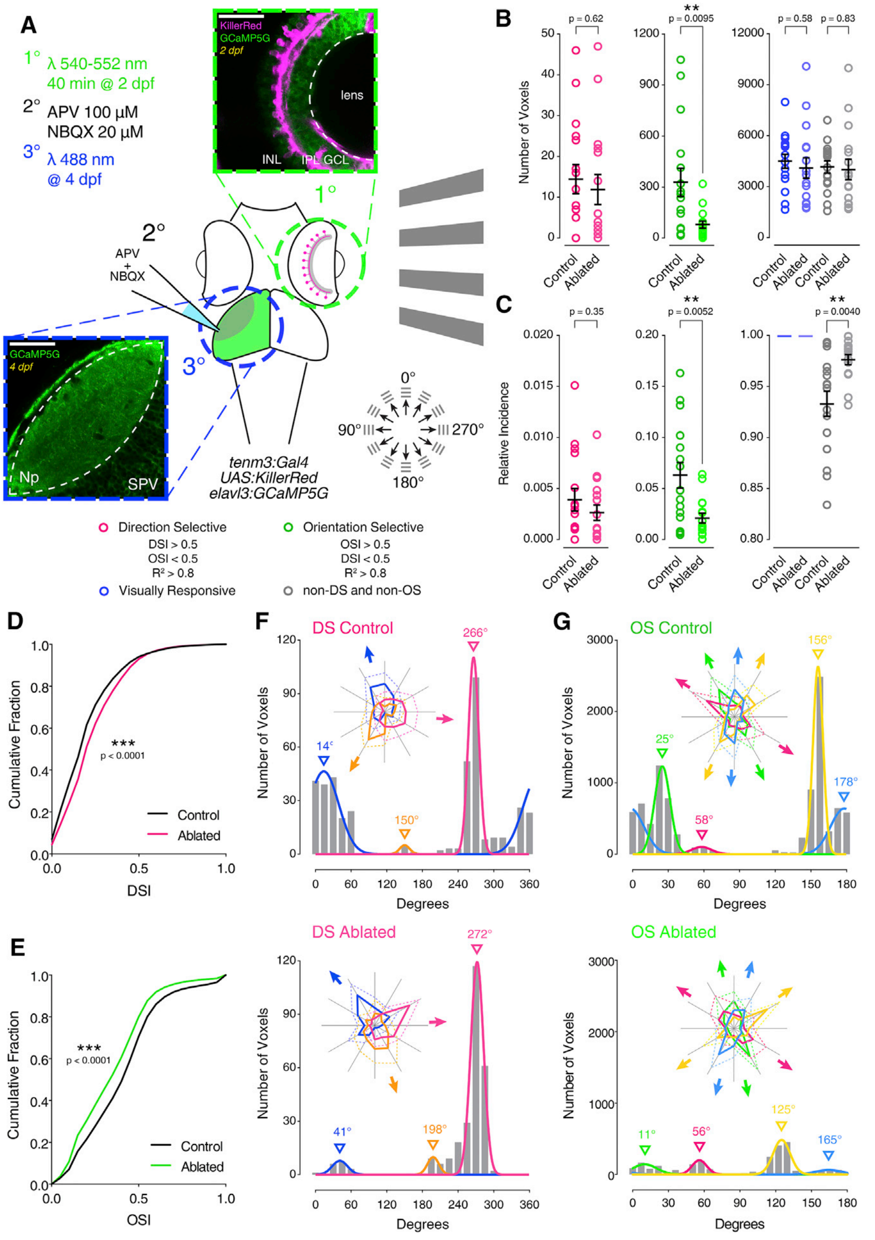

Figure 3. Tenm3⁺ ACs Generate Orientation Tuning in RGCs

(A) Summary of the experimental procedures used to record visual responses from larvae where *tenm3*⁺ ACs were optogenetically ablated. At 2 dpf, the eyes of *Tg(tenm3:Gal4;UAS:KillerRed; elavl3:GCaMP5G)* larvae, where KillerRed is selectively expressed in *tenm3*⁺ ACs only (magenta), are illuminated with green light (540–552 nm) for 40 min. Then, at 4 dpf, visual responses to moving bars are recorded through calcium imaging of RGC axon terminals (expressing GCaMP5G; green) in the optic tectum contralateral to the illuminated eye. Local application of the glutamate receptor antagonists APV and NBQX (100 and 20 μM, respectively) is used to isolate RGC axonal calcium responses from tectal cell dendritic responses. INL, inner nuclear layer; GCL, ganglion cell layer; IPL, inner plexiform layer; Np, neuropil; SPV, stratum periventriculare. Scale bars, 40 μm.

(B and C) Average number (B) and relative frequency (C) of DS, OS, visually responsive, and non-DS/non-OS voxels per Z-plane in control (n = 16 larvae) and *tenm3*⁺ AC ablated (n = 16 larvae) 4-dpf larvae. Error bars, ±SEM. **p < 0.01, unpaired two-tailed Student's t test.

(D and E) Cumulative distributions of DSI values ($R^2 > 0$) across voxels with OSI < 0.5 (D) and OSI values ($R^2 > 0$) across voxels with DSI < 0.5 (E) in control and *tenm3*⁺ AC ablated larvae. ***p < 0.001, two-sample Kolmogorov-Smirnov test.

(F and G) Cumulative histograms summarizing the incidence of preferred angles for identified DS (F) and OS voxels (G) in control (n = 16; top) and *tenm3*⁺ AC ablated (n = 16; bottom) 4-dpf larvae. Overlaid curves are the fitted Gaussian distributions for each DS or OS subtype. Polar plots illustrate the mean (+1 SD) normalized response profiles for each DS or OS subtype.

See also Figure S4, Table S1, and Movie S3.

in RGC orientation selectivity (Figure 1) and previously reported defects in RGC dendritic IPL stratification following Tenm3 loss of function [17] strongly suggest that *tenm3*⁺ ACs and OSGCs are part of the same circuit and that Tenm3 is involved in the morphological and functional development of these two neural populations.

Tenm3⁺ ACs Generate OSGC Tuning through GABAergic Inhibition

To investigate whether *tenm3*⁺ ACs play a role in the emergence of RGC orientation selectivity, we took advantage of the *Tg(tenm3:Gal4)* line to selectively ablate these cells and assess the functional consequences in RGCs. In the *Tg(tenm3:Gal4; UAS:KillerRed;elavl3:GCaMP5G)* line, the genetically encoded photosensitizer KillerRed [31] is expressed in *tenm3*⁺ ACs, whereas GCaMP5G is expressed pan-neuronally (Figure 3A) [32]. At 2 dpf, we optogenetically ablated *tenm3*⁺ ACs by illuminating the retina with intense green light (540–552 nm) for 40 min (Figure S4; see Supplemental Experimental Procedures). Subsequently, at 4 dpf, we recorded RGC visual responses to moving bars as described above (Movie S3). To isolate RGC axonal calcium responses from tectal cell dendritic responses, we locally applied the glutamate receptor antagonists APV and NBQX (100 and 20 μM, respectively) in the tectum [33]. Unlike KillerRed-positive larvae, control larvae subjected to the same procedures did not exhibit retinal cell death (Figure S4). Animals subjected to *tenm3*⁺ AC ablation showed a dramatic impairment in RGC orientation selectivity but no detrimental change in DSGC responses (Figures 3B–3E). The magnitude of the decrease in number of OS voxels, relative proportion of OSGC output and overall degree of RGC orientation selectivity was analogous to what we observed in *tenm3*^{KO} mutants. Moreover, the OSGC subpopulation tuned to vertical stimuli was the least affected by *tenm3*⁺ AC

ablation (magenta, Figure 3G), matching our Tenm3 KO results. Compared to data acquired using the *Tg(isl2b:Gal4;UAS:SyGCaMP3)* line (Figures 1I and 1J), we observed differences in the relative proportions of DSGC and OSGC subtypes as well as in their preferred directions or orientations both in control and *tenm3*$^+$ AC ablated groups (Figures 3F and 3G), likely resulting from the use of a different transgenic line or the pharmacological treatment used to isolate RGC responses.

The results obtained by ablating *tenm3*$^+$ ACs strongly support the idea that the output of *tenm3*$^+$ ACs is crucial in generating RGC orientation selectivity. We therefore aimed to reveal the role played by *tenm3*$^+$ AC neurotransmission in performing this neural computation. Immunohistochemical analyses showed that most *tenm3*$^+$ ACs are GABAergic and express the calcium-binding protein Parvalbumin (Figures 4A, 4B, and 4F). *Tenm3*$^+$ ACs also comprise dopaminergic ACs, which constitute a very small fraction of the whole AC population in zebrafish (Figure 4C) [34]. Negligible or no co-localization was observed between *tenm3*$^+$ ACs and cholinergic or glycinergic ACs, respectively (Figures 4D–4F). Cholinergic ACs correspond to starburst ACs (SACs), which are key cellular players in generating RGC direction selectivity [35], consistent with the observation that neither Tenm3 KO nor *tenm3*$^+$ AC ablation affected DSGC tuning. We next tested the role of GABA-mediated inhibition in producing OSGC tuning by blocking $GABA_A$ receptors through picrotoxin (100 μM). RGC visual responses were recorded from the same *Tg(isl2b:Gal4;UAS:SyGCaMP3)* larvae before and after drug application. Notably, OSGCs were severely affected by GABA inhibition block, with a decrease in both OS responses and overall degree of RGC orientation selectivity comparable to the effects seen in *tenm3*^{KO} mutants and after *tenm3*$^+$ AC ablation (Figures 4G, 4H, and 4J). Similarly to the knockout and ablation experiments, the small OSGC subpopulation tuned to vertical bars was the least affected by the pharmacological block (magenta, Figure 4L). As expected, RGC direction selectivity was also negatively impacted (Figures 4G–4I and 4K), since directionally tuned GABAergic inhibitory input from SACs plays an essential role in most DSGCs [35]. Compared to the impairments in RGC direction and orientation selectivity caused by blocking $GABA_A$ receptors, the effects produced by blocking glycine receptors using strychnine (70 μM) were minimal (Figures S5A–S5F). Taken together, these results demonstrate that OSGCs require GABAergic inhibitory input, likely from *tenm3*$^+$ ACs, to acquire their orientation tuning.

Single-Cell Morphologies of Tenm3$^+$ AC Types

To explore a possible link between the morphology of *tenm3*$^+$ ACs and the function they play in the OS circuit, we sparsely labeled *tenm3*$^+$ ACs by injecting *UAS:eGFP-CAAX* DNA constructs into 1-4 cell-stage *Tg(tenm3:Gal4)* embryos. We identified seven types of *tenm3*$^+$ ACs characterized by distinct morphological properties (Figures 5A–5F). These types differ in terms of their observed frequency, IPL dendritic stratification, dendritic field area (Figures 5I and 5J), and, interestingly, dendritic field elongation, quantified by calculating the eccentricity of their dendritic fields (Figures 5G and 5H). The most frequent *tenm3*$^+$ AC type (type I, 43% of *tenm3*$^+$ ACs) is a narrow-field AC with a dendritic arbor mono-stratified in S5 (Figure 5A). Type II and III *tenm3*$^+$ ACs (19% and 16% of *tenm3*$^+$ ACs, respectively) are medium-field ACs characterized by highly elongated dendritic fields (Figures 5B, 5C, and 5H). Their dendritic arbors stratify differently in the IPL with type II *tenm3*$^+$ ACs having mono-stratified neurites in S5, and type III *tenm3*$^+$ ACs showing a bi-stratified dendritic arbor in S5 and S61. Type IV-ON and IV-OFF *tenm3*$^+$ ACs (each 8% of *tenm3*$^+$ ACs) are mono-stratified medium-field ACs characterized by circular dendritic fields of similar area but different IPL stratification pattern, with the ON type stratifying in the innermost stratum (S94) and the OFF type in the outermost stratum (S5; Figures 5D and 5E). Finally, type V and VI *tenm3*$^+$ ACs are the least frequent *tenm3*$^+$ AC types (each 3% of *tenm3*$^+$ ACs) and possess wide-field dendritic arbors. Type V has extensive, radially arranged neurites covering most of the retina (Figure 5F). Type VI corresponds to the dopaminergic interplexiform AC previously described in goldfish (Figure 4C) [36]. Importantly, k-means clustering based on IPL stratification, dendritic field area, and dendritic field elongation support the notion that the different *tenm3*$^+$ ACs identified here are indeed defined AC types (Figures 5L and 5M; type V and VI ACs were not included in the clustering). Moreover, several lines of evidence suggest that type I–IV *tenm3*$^+$ ACs are arranged in mosaics tiling the retina with a coverage factor close to 1: (1) their frequency scales quadratically as a function of their dendritic field area (Figure 5K); (2) their observed frequency does not differ significantly from the frequency estimated assuming a retinal coverage factor of 1 (Figure 5N). Interestingly, the high dendritic field elongation of type II and III *tenm3*$^+$ ACs (Figures 5B, 5C, and 5H) is a feature previously described also in rabbit orientation-sensitive ACs [13, 14]. This led us to hypothesize that type II and III *tenm3*$^+$ ACs could produce OS responses when stimulating the retina with elongated stimuli oriented along particular axes in the visual field and, consequently, constitute cellular elements underlying the emergence of retinal orientation selectivity.

Tenm3$^+$ ACs Show Orientation Tuning

To analyze tuning in the *tenm3*$^+$ AC population, we performed in vivo two-photon calcium imaging in the retinae of *Tg(tenm3:Gal4;UAS:SyGCaMP3)* larvae (Figure 6A; see Supplemental Experimental Procedures). We found that *tenm3*$^+$ ACs show stimulus-locked responses to moving square-wave gratings (Movie S4). Notably, analyses using different metrics of orientation selectivity (i.e., OSI and circular variance) and progressively higher tuning stringency levels revealed a large fraction of *tenm3*$^+$ ACs tuned to elongated stimuli (Figures S6A–S6D). The distribution of preferred stimulus orientations across *tenm3*$^+$ ACs indicated the presence of four subpopulations of OS responses tuned to gratings oriented along the cardinal (13°, 90°) and diagonal axes (40°, 145°; Figure 6B), similar to what we observed in OSGCs (Figure 1J). Compared to RGCs, however, *tenm3*$^+$ ACs exhibited a higher degree of orientation selectivity (Figures 6C–6E). If activation of *tenm3*$^+$ ACs along a particular axis in the visual field leads to inhibition of OSGC responses along that axis, one would expect that the relationship between their population distributions is inversely correlated. We therefore analyzed the frequency distribution of the four OS subpopulations in *tenm3*$^+$ ACs and RGCs (Figures 6I and 6J). We found that they are indeed anti-correlated (Figure 6K), suggesting that the OS inhibitory input provided by *tenm3*$^+$ ACs to OSGCs is

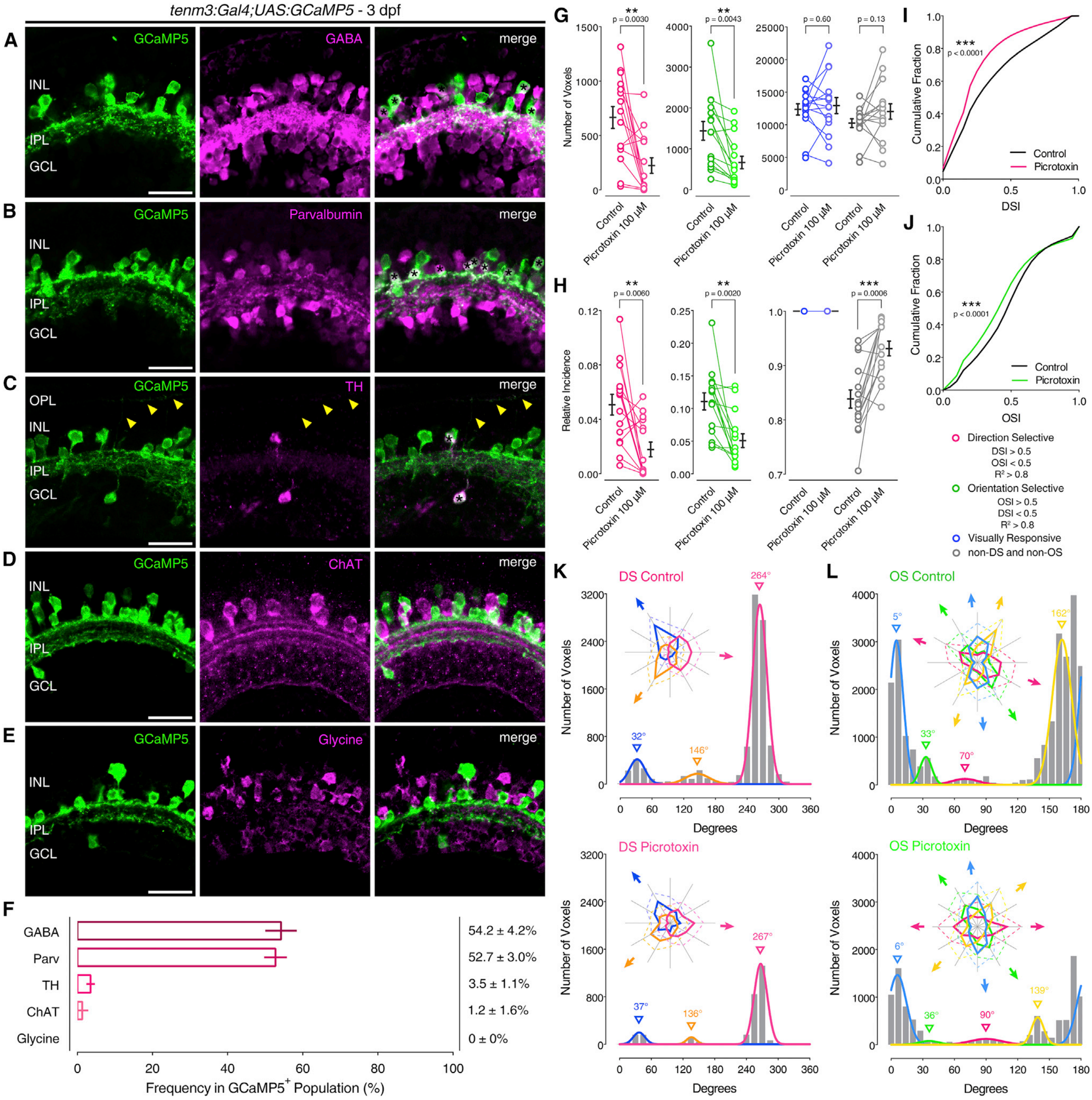

Figure 4. Role of Tenm3$^+$ AC GABAergic Inhibition in RGC Orientation Selectivity

(A–E) Immunostaining showing the expression of γ-aminobutyric acid (GABA; A), parvalbumin (B), tyrosine hydroxylase (TH; C), choline acetyltransferase (ChAT; D), and glycine (E) (all in magenta) in 3-dpf *Tg(tenm3:Gal4;UAS:GCaMP5)* larvae, where *tenm3*$^+$ ACs are labeled with GCaMP5 (green). Yellow arrowheads indicate neurites of TH$^+$ interplexiform ACs (C). INL, inner nuclear layer; GCL, ganglion cell layer; OPL, outer plexiform layer; IPL, inner plexiform layer. Scale bars, 20 μm.

(F) Percentage of GCaMP5$^+$ cells co-localizing with antigen$^+$ cells (mean ± SD). GABA, n = 13 retinae; Parvalbumin, n = 10 retinae; TH, n = 9 retinae; ChAT, n = 10 retinae; glycine, n = 5 retinae.

(G and H) Average number (G) and relative frequency (H) of DS, OS, visually responsive, and non-DS/non-OS voxels per Z-plane in 4-dpf *Tg(isl2b:Gal4;UAS:SyGCaMP3)* larvae (n = 15 larvae) before (control) and after (picrotoxin) the application of picrotoxin (100 μM). Error bars, ±SEM. **$p < 0.01$, ***$p < 0.001$, paired two-tailed Student's t test.

(I and J) Cumulative distributions of DSI values ($R^2 > 0$) across voxels with OSI < 0.5 (I) and OSI values ($R^2 > 0$) across voxels with DSI < 0.5 (J) before (control) and after (picrotoxin) the application of picrotoxin (100 μM). ***$p < 0.001$, two-sample Kolmogorov-Smirnov test.

(K and L) Cumulative histograms summarizing the incidence of preferred angles for identified DS (K) and OS voxels (L) in 4-dpf larvae (n = 15 larvae) before (control) and after (picrotoxin) the application of picrotoxin (100 μM). Overlaid curves are the fitted Gaussian distributions for each DS or OS subtype. Polar plots illustrate the mean (+1 SD) normalized response profiles for each DS or OS subtype.

See also Figure S5 and Table S1.

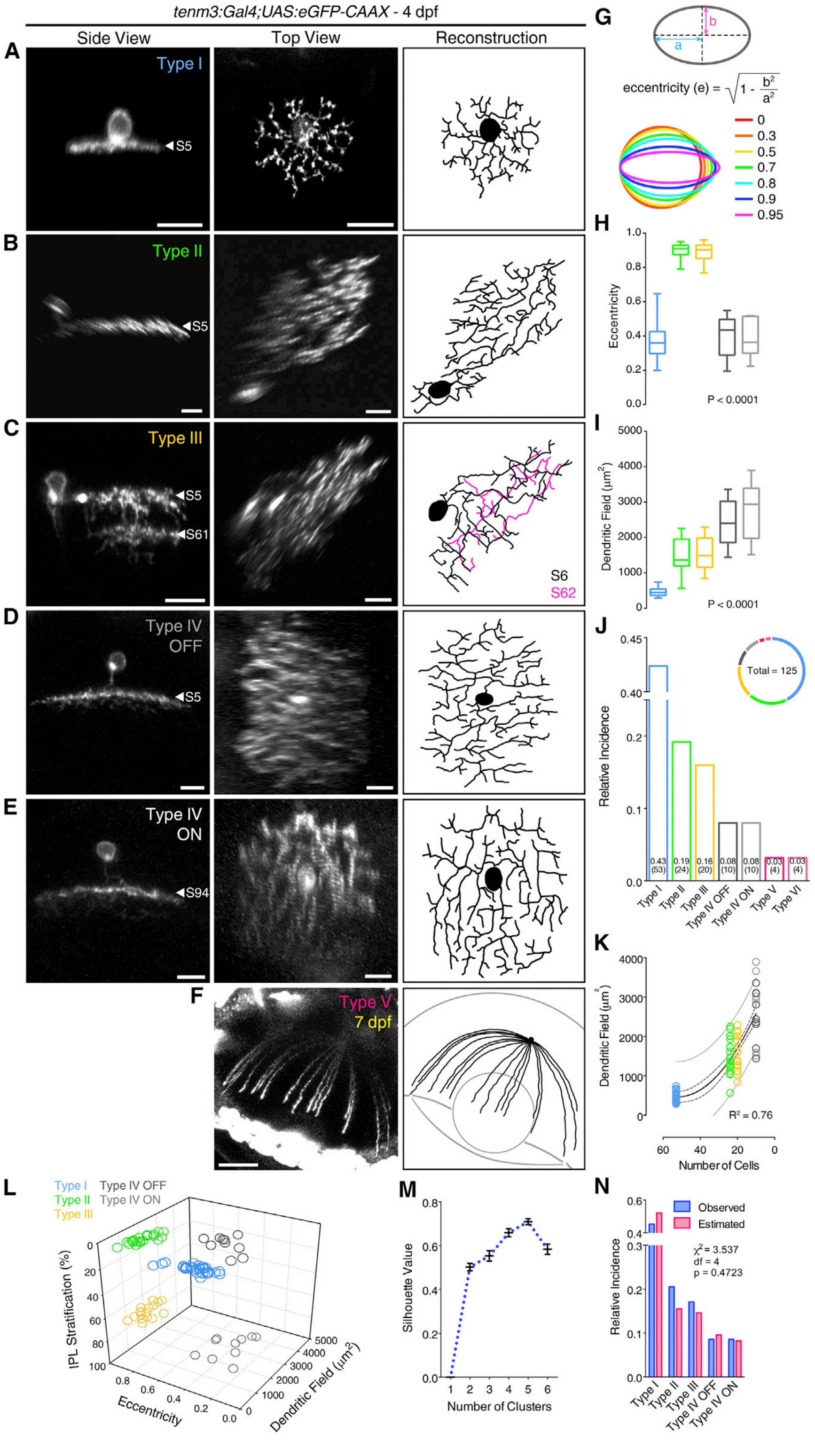

Figure 5. Morphological Diversity of Individual Tenm3⁺ AC Types

(A–F) Morphologies of single *tenm3*⁺ ACs expressing eGFP-CAAX in 4-dpf *UAS:eGFP-CAAX*-injected *Tg(tenm3:Gal4)* larvae. The side views (left), top views (middle), and top view 3D reconstructions (right) are shown. IPL strata location of *tenm3*⁺ AC neurites is indicated by the letter "S" followed by the relative IPL position. The 3D reconstructed neurites of the bi-stratified type III *tenm3*⁺ AC are color-coded according to the stratum they are located. Note that the type V *tenm3*⁺ AC shown in (F) is from a 7-dpf larva. Scale bars, 10 µm in (A)–(E) and 40 µm in (F).

(G) Diagram illustrating the quantification of dendritic field elongation by calculating the eccentricity of dendritic arbor profiles following ellipse fitting. "a" is the length of the semi-major axis, and "b" is the length of the semi-minor axis.

(H–J) Dendritic field elongation (i.e., eccentricity; H), dendritic field area (I), and relative frequency (J) of identified *tenm3*⁺ AC types (n = 125 cells from 65 larvae). The number of observed cells for each *tenm3*⁺ AC type is reported in brackets (J). Boxplots indicate interquartile ranges (boxes), medians (lines in boxes), and ranges (min-max, whiskers). p values are the results of one-way ANOVA.

(K) Relationship between dendritic field area of type I–IV *tenm3*⁺ ACs and their observed frequency (in number of cells). The continuous curve shows the nonlinear regression of the data with a second order polynomial function indicating a quadratic relationship between the two variables. Thick and thin dashed curves report the 95% confidence and prediction bands of the nonlinear fit, respectively. Goodness of fit value (R^2) is reported at the bottom-right corner.

(L and M) k-means clustering of type I–IV *tenm3*⁺ ACs based on their IPL stratification, dendritic field area, and dendritic field elongation. Individual cell data points are color coded according to which *tenm3*⁺ AC type they have been classified (L). Analysis of mean silhouette values for increasing number of clusters indicates that five clusters best describe the dataset (M). Importantly, the five cell clusters obtained by k-means are consistent with the classification of the most frequent *tenm3*⁺ ACs into five different types. Error bars, ±SEM.

(N) Observed (blue) and estimated (red; assuming a retinal coverage factor of 1) relative frequencies of type I–IV *tenm3*⁺ ACs. Results of the two-tailed chi-square test are reported.

See also Table S1.

orthogonally tuned (i.e., tuned to the orientation orthogonal to the OSGC-preferred orientation).

Given the presence of different *tenm3*⁺ AC types (Figure 5), we asked which ones display high orientation selectivity. We thus performed functional imaging of individually GCaMP6f-labeled *tenm3*⁺ ACs, followed by analyses of their tuning and dendritic field morphology (Figure S7A). Strikingly, the only *tenm3*⁺ ACs that showed stimulus-locked visual responses characterized by high orientation tuning were type II or III ACs (Figure 6F). Their degree of orientation selectivity was correlated with the elongation of their dendritic fields (Figure 6G), and the angular difference between their preferred stimulus orientation and dendritic field orientation was close to zero

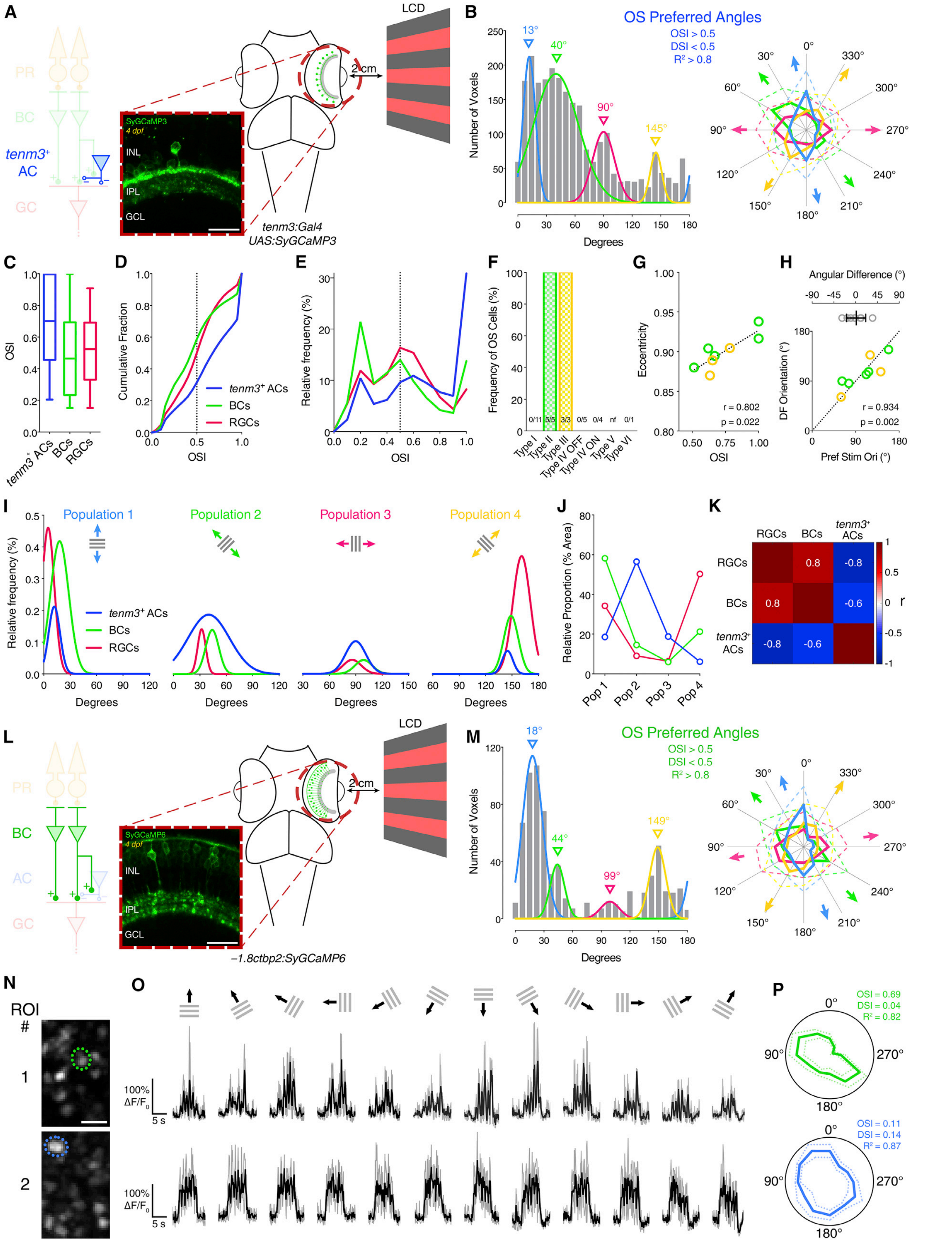

(legend on next page)

(Figure 6H), indicating that type II and III *tenm3*$^+$ ACs respond maximally when the stimulus orientation coincides with the orientation of their dendritic fields. Additionally, the distribution of dendritic field orientations across sparsely eGFP-labeled type II and III *tenm3*$^+$ ACs revealed that they fully cover the orientation space (Figures S7B and S7C). Since blocking $GABA_A$ receptors leads to impaired RGC orientation selectivity (Figures 4G–4L), we investigated whether these two AC types are GABAergic by performing anti-GABA immunostaining of sparsely eGFP-labeled *tenm3*$^+$ ACs. We observed that both type II and III *tenm3*$^+$ ACs do indeed express the neurotransmitter GABA (Figures S7D and S7E), consistent with the results in Figure 4. Together, these data show that type II and III *tenm3*$^+$ ACs constitute a source of orientation-tuned GABAergic inhibition in the retina.

Orientation-Selective Responses in Bipolar Cell Presynaptic Terminals

Since ACs have been shown to modulate BC output at the level of individual presynaptic terminals [37], tuned inhibitory input from type II and III *tenm3*$^+$ ACs could potentially generate orientation tuning in BC presynaptic terminals. We started investigating this idea by performing calcium imaging in the retinae of *Tg(−1.8ctbp2:SyGCaMP6)* larvae, where BC ribbon synapses are selectively labeled with SyGCaMP6 (Figure 6L; Movie S5) [38]. Interestingly, we observed that a fraction of BC responses (~5% of visually responsive voxels) is indeed highly orientation selective (OSI > 0.5, DSI < 0.5, R^2 > 0.8; Figures 6N–6P and S6E–S6H). Similarly to OSGCs and *tenm3*$^+$ ACs, the preferred stimulus orientations of OS responses fall into four subpopulations tuned to gratings oriented along the cardinal (18°, 99°) and diagonal axes (44°, 149°; Figure 6M). The degree of orientation selectivity across the whole population of BC terminals appeared to be more similar to RGCs than *tenm3*$^+$ ACs (Figures 6C–6E). Furthermore, the frequency distribution of the four OS BC subpopulations is highly correlated with that of OSGCs but inversely correlated with *tenm3*$^+$ ACs (Figures 6I–6K). This therefore suggests that orientation selectivity in BC terminals could be generated by orthogonal orientation-tuned inhibitory input from *tenm3*$^+$ ACs, similarly to OSGCs.

DISCUSSION

The vertebrate retina extracts information from visual scenes and sends it to higher brain areas through feature-specific neural pathways. Crucial neural substrates underlying this information processing in the retina are the stereotypic synaptic connections between defined neural cell types. How specific elements of the retinal circuit perform computations is, however, largely unknown. The data presented here define cellular and molecular building blocks of a circuit in the larval zebrafish retina capable of detecting the orientation of elongated visual stimuli. In particular, we take advantage of the functional link between RGC orientation selectivity and the cell-adhesion molecule Tenm3 to genetically identify a class of ACs with elongated dendritic arbors that show orientation tuning. We reveal that these *tenm3*$^+$ ACs and their GABAergic inhibitory output are crucial for the tuning of orientation-selective RGCs. Moreover, we show that Tenm3 is a key molecular player in both the morphological and functional development of the circuit, and that

Figure 6. Orientation Selectivity in Tenm3$^+$ ACs and BC Terminals

(A) Two-photon functional calcium imaging of *tenm3*$^+$ AC synaptic terminals expressing SyGCaMP3 (green) in 4-dpf *Tg(tenm3:Gal4;UAS:SyGCaMP3)* larvae. Distance of the eye from LCD screen is 2 cm. Recordings are performed from two to four Z-planes (approximately 20 μm total volume thickness). INL, inner nuclear layer; GCL, ganglion cell layer; IPL, inner plexiform layer. Scale bar, 20 μm.

(B) Cumulative histogram summarizing the incidence of preferred angles for identified *tenm3*$^+$ AC OS voxels in 4-dpf larvae (n = 20 larvae). Overlaid curves are the fitted Gaussian distributions for each OS subtype. Polar plots illustrate the mean (+1 SD) normalized response profiles for each OS subtype.

(C–E) Degree of orientation selectivity (quantified by the OSI) across voxels with DSI < 0.5 and R^2 > 0 in *tenm3*$^+$ ACs (blue, n = 20 larvae), BCs (green, n = 20 larvae), and RGCs (red, n = 23 larvae; data from Figure 1H). Boxplots in (C) indicate interquartile ranges (boxes), medians (lines in boxes), and 10–90 percentiles (whiskers). The black dotted lines in (D) and (E) indicate the OSI threshold used to identify OS responses (OSI > 0.5).

(F) Bar histogram summarizing the frequency of OS cells among *tenm3*$^+$ ACs in 4-dpf *Tg(tenm3:Gal4)* larvae injected with *UAS:GCaMP6f* DNA constructs (n = 29 cells from 27 larvae). The number of observed OS cells for each *tenm3*$^+$ AC type is reported at the bottom. nf, not found.

(G) Scatterplot representing the relationship between OSI and dendritic field eccentricity of OS type II and III *tenm3*$^+$ ACs (II, n = 5 cells; III, n = 3 cell). Spearman's correlation coefficient (r) with the corresponding p value is reported. Dotted line represents the linear regression fit to the data.

(H) Scatterplot representing the relationship between preferred stimulus orientation and dendritic field orientation of OS type II and III *tenm3*$^+$ ACs (II, n = 5 cells; III, n = 3 cell). Spearman's correlation coefficient (r) with the corresponding p value is reported. Dotted reference line indicates x = y. Top graph shows the angular difference between preferred stimulus orientation and dendritic field orientation (mean ± SD).

(I) Normalized frequency distributions of preferred stimulus orientations in OS *tenm3*$^+$ ACs (blue, n = 20 larvae), BCs (green, n = 20 larvae), and RGCs (red, n = 23 larvae; data from Figure 1J). The Gaussian distributions of the four different OS subpopulations are reported in separate graphs.

(J) Relative proportions of the four different OS subpopulations (Pop 1–4) in *tenm3*$^+$ ACs (blue), BCs (green), and RGCs (red). Values are obtained by calculating the relative proportion (%) of the area under the normalized Gaussian curves in (I).

(K) Correlation matrix showing Spearman's correlation coefficients (r) between the frequency distribution of the four OS subpopulations in *tenm3*$^+$ ACs, BCs, and RGCs.

(L) Two-photon functional calcium imaging of BC ribbon synapses expressing SyGCaMP6 (green) in 4-dpf *Tg(−1.8ctbp2:SyGCaMP6)* larvae. Distance of the eye from LCD screen is 2 cm. Recordings are performed from two to four Z-planes (approximately 20 μm total volume thickness). Scale bar, 20 μm.

(M) Cumulative histogram summarizing the incidence of preferred angles for identified BC OS voxels in 4-dpf larvae (n = 20 larvae). Overlaid curves are the fitted Gaussian distributions for each OS subtype. Polar plots illustrate the mean (+1 SD) normalized response profiles for each OS subtype.

(N–P) Examples of visual responses to moving gratings from two BC terminals. Images showing the mean fluorescence across tuning experiments with identified regions of interest (ROIs) are reported in (N). (O) shows calcium responses of the two ROIs in (N) with black traces representing the average responses across three trials (gray traces) for each stimulus epoch. Polar plots in (P) illustrate the mean response profile (±SD, dotted lines) of each ROI with corresponding OSI, DSI, and R^2 values. Note that ROI #1 shows orientation selectivity. Scale bar, 5 μm.

See also Figures S6 and S7, Table S1, and Movies S4 and S5.

orientation selectivity is also present among bipolar cell presynaptic terminals. Our study represents, to our knowledge, the most extensive characterization of the retinal orientation-selective circuit in a single tractable system. By collecting functional and structural data from amacrine, bipolar, and ganglion cells at cellular and population levels, we provide a mechanistic explanation of how defined neural cell types in the retina generate a fundamental property of visual perception—i.e., orientation selectivity. Additionally, our results elucidate the functional role of two novel AC types, therefore shedding some light on the most diverse and least understood retinal cell class [39].

A Circuit Model of Orientation Selectivity in the Retina

To integrate our data into a general framework describing the computation of orientation selectivity in RGCs, we outlined a model of the retinal OS circuit. The model is based on the following principles: (1) the highly elongated dendritic fields of type II and III *tenm3*+ ACs identified in this study (Figure 5) underlie their orientation tuning (Figures 6 and 7A). Specifically, these defined AC types respond maximally when the orientation of elongated visual stimuli coincides with the orientation of their dendritic fields. Interestingly, ACs characterized by elongated dendritic fields and orientation selectivity have been found also in the rabbit retina, although their genetic identity is still unknown [13, 14]. (2) Type II and III *tenm3*+ ACs provide orthogonal orientation-tuned inhibitory input to OSGCs and, potentially, BC presynaptic terminals (Figures 6I–6K). This feedforward inhibition is mediated by GABA and generates orientation selectivity in OSGCs (Figures 3, 4, and 7A). Interestingly, pharmacological block of synaptic inhibition onto zebrafish BC terminals indicates that orientation selectivity in BC ribbon synapses is generated through AC inhibitory input (J. Johnston and L. Lagnado, personal communication), therefore supporting the idea that the OS inhibitory output of type II and III *tenm3*+ ACs could be at the basis of orientation selectivity in both BCs and OSGCs. Studies in the rabbit and mouse retina showed that OSGCs receive preferred orientation-tuned excitatory input and orthogonal orientation-tuned inhibitory input and, in rabbit, presynaptic GABAergic inhibition plays a pivotal role in the emergence of these OS inputs [9, 40]. Recent findings in *Drosophila* showed an analogous requirement of GABA signaling for orientation

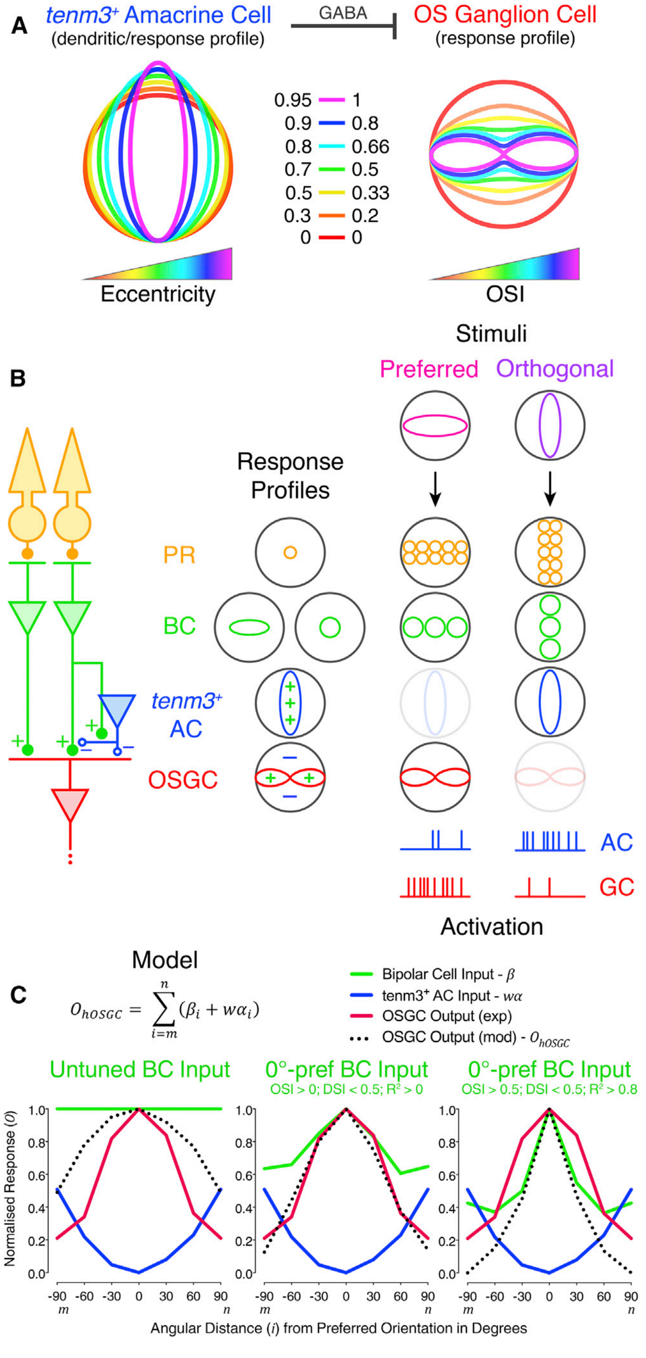

Figure 7. Circuit Model of Orientation Selectivity in the Retina

(A) Hypothesized principles underlying the emergence of orientation selectivity in the retina. The high dendritic field elongation (quantified by the eccentricity of fitted elliptic profiles) of defined *tenm3*+ AC types is at the basis of their high orientation tuning (left). Maximal activation of tuned AC types is obtained when the orientation of elongated visual stimuli coincides with the orientation of their dendritic fields. As a result, these tuned *tenm3*+ AC types generate orientation selectivity in RGCs (quantified by the OSI, right) by providing orthogonal orientation-tuned GABAergic inhibitory input. The color code describes the different levels of dendritic field elongation (left) and orientation selectivity (right).

(B) Examples of retinal OS circuit activation patterns for horizontal orientation-tuned OSGC preferred (magenta) and orthogonal (purple) stimuli. Excitatory input is indicated by "+" (full circles), whereas inhibitory input is indicated by "–" (empty circles). Putative synapses between OS *tenm3*+ ACs and BC terminals are also represented. Tuning profiles of example photoreceptor (PR), bipolar cells (BCs), OS *tenm3*+ amacrine cell (AC), and orientation-selective ganglion cell (OSGC) are also reported.

(C) Simulation of the OSGC tuning profile (O_{hOSGC}, black dotted line) using experimentally observed average response profiles of orthogonally tuned OS *tenm3*+ ACs (α, blue line; n = 20 larvae) and BC terminals (β, green line; n = 20 larvae). Three different orientation-tuning levels of excitatory BC input were used: untuned (left), weakly tuned to preferred orientation (middle) and highly tuned to preferred orientation (right). The experimentally observed average response profile of OSGCs (n = 23 larvae) is shown in red. The algorithm used for the simulation is reported at the top with the related legend. Note that, since the OS *tenm3*+ AC input (α) is inhibitory, a negative synaptic weight factor (*w*) is used in the algorithm. The orientation space ranges from "*m*" to "*n*", which are negative (−90°) and positive (90°) angles orthogonal to the preferred orientation (0°), respectively. Exp, experimental; mod, model; pref, preferred.

See also Figure S5.

selectivity [5], revealing strikingly similar mechanisms between vertebrates and invertebrates. (3) Stimulus orientation, not the axis of stimulus movement, is the visual feature OSGCs are selective to. This is supported by our observation that static gratings, even though less effective in eliciting RGC responses, produce results analogous to those obtained using moving gratings (Figures S5G–S5J). Again, such property has been observed in rabbit and mouse OSGCs as well [10, 40]. Additional mechanisms to those described here may contribute to the emergence of RGC orientation selectivity.

In a schematic example of our model (Figure 7B), when the retina is stimulated with the OSGC-preferred stimulus orientation, the orthogonally tuned *tenm3*$^+$ AC is weakly activated, therefore allowing the OSGC to fire action potentials. When the orthogonal stimulus orientation is presented, instead, the orthogonally tuned *tenm3*$^+$ AC is strongly activated, and, consequently, OSGC firing is inhibited. To further evaluate our model, we implemented the basic principles described above into a simple simulation of OSGC output (Figure 7C). To simulate the OSGC tuning profile (black dotted line), we used our experimentally observed average response profiles of OS *tenm3*$^+$ ACs (blue line) and BC presynaptic terminals (green line). We assumed the OS inhibitory input provided by *tenm3*$^+$ ACs has a subtractive effect on OSGC output and tested three different orientation-tuning levels of excitatory BC input. Interestingly, we found that the average OSGC response profile observed experimentally (red line) was best reproduced when linearly integrating highly OS (OSI > 0.5, DSI < 0.5, $R^2 > 0.8$) orthogonal orientation-tuned inhibitory input from *tenm3*$^+$ ACs and weakly OS (OSI > 0, DSI < 0.5, $R^2 > 0$) preferred orientation-tuned excitatory input from BCs (Figure 7C), indicating that OSGCs may receive BC input characterized by a substantial degree of heterogeneity in orientation tuning. This simulation also implies that OSGCs potentially integrate tuned input from both ACs and BCs to obtain the orientation selectivity observed in vivo. Our results show that the tuned GABAergic inhibitory output of *tenm3*$^+$ ACs is necessary to generate normal RGC orientation selectivity. However, further experiments are needed to precisely determine the relative contribution played by inhibitory AC versus excitatory BC tuned inputs in OSGCs. The strong similarities found between the OS circuit we characterize in the zebrafish retina and previous descriptions of orientation selectivity in mammalian retinae [9, 13, 40] suggest that our model can be generalized to other vertebrate species.

Functional Significance of Orientation Selectivity

The widespread presence of orientation-selective cells in visual systems of many animals highlights the prominent functional role of orientation selectivity in visual perception. Studies on the statistical properties of natural scenes indicate that natural images can be described by local, oriented filters similar to the receptive fields of OS cells found in visual systems [41]. One striking example in humans is the key role played by horizontal visual information in the identification of faces [42]. However, a central question is, where does orientation selectivity emerge in visual circuits? Interestingly, both in vertebrates and invertebrates the detection of elongated visual stimuli takes place early in visual processing [5, 10]. Even in mammalian species, including mice and monkeys, where for long time it was thought that orientation selectivity is an emergent property generated in primary visual cortex [1, 43], OS cells have been found in non-cortical areas such as the lateral geniculate nucleus [6–8] and superior colliculus [44], as well as in the retina [11, 12, 40, 45].

In our study, we found orientation selectivity in presynaptic terminals of BCs and ACs (Figures 6 and S6), which are neurons only one and two synapses away from photoreceptors, respectively. Additionally, we observed OS responses in these cells as early as 4 dpf, when zebrafish larvae start performing visually guided behaviors, such as the optokinetic reflex. Importantly, our data show that the cells and mechanisms underlying RGC orientation selectivity are different from those generating direction selectivity, in line with the notion that parallel retinal circuits process these two distinct visual features. This idea is further supported by the fact that zebrafish OSGC and DSGC axonal projections terminate in different, non-overlapping neuropil laminae of the optic tectum [4, 29].

Role of Teneurins in Neural Circuit Wiring

Teneurins are phylogenetically conserved type II transmembrane proteins with large extracellular domains that are highly expressed in neural tissues [24, 25]. In vertebrates, the teneurin family comprises four members, Tenm1–4, whereas in invertebrates fewer members have been identified (one in *C. elegans*, two in *Drosophila*). In both vertebrate and invertebrate species, teneurins interact in *trans* through both homo- and heterophilic mechanisms [22, 23, 26, 27]. Notably, these trans-interactions are crucial in mediating cell-cell recognition and adhesion. Elegant studies in *Drosophila* demonstrated that teneurins play an instructive role in the synaptic matching between specific pre- and postsynaptic cells [22, 23]. In addition, teneurins regulate other fine-scale neural wiring processes in vivo, such as cell-type-specific dendrite morphogenesis [17, 46], synapse organization [23, 47], and axon projection topography and lamination [17, 48]. The precise roles played by homo- versus heterophilic trans-interactions during these wiring events are still unclear. However, it appears that homophilic interactions are crucial for the initial recognition and matching between specific subsets of neurons [22, 26], whereas heterophilic interactions are involved in subsequent steps of synapse adhesion and organization [27, 28, 47]. Since teneurins can control these distinct processes even between the same sets of neurons [22, 47], sophisticated genetic manipulations will be required to disentangle the contribution of homo- versus heterophilic trans-interactions in neurons where a given teneurin and its heterophilic binding partners are simultaneously expressed.

Our data suggest that Tenm3 specifies the correct development of functionally and morphologically defined subsets of ACs and RGCs forming a circuit underlying retinal orientation selectivity. Even though our results are suggestive of direct synaptic matching between *tenm3*$^+$ ACs and OSGCs, the technical limitations of our study do not allow to unequivocally demonstrate the physical synaptic connections between these two neural populations, and, therefore, future experiments will be required to further elucidate this point. Given that Tenm3 is expressed in both ACs and RGCs (Figures S1C–S1F, S2B, and S2C) [17] and that Tenm3 loss of function leads to defects in *tenm3*$^+$ AC neurite IPL stratification (Figures 2B–2D) as well as specific morphological and functional impairments in RGCs

(Figures 1 and S1) [17], one possible explanation of Tenm3 mechanism of action could be through *trans*-synaptic homophilic interactions. However, loss of selective trans-interactions with other cell-adhesion molecules known to bind heterophilically with teneurins, such as latrophilins [27, 28], may as well explain the phenotypes we observed in $tenm3^{KO}$ mutants. Interestingly, some latrophilins are expressed in the zebrafish eye at larval and adult stages, although it is not clear whether they exhibit a cell-type-specific expression pattern [49, 50]. Thus, the retinal orientation-selective circuit characterized in this study represents a tractable in vivo vertebrate system to test the specific roles played by teneurin homo- and heterophilic trans-interactions during neural circuit wiring.

In conclusion, our findings constitute a significant advancement in the understanding of how orientation selectivity emerges in the vertebrate retina, bringing together molecular markers, cell morphologies, pharmacology, and function. Moreover, the in vivo system and relative genetic tools established in this study will allow investigations of the precise functional role played by retinal orientation selectivity in higher visual areas of the brain as well as its role in performing visually guided behaviors.

EXPERIMENTAL PROCEDURES

Statistical test results are reported in the figures and figure legends. Statistical analyses and tests were carried out using Prism 6 (GraphPad), SigmaPlot 11 (Systat Software), or MATLAB R2014b (MathWorks). A comprehensive description of the statistical analyses and tests performed in this study can be found in Table S1. Before performing statistical tests, descriptive statistics (e.g., normality tests to see whether values come from a Gaussian distribution or F-test to compare variances) were used to choose the appropriate statistical test (reported in Table S1). The criterion for statistical significance was set at $p < 0.05$. In order to quantitatively measure and assess the effects of treatments or genetic manipulations between animal groups, the effect size (Cohen's *d*) and its 95% confidence interval were also calculated (see Table S1). See Supplemental Experimental Procedures for detailed methods and zebrafish lines used in this study. All animal procedures were approved by the local Animal Welfare and Ethics Review Body (King's College London) and were carried out in accordance with the Animals (Scientific Procedures) Act 1986 under license from the United Kingdom Home Office.

SUPPLEMENTAL INFORMATION

Supplemental Information includes seven figures, Supplemental Experimental Procedures, one table, and five movies and can be found with this article online at http://dx.doi.org/10.1016/j.cub.2016.05.035.

AUTHOR CONTRIBUTIONS

P.A. and R.H. designed the study. P.A. performed the experiments and analyzed the data. O.S. and C.M. generated the TALEN mutant zebrafish. P.A. and R.H. wrote the manuscript.

ACKNOWLEDGMENTS

We would like to thank Martin Meyer and Leon Lagnado for providing zebrafish transgenic lines, Oscar Marín and Martin Meyer for comments on the manuscript, and Andrew Lowe for analysis tools. We thank the Nikon Imaging Centre at King's College London for support and instrumentation. This study was supported by a BBSRC grant (BB/M000664/1) awarded to R.H. and a King's College London Health Schools PhD studentship awarded to P.A., sponsored by the Medical Research Council (1413592). O.S. and C.M. are part of a Genome Editing Team funded by a Wellcome Trust Technology Development Grant (WT093389) to Corinne Houart, Jon Clarke, Uwe Drescher, Simon Hughes, and R.H.

Received: March 22, 2016
Revised: April 21, 2016
Accepted: May 12, 2016
Published: June 30, 2016

REFERENCES

1. Hubel, D.H., and Wiesel, T.N. (1962). Receptive fields, binocular interaction and functional architecture in the cat's visual cortex. J. Physiol. *160*, 106–154.
2. Hubel, D.H., and Wiesel, T.N. (1968). Receptive fields and functional architecture of monkey striate cortex. J. Physiol. *195*, 215–243.
3. Niell, C.M., and Stryker, M.P. (2008). Highly selective receptive fields in mouse visual cortex. J. Neurosci. *28*, 7520–7536.
4. Nikolaou, N., Lowe, A.S., Walker, A.S., Abbas, F., Hunter, P.R., Thompson, I.D., and Meyer, M.P. (2012). Parametric functional maps of visual inputs to the tectum. Neuron *76*, 317–324.
5. Fisher, Y.E., Silies, M., and Clandinin, T.R. (2015). Orientation selectivity sharpens motion detection in *Drosophila*. Neuron *88*, 390–402.
6. Cheong, S.K., Tailby, C., Solomon, S.G., and Martin, P.R. (2013). Cortical-like receptive fields in the lateral geniculate nucleus of marmoset monkeys. J. Neurosci. *33*, 6864–6876.
7. Piscopo, D.M., El-Danaf, R.N., Huberman, A.D., and Niell, C.M. (2013). Diverse visual features encoded in mouse lateral geniculate nucleus. J. Neurosci. *33*, 4642–4656.
8. Marshel, J.H., Kaye, A.P., Nauhaus, I., and Callaway, E.M. (2012). Anterior-posterior direction opponency in the superficial mouse lateral geniculate nucleus. Neuron *76*, 713–720.
9. Venkataramani, S., and Taylor, W.R. (2010). Orientation selectivity in rabbit retinal ganglion cells is mediated by presynaptic inhibition. J. Neurosci. *30*, 15664–15676.
10. Levick, W.R. (1967). Receptive fields and trigger features of ganglion cells in the visual streak of the rabbits retina. J. Physiol. *188*, 285–307.
11. Zhao, X., Chen, H., Liu, X., and Cang, J. (2013). Orientation-selective responses in the mouse lateral geniculate nucleus. J. Neurosci. *33*, 12751–12763.
12. Baden, T., Berens, P., Franke, K., Román Rosón, M., Bethge, M., and Euler, T. (2016). The functional diversity of retinal ganglion cells in the mouse. Nature *529*, 345–350.
13. Bloomfield, S.A. (1994). Orientation-sensitive amacrine and ganglion cells in the rabbit retina. J. Neurophysiol. *71*, 1672–1691.
14. Murphy-Baum, B.L., and Taylor, W.R. (2015). The synaptic and morphological basis of orientation selectivity in a polyaxonal amacrine cell of the rabbit retina. J. Neurosci. *35*, 13336–13350.
15. Masland, R.H. (2012). The neuronal organization of the retina. Neuron *76*, 266–280.
16. Roska, B., and Werblin, F. (2001). Vertical interactions across ten parallel, stacked representations in the mammalian retina. Nature *410*, 583–587.
17. Antinucci, P., Nikolaou, N., Meyer, M.P., and Hindges, R. (2013). Teneurin-3 specifies morphological and functional connectivity of retinal ganglion cells in the vertebrate visual system. Cell Rep. *5*, 582–592.
18. Yamagata, M., and Sanes, J.R. (2008). Dscam and Sidekick proteins direct lamina-specific synaptic connections in vertebrate retina. Nature *451*, 465–469.
19. Krishnaswamy, A., Yamagata, M., Duan, X., Hong, Y.K., and Sanes, J.R. (2015). Sidekick 2 directs formation of a retinal circuit that detects differential motion. Nature *524*, 466–470.
20. Duan, X., Krishnaswamy, A., De la Huerta, I., and Sanes, J.R. (2014). Type II cadherins guide assembly of a direction-selective retinal circuit. Cell *158*, 793–807.

21. Sun, L.O., Jiang, Z., Rivlin-Etzion, M., Hand, R., Brady, C.M., Matsuoka, R.L., Yau, K.W., Feller, M.B., and Kolodkin, A.L. (2013). On and off retinal circuit assembly by divergent molecular mechanisms. Science *342*, 1241974.
22. Hong, W., Mosca, T.J., and Luo, L. (2012). Teneurins instruct synaptic partner matching in an olfactory map. Nature *484*, 201–207.
23. Mosca, T.J., Hong, W., Dani, V.S., Favaloro, V., and Luo, L. (2012). Trans-synaptic Teneurin signalling in neuromuscular synapse organization and target choice. Nature *484*, 237–241.
24. Tucker, R.P., and Chiquet-Ehrismann, R. (2006). Teneurins: a conserved family of transmembrane proteins involved in intercellular signaling during development. Dev. Biol. *290*, 237–245.
25. Mosca, T.J. (2015). On the Teneurin track: a new synaptic organization molecule emerges. Front. Cell. Neurosci. *9*, 204.
26. Beckmann, J., Schubert, R., Chiquet-Ehrismann, R., and Müller, D.J. (2013). Deciphering teneurin domains that facilitate cellular recognition, cell-cell adhesion, and neurite outgrowth using atomic force microscopy-based single-cell force spectroscopy. Nano Lett. *13*, 2937–2946.
27. Boucard, A.A., Maxeiner, S., and Südhof, T.C. (2014). Latrophilins function as heterophilic cell-adhesion molecules by binding to teneurins: regulation by alternative splicing. J. Biol. Chem. *289*, 387–402.
28. Silva, J.P., Lelianova, V.G., Ermolyuk, Y.S., Vysokov, N., Hitchen, P.G., Berninghausen, O., Rahman, M.A., Zangrandi, A., Fidalgo, S., Tonevitsky, A.G., et al. (2011). Latrophilin 1 and its endogenous ligand Lasso/teneurin-2 form a high-affinity transsynaptic receptor pair with signaling capabilities. Proc. Natl. Acad. Sci. USA *108*, 12113–12118.
29. Lowe, A.S., Nikolaou, N., Hunter, P.R., Thompson, I.D., and Meyer, M.P. (2013). A systems-based dissection of retinal inputs to the zebrafish tectum reveals different rules for different functional classes during development. J. Neurosci. *33*, 13946–13956.
30. Chen, T.W., Wardill, T.J., Sun, Y., Pulver, S.R., Renninger, S.L., Baohan, A., Schreiter, E.R., Kerr, R.A., Orger, M.B., Jayaraman, V., et al. (2013). Ultrasensitive fluorescent proteins for imaging neuronal activity. Nature *499*, 295–300.
31. Bulina, M.E., Chudakov, D.M., Britanova, O.V., Yanushevich, Y.G., Staroverov, D.B., Chepurnykh, T.V., Merzlyak, E.M., Shkrob, M.A., Lukyanov, S., and Lukyanov, K.A. (2006). A genetically encoded photosensitizer. Nat. Biotechnol. *24*, 95–99.
32. Ahrens, M.B., Orger, M.B., Robson, D.N., Li, J.M., and Keller, P.J. (2013). Whole-brain functional imaging at cellular resolution using light-sheet microscopy. Nat. Methods *10*, 413–420.
33. Hunter, P.R., Lowe, A.S., Thompson, I.D., and Meyer, M.P. (2013). Emergent properties of the optic tectum revealed by population analysis of direction and orientation selectivity. J. Neurosci. *33*, 13940–13945.
34. Xi, Y., Yu, M., Godoy, R., Hatch, G., Poitras, L., and Ekker, M. (2011). Transgenic zebrafish expressing green fluorescent protein in dopaminergic neurons of the ventral diencephalon. Dev. Dyn. *240*, 2539–2547.
35. Vaney, D.I., Sivyer, B., and Taylor, W.R. (2012). Direction selectivity in the retina: symmetry and asymmetry in structure and function. Nat. Rev. Neurosci. *13*, 194–208.
36. Dowling, J.E., and Ehinger, B. (1978). The interplexiform cell system. I. Synapses of the dopaminergic neurons of the goldfish retina. Proc. R. Soc. Lond. B Biol. Sci. *201*, 7–26.
37. Asari, H., and Meister, M. (2012). Divergence of visual channels in the inner retina. Nat. Neurosci. *15*, 1581–1589.
38. Johnston, J., Ding, H., Seibel, S.H., Esposti, F., and Lagnado, L. (2014). Rapid mapping of visual receptive fields by filtered back projection: application to multi-neuronal electrophysiology and imaging. J. Physiol. *592*, 4839–4854.
39. MacNeil, M.A., Heussy, J.K., Dacheux, R.F., Raviola, E., and Masland, R.H. (1999). The shapes and numbers of amacrine cells: matching of photofilled with Golgi-stained cells in the rabbit retina and comparison with other mammalian species. J. Comp. Neurol. *413*, 305–326.
40. Nath, A., and Schwartz, G.W. (2016). Cardinal orientation selectivity is represented by two distinct ganglion cell types in mouse retina. J. Neurosci. *36*, 3208–3221.
41. Olshausen, B.A., and Field, D.J. (1996). Emergence of simple-cell receptive field properties by learning a sparse code for natural images. Nature *381*, 607–609.
42. Dakin, S.C., and Watt, R.J. (2009). Biological "bar codes" in human faces. J Vis *9*, 2.1–10.
43. Niell, C.M. (2013). Vision: more than expected in the early visual system. Curr. Biol. *23*, R681–R684.
44. Wang, L., Sarnaik, R., Rangarajan, K., Liu, X., and Cang, J. (2010). Visual receptive field properties of neurons in the superficial superior colliculus of the mouse. J. Neurosci. *30*, 16573–16584.
45. Passaglia, C.L., Troy, J.B., Rüttiger, L., and Lee, B.B. (2002). Orientation sensitivity of ganglion cells in primate retina. Vision Res. *42*, 683–694.
46. Hattori, Y., Usui, T., Satoh, D., Moriyama, S., Shimono, K., Itoh, T., Shirahige, K., and Uemura, T. (2013). Sensory-neuron subtype-specific transcriptional programs controlling dendrite morphogenesis: genome-wide analysis of Abrupt and Knot/Collier. Dev. Cell *27*, 530–544.
47. Mosca, T.J., and Luo, L. (2014). Synaptic organization of the *Drosophila* antennal lobe and its regulation by the Teneurins. eLife *3*, e03726.
48. Dharmaratne, N., Glendining, K.A., Young, T.R., Tran, H., Sawatari, A., and Leamey, C.A. (2012). Ten-m3 is required for the development of topography in the ipsilateral retinocollicular pathway. PLoS ONE *7*, e43083.
49. Harty, B.L., Krishnan, A., Sanchez, N.E., Schiöth, H.B., and Monk, K.R. (2015). Defining the gene repertoire and spatiotemporal expression profiles of adhesion G protein-coupled receptors in zebrafish. BMC Genomics *16*, 62.
50. Lange, M., Norton, W., Coolen, M., Chaminade, M., Merker, S., Proft, F., Schmitt, A., Vernier, P., Lesch, K.P., and Bally-Cuif, L. (2012). The ADHD-susceptibility gene lphn3.1 modulates dopaminergic neuron formation and locomotor activity during zebrafish development. Mol. Psychiatry *17*, 946–954.

Current Biology, Volume *26*

Supplemental Information

Neural Mechanisms Generating

Orientation Selectivity in the Retina

Paride Antinucci, Oniz Suleyman, Clinton Monfries, and Robert Hindges

SUPPLEMENTAL FIGURES AND LEGENDS

Figure S1

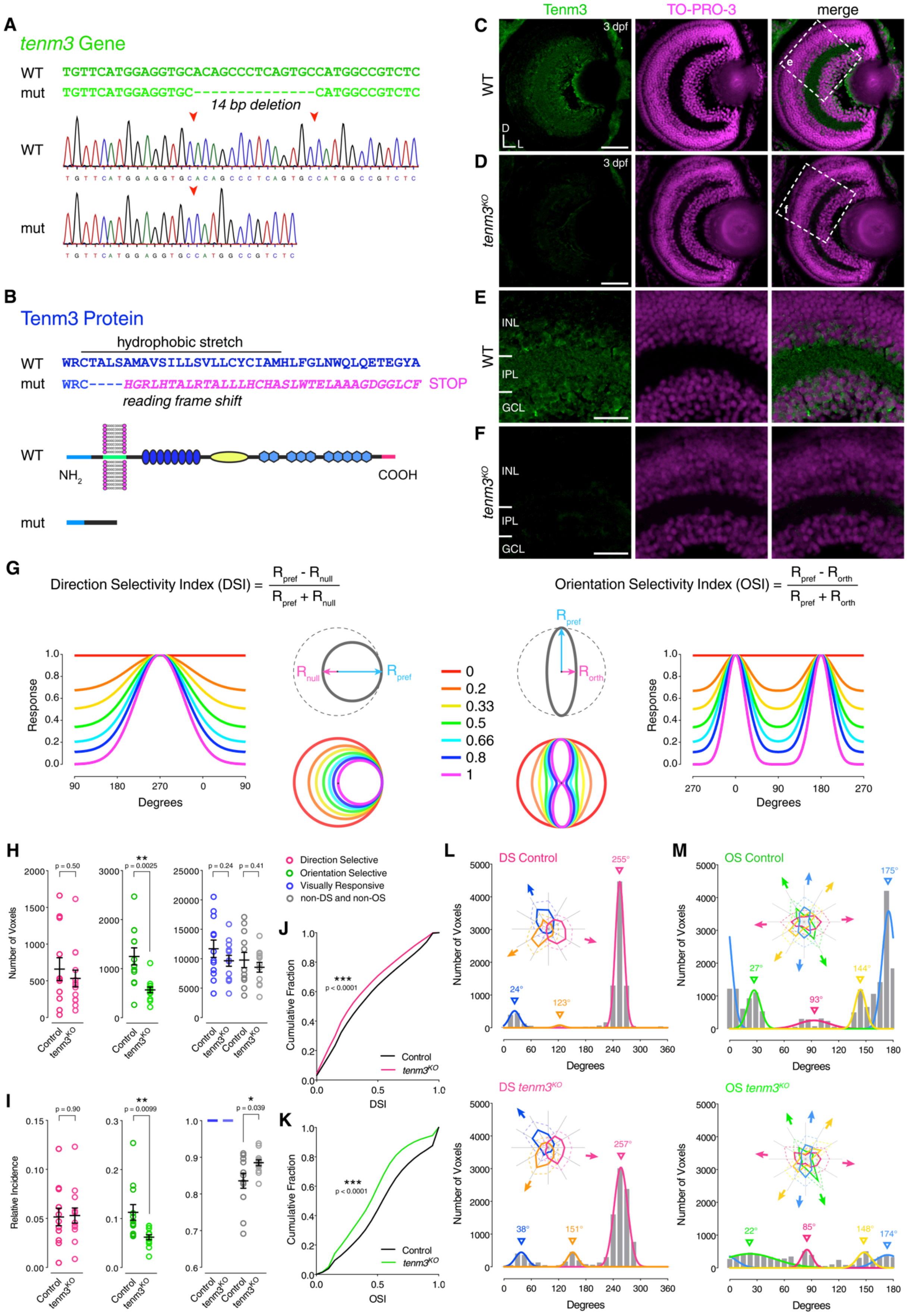

Figure S1. Tenm3 Knock-out, Quantification of Direction and Orientation Selectivity, and Tenm3 Functional Role in Late Development, Related to Figure 1 and Movie S1

(A and B) TALEN-mediated *tenm3* gene knock-out (A) and consequent structural changes in the Tenm3 protein (B). DNA sequencing chromatograms show the 14-base pair (bp) deletion present in *tenm3*^{KO} mutant (mut) larvae. This deletion in the gene region encoding the transmembrane domain of Tenm3 generates a reading frame shift and subsequent premature stop codon, therefore leading to the production of a non-functional Tenm3 protein missing its entire extracellular domain.

(C and D) Immunostaining showing the expression of Tenm3 (green) in retinae of 3-dpf WT (C) and *tenm3*^{KO} larvae (D). Cell bodies are labelled with the nuclear stain TO-PRO-3 (magenta). D, dorsal; L, lateral. Scale bars are 40 μm.

(E and F) Insets in (C) and (D) showing that, in the WT retina (E), Tenm3 is expressed in the inner nuclear layer (INL) and ganglion cell layer (GCL), whereas no Tenm3 expression is present in the *tenm3*^{KO} retina (F). IPL, inner plexiform layer. Scale bars are 20 μm. Importantly, all images in (C-F) were obtained using the same acquisition settings.

(G) Diagram illustrating the quantification of direction selectivity (left) and orientation selectivity (right) using the direction selectivity index (DSI) and orientation selectivity index (OSI), respectively. The algorithms used to calculate DSI and OSI of visual responses are reported at the top. For DSI, the responses to the preferred direction of moving bars (R_{pref}) and opposite, or null, direction (R_{null}; 180° angular distance) are used. For OSI, the responses to the preferred axis (R_{pref}) and orthogonal axis (R_{orth}; 90° angular distance) are used. Note that the arrows indicate the motion direction of orthogonally oriented bars or gratings. A single Gaussian or von-Mises distribution is used to fit responses of direction-selective cells and estimate their preferred direction of motion from the centre of the fitted curve. The sum of two Gaussian or von-Mises distributions (180° angular distance apart) is used to fit responses of orientation-selective cells in order to estimate their preferred axis and stimulus orientation from the centres of the fitted curves. The colour code describes the different levels of direction (left) and orientation (right) selectivity.

(H and I) Average number (H) and relative frequency (I) of DS, OS, visually responsive and non-DS/non-OS voxels per Z-plane in control (n = 12 larvae) and *tenm3*^{KO} (n = 13 larvae) 7-dpf *Tg(isl2b:Gal4;UAS:SyGCaMP3)* larvae. Error bars are ± SEM. *p < 0.05, **p < 0.01, unpaired two-tailed Student's t test.

(J and K) Cumulative distributions of DSI values ($R^2 > 0$) across voxels with OSI < 0.5 (J) and OSI values ($R^2 > 0$) across voxels with DSI < 0.5 (K) in control and *tenm3*^{KO} larvae. ***p < 0.001, two-sample Kolmogorov-Smirnov test.

(L and M) Cumulative histograms summarising the incidence of preferred angles for identified DS (L) and OS voxels (M) in control (n = 12; top) and *tenm3*^{KO} (n = 13; bottom) 7-dpf larvae. Overlaid curves are the fitted Gaussian distributions for each DS or OS subtype. Polar plots illustrate the mean (+ 1 SD) normalised response profiles for each DS or OS subtype.

Figure S2

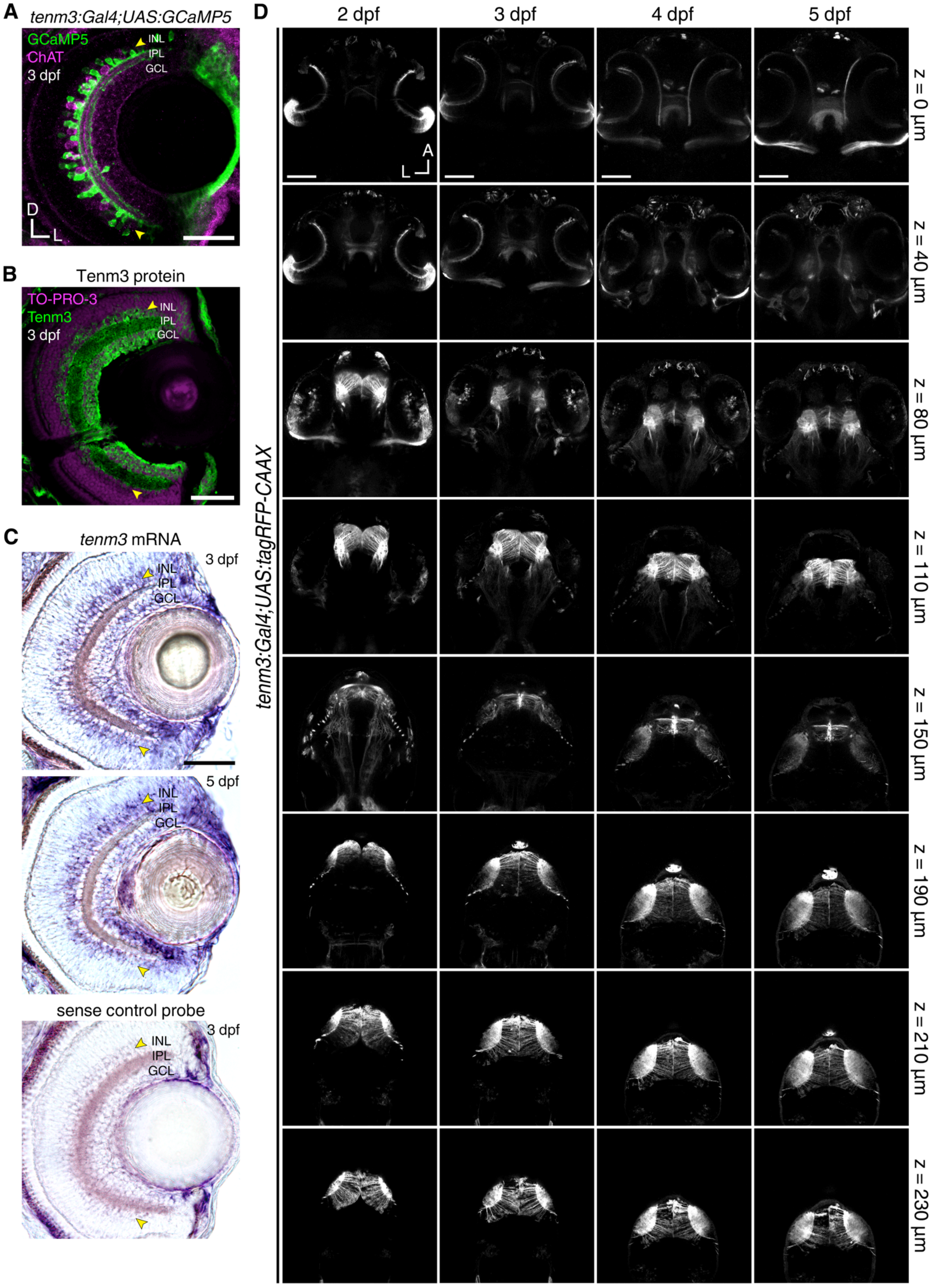

A
tenm3:Gal4;UAS:GCaMP5
GCaMP5
ChAT
3 dpf
INL
IPL
GCL
D
L
B
Tenm3 protein
TO-PRO-3
Tenm3
3 dpf
INL
IPL
GCL
C
tenm3 mRNA
3 dpf
INL
IPL
GCL
5 dpf
INL
IPL
GCL
sense control probe
3 dpf
INL
IPL
GCL
D
2 dpf
3 dpf
4 dpf
5 dpf
A
L
tenm3:Gal4;UAS:tagRFP-CAAX
z = 0 μm
z = 40 μm
z = 80 μm
z = 110 μm
z = 150 μm
z = 190 μm
z = 210 μm
z = 230 μm

Figure S2. ***Tg(tenm3:Gal4)*** **BAC Transgenic Labelling in the Retina and Brain, Related to Figure 2 and Movie S2**

(A) Immunostaining showing *tenm3* BAC transgenic labelling of a subset of amacrine cells in a 3-dpf *Tg(tenm3:Gal4;UAS:GCaMP5G)* larva. GCaMP5 expression is showed in green, whereas choline acetyltransferase (ChAT) expression is showed in magenta as a reference. Yellow arrowheads indicate *tenm3*$^+$ amacrine cells in the inner portion of the inner nuclear layer (INL). GCL, ganglion cell layer; IPL, inner plexiform layer. D, dorsal; L, lateral. Scale bar is 40 μm.

(B) Immunostaining showing the expression of Tenm3 (green) in the retina of a 3-dpf WT larva. Cell bodies are labelled with the nuclear stain TO-PRO-3 (magenta). Yellow arrowheads indicate Tenm3$^+$ amacrine cells in the inner portion of the INL. Scale bar is 40 μm.

(C) *In situ* hybridisations showing *tenm3* mRNA expression in the retinae of 3- and 5-dpf WT larvae (top). A control *in situ* hybridisation at 3 dpf using sense *tenm3* riboprobe is reported at the bottom. Yellow arrowheads indicate the inner portion of the INL. Scale bar is 40 μm. From Antinucci, et al. [S1].

(D) Overview of the brain regions labelled by the *Tg(tenm3:Gal4;UAS:tagRFP-CAAX)* BAC transgenic zebrafish line from 2 to 5 dpf. Eight Z-planes are displayed per developmental stage (approximately 230 μm total volume thickness). The most ventral regions (z = 0 μm) are displayed at the top, whereas the most dorsal ones (z = 230 μm) are at the bottom. Note that tagRFP-CAAX is expressed in the retina (0-80 μm Z-axis depth), thalamus (80-150 μm Z-axis depth), and optic tectum (150-230 μm Z-axis depth), which are brain regions where *tenm3* is highly expressed. A, anterior; L, left. Scale bars are 100 μm.

Figure S3

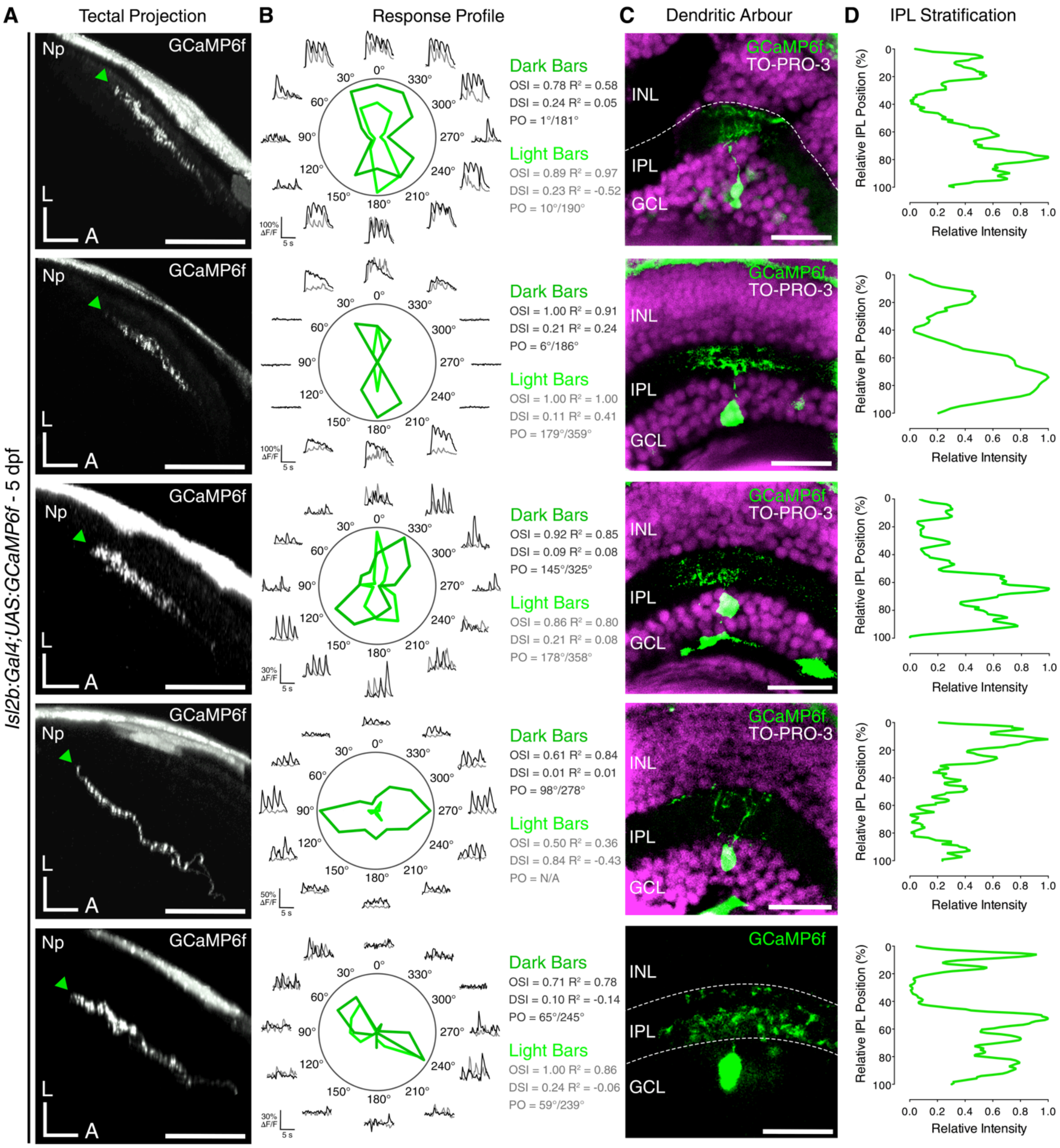

A Tectal Projection
B Response Profile
C Dendritic Arbour
D IPL Stratification
Isl2b:Gal4;UAS:GCaMP6f - 5 dpf
Np
GCaMP6f
L
A
Dark Bars
OSI = 0.78 R² = 0.58
DSI = 0.24 R² = 0.05
PO = 1°/181°
Light Bars
OSI = 0.89 R² = 0.97
DSI = 0.23 R² = -0.52
PO = 10°/190°
Dark Bars
OSI = 1.00 R² = 0.91
DSI = 0.21 R² = 0.24
PO = 6°/186°
Light Bars
OSI = 1.00 R² = 1.00
DSI = 0.11 R² = 0.41
PO = 179°/359°
Dark Bars
OSI = 0.92 R² = 0.85
DSI = 0.09 R² = 0.08
PO = 145°/325°
Light Bars
OSI = 0.86 R² = 0.80
DSI = 0.21 R² = 0.08
PO = 178°/358°
Dark Bars
OSI = 0.61 R² = 0.84
DSI = 0.01 R² = 0.01
PO = 98°/278°
Light Bars
OSI = 0.50 R² = 0.36
DSI = 0.84 R² = -0.43
PO = N/A
Dark Bars
OSI = 0.71 R² = 0.78
DSI = 0.10 R² = -0.14
PO = 65°/245°
Light Bars
OSI = 1.00 R² = 0.86
DSI = 0.24 R² = -0.06
PO = 59°/239°
GCaMP6f
TO-PRO-3
INL
IPL
GCL
Relative IPL Position (%)
Relative Intensity

Figure S3. IPL Dendritic Stratification Pattern of Individual OSGCs, Related to Figure 2

(A) Side views of axon projections (green arrowheads) of individual OSGCs expressing GCaMP6f in the optic tectum of 5-dpf *UAS:GCaMP6f*-injected *Tg(isl2b:Gal4)* larvae. 12.8% of functionally imaged RGCs were OS (5 out of 39 cells in 39 larvae). Np, neuropil; A, anterior; L, lateral. Scale bars are 40 μm.

(B) Functional identification of individual OSGCs through analyses of their visual responses to moving bars. Polar plots represent the tuning profiles (obtained by calculating the integral response to each stimulus) of OSGCs for dark and light moving bars (dark and light green, respectively). Black and grey traces represent the $\Delta F/F_0$ calcium responses to moving dark and light bars, respectively. OSI, orientation selectivity index; DSI, direction selectivity index; R^2, Gaussian goodness of fit; PO, preferred orientation.

(C and D) Immunostaining for GCaMP6f (green) showing the dendritic morphology of functionally identified OSGCs (C) and corresponding normalised IPL fluorescence intensity profiles (D). 0% corresponds to the INL/IPL boundary, whereas 100% corresponds to the IPL/GCL boundary. Cell bodies are labelled with the nuclear stain TO-PRO-3 (magenta). INL, inner nuclear layer; GCL, ganglion cell layer; IPL, inner plexiform layer. Scale bars are 20 μm.

Figure S4

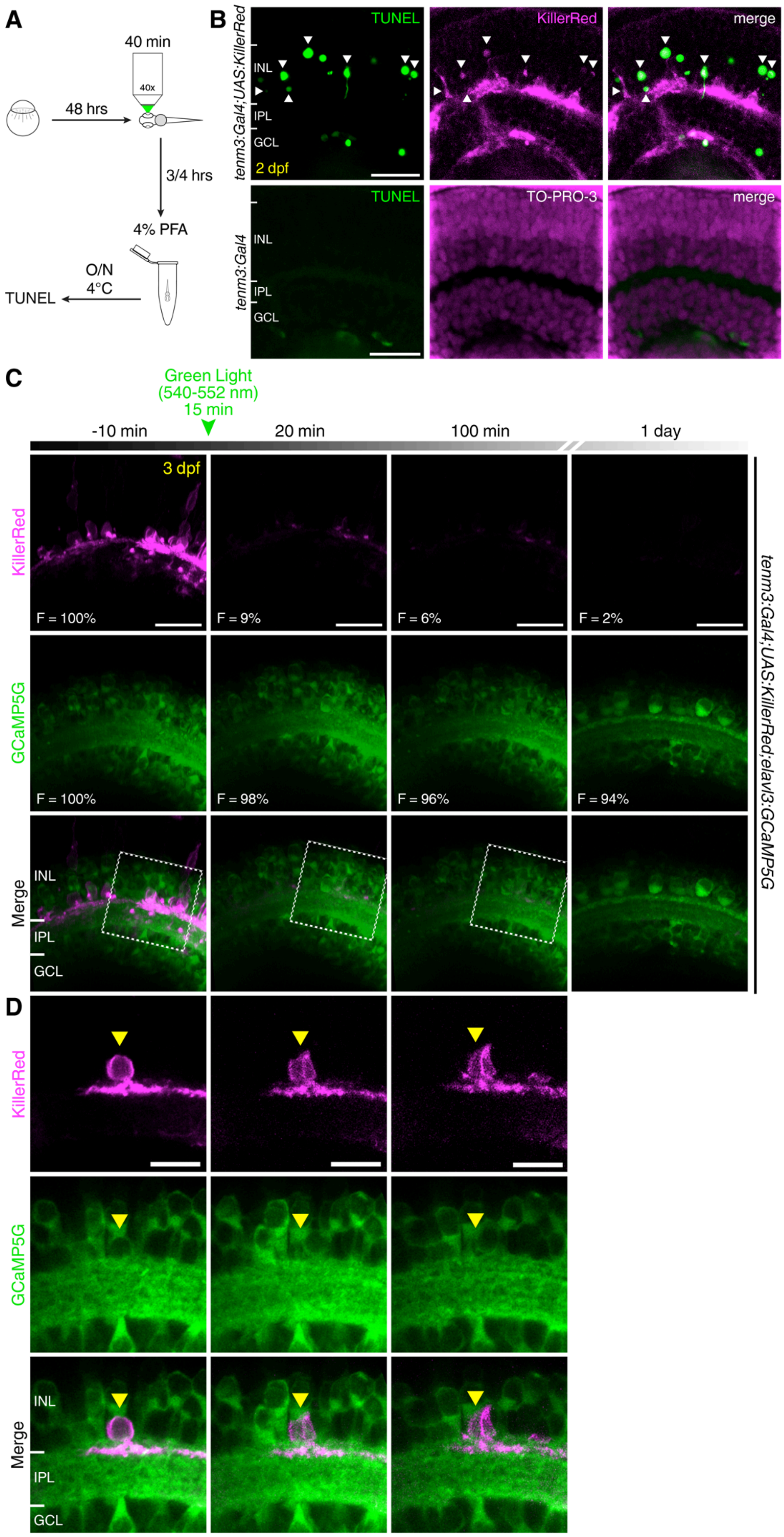
A
40 min
40x
48 hrs
3/4 hrs
4% PFA
O/N
4°C
TUNEL
B
tenm3:Gal4;UAS:KillerRed
TUNEL
KillerRed
merge
INL
IPL
GCL
2 dpf
tenm3:Gal4
TO-PRO-3
C
Green Light
(540-552 nm)
15 min
-10 min
20 min
100 min
1 day
3 dpf
KillerRed
F = 100%
F = 9%
F = 6%
F = 2%
GCaMP5G
F = 100%
F = 98%
F = 96%
F = 94%
Merge
tenm3:Gal4;UAS:KillerRed;elavl3:GCaMP5G
D
KillerRed
GCaMP5G
Merge
INL
IPL
GCL

Figure S4. Optogenetic Ablation of Tenm3⁺ ACs using KillerRed, Related to Figure 3 and Movie S3

(A) Schematic summarising the experimental procedure used to validate the optogenetic ablation of *tenm3*$^+$ ACs. At 2 dpf, the eyes of *Tg(tenm3:Gal4;UAS:KillerRed)* and *Tg(tenm3:Gal4)* control larvae were illuminated with intense green light (540-552 nm) for 40 minutes using a wide-field fluorescence microscope equipped with a 40× objective. After 3-4 hours, the larvae were fixed in 4% paraformaldehyde (PFA) at 4°C overnight (O/N). Subsequently, larvae were cryosectioned and fluorometric terminal deoxynucleotidyl transferase dUTP nick end labeling (TUNEL) assays were performed on sections following standard protocols.

(B) TUNEL staining (green) shows the presence of apoptotic nuclei characteristic of dying cells in the retinae of 2-dpf *Tg(tenm3:Gal4;UAS:KillerRed)* larvae following 40 minutes of green light illumination (top, n = 3 larvae). In these larvae KillerRed (magenta) is selectively expressed in *tenm3*$^+$ ACs. White arrowheads indicate the sites of co-localisation between KillerRed$^+$ cell bodies and TUNEL$^+$ apoptotic nuclei in the inner nuclear layer (INL). Importantly, no cell death is detected in the retinae of 2-dpf *Tg(tenm3:Gal4)* control larvae that were subjected the same illumination procedure (bottom, n = 3 larvae). In the bottom panel, cell bodies are labelled with the nuclear stain TO-PRO-3 (magenta). GCL, ganglion cell layer; IPL, inner plexiform layer. Scale bars are 20 µm.

(C) Proof-of-principle experiment showing the effects of 15 minutes illumination with green light on the KillerRed (magenta) fluorescence level (F) in the retina of a 3-dpf *Tg(tenm3:Gal4; UAS:KillerRed;elavl3:GCaMP5G)* larva. Strikingly, strong photobleaching (~90% decrease in fluorescence) is observed after green light illumination, indicating effective KillerRed phototoxicity. This large decrease in KillerRed fluorescence is visible even one day after illumination. No significant decrease in the overall GCaMP5G fluorescence (green) is detected after illumination. Importantly, all images were obtained using the same acquisition settings. Scale bars are 20 µm.

(D) Inset of (C) showing the dramatic morphological changes of a single KillerRed-expressing *tenm3*$^+$ AC (yellow arrowhead) following green light illumination. Notably, also the GCaMP5G fluorescence appears to decrease in this cell, probably due to the production of phototoxic reactive oxygen species in the cytoplasm. The magenta channel signal has been increased post-acquisition to compensate for the ~90% decrease in fluorescence resulting from the illumination procedure. Scale bars are 10 µm.

Figure S5

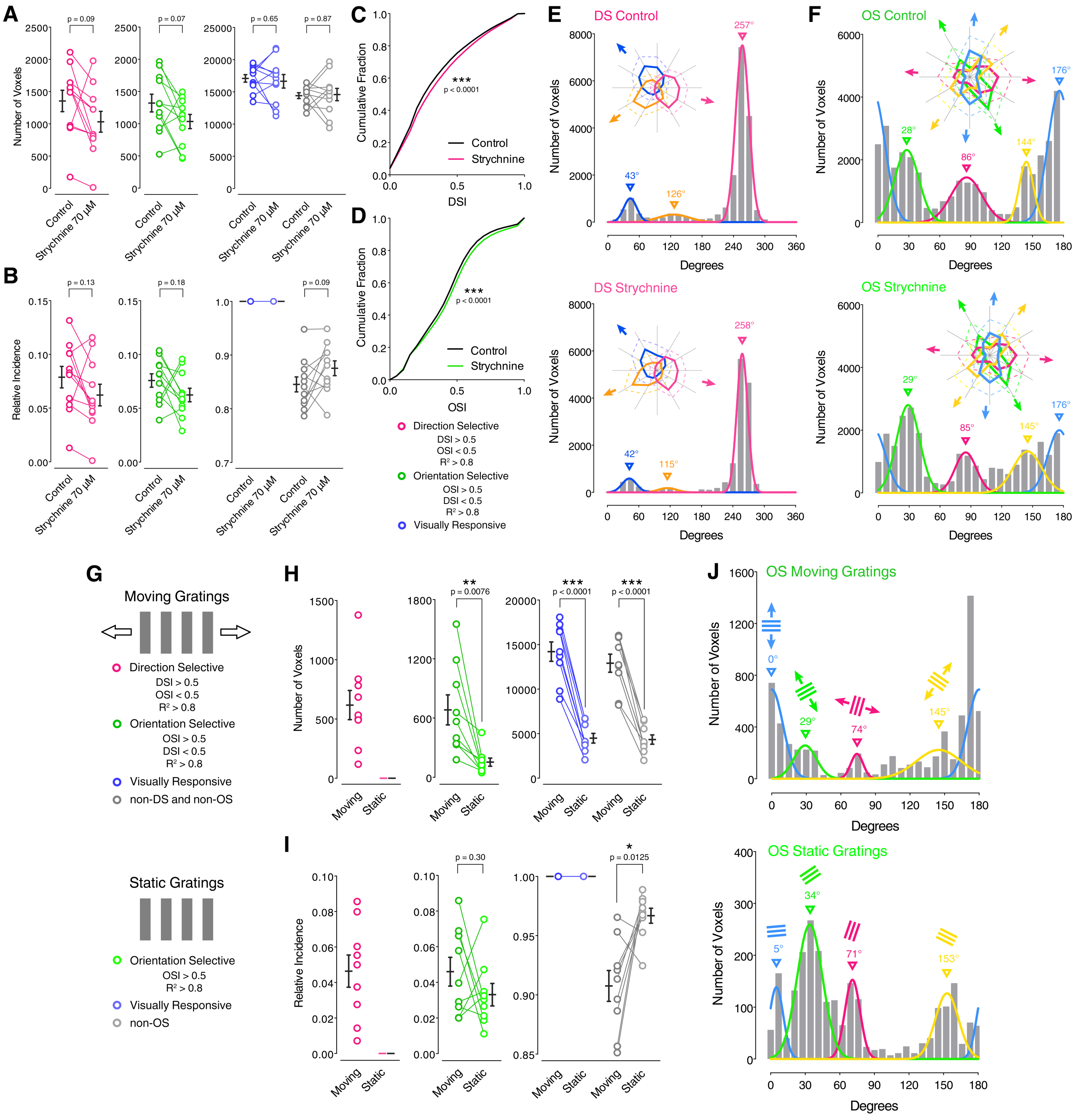

Figure S5. Role of Glycinergic Inhibitory Input in Generating RGC Orientation Selectivity, and RGC Orientation-selective Responses Using Moving or Static Gratings, Related to Figure 4

(A and B) Average number (A) and relative frequency (B) of DS, OS, visually responsive and non-DS/non-OS voxels per Z-plane in 5-dpf *Tg(isl2b:Gal4;UAS:SyGCaMP3)* larvae (n = 11 larvae) before (control) and after (strychnine) the application of strychnine (70 µM). Error bars are ± SEM. Paired two-tailed Student's t test.

(C and D) Cumulative distributions of DSI values ($R^2 > 0$) across voxels with OSI < 0.5 (C) and OSI values ($R^2 > 0$) across voxels with DSI < 0.5 (D) before (control) and after (strychnine) the application of strychnine (70 µM). ***p < 0.001, two-sample Kolmogorov-Smirnov test.

(E and F) Cumulative histograms summarising the incidence of preferred angles for identified DS (E) and OS voxels (F) in 5-dpf larvae (n = 11 larvae) before (control) and after (strychnine) the application of strychnine (70 µM). Overlaid curves are the fitted Gaussian distributions for each DS or OS subtype. Polar plots illustrate the mean (+ 1 SD) normalised response profiles for each DS or OS subtype.

(G) Diagram showing the criteria used to identify DS and OS voxels when using moving (top) or static gratings (bottom). The DSI cannot be calculated when using static stimuli, therefore only OS, visually responsive and non-OS voxels can be identified when larvae are stimulated with static gratings.

(H and I) Average number (H) and relative frequency (I) of DS, OS, visually responsive and non-DS/non-OS RGC voxels per Z-plane in 5-dpf *Tg(isl2b:Gal4;UAS:SyGCaMP3)* larvae (n = 9 larvae) using either moving or static gratings. Individual larvae are tested using both stimuli. Error bars are ± SEM. *p < 0.05, **p < 0.01, ***p < 0.001, paired two-tailed Student's t test.

(J) Cumulative histograms summarising the incidence of preferred angles for identified OS voxels in 5-dpf larvae (n = 9 larvae) using either moving (left) or static gratings (right). Overlaid curves are the fitted Gaussian distributions for each OS subtype, with the colour-coded bars indicating the preferred orientation of gratings. Note that, in both stimulus groups, OS populations with similar preferred angles are present, indicating that OSGCs are tuned to the stimulus orientation not its axis of movement.

Figure S6

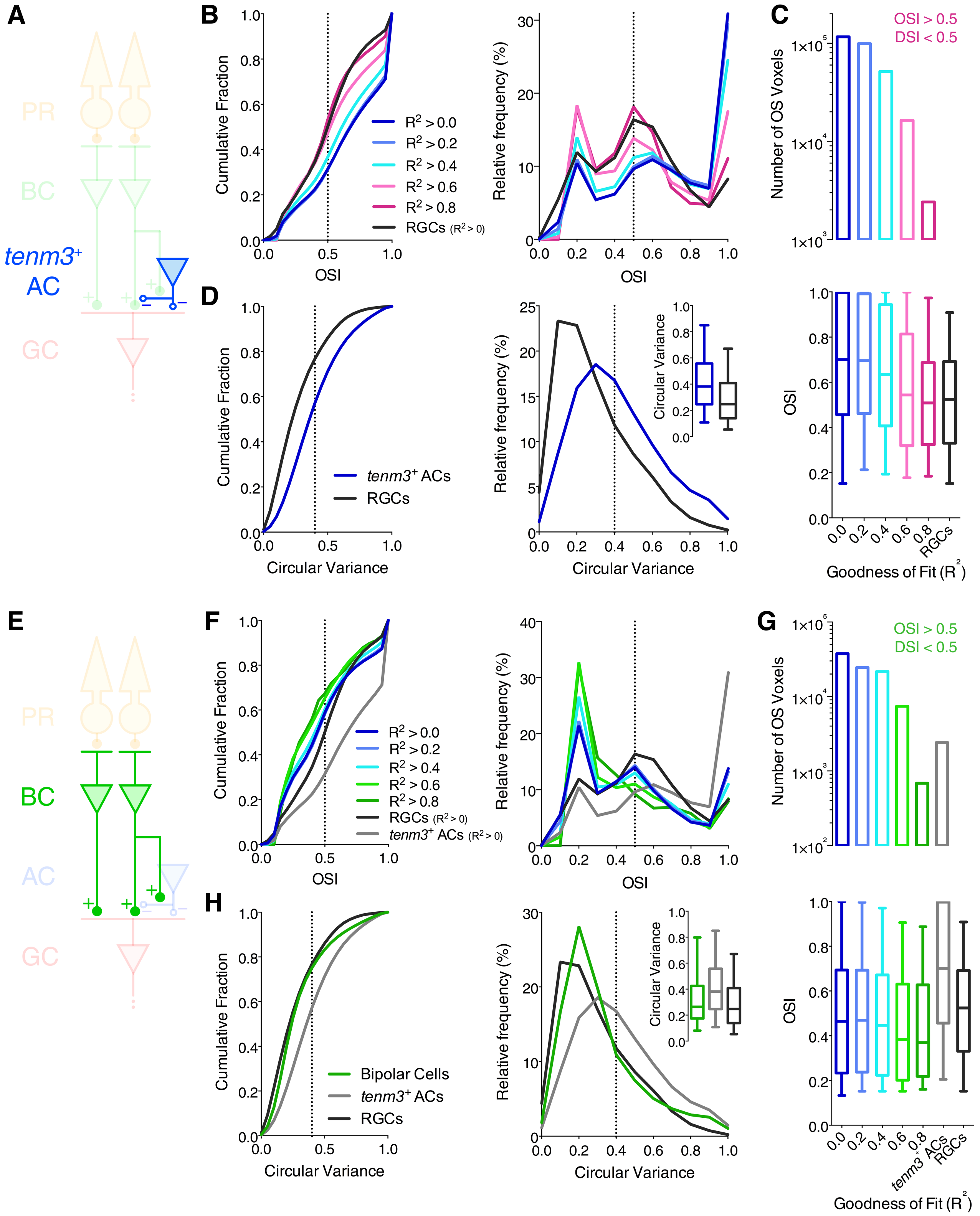

A
PR
BC
tenm3+ AC
GC
B
Cumulative Fraction
OSI
R2 > 0.0
R2 > 0.2
R2 > 0.4
R2 > 0.6
R2 > 0.8
RGCs (R2 > 0)
Relative frequency (%)
C
OSI > 0.5
DSI < 0.5
Number of OS Voxels
D
Circular Variance
tenm3+ ACs
RGCs
OSI
Goodness of Fit (R2)
E
PR
BC
AC
GC
F
tenm3+ ACs (R2 > 0)
G
H
Bipolar Cells
tenm3+ ACs
RGCs

Figure S6. Orientation Selectivity in Tenm3$^+$ ACs and BC Ribbon synapses, Related to Figure 6 and Movies S4 and S5

(A) Selective recording of *tenm3*$^+$ AC synaptic terminals through two-photon calcium imaging in 4-dpf *Tg(tenm3:Gal4;UAS:SyGCaMP3)* larvae.

(B) Cumulative distribution (left) and relative frequency (right) of orientation selectivity index (OSI) values across voxels with DSI < 0.5 in *tenm3*$^+$ ACs (n = 20 larvae) and RGCs (black, n = 23 larvae; data from Figure 1H). The colour code indicates the progressively higher goodness of fit (R^2 of the fitted von-Mises profiles) criteria applied to the data in order to identify voxels characterised by high orientation selectivity. The black dotted lines indicate the OSI threshold used to identify OS responses (OSI > 0.5). Depending on the R^2 value chosen as threshold, the number of voxels that satisfy the criteria, and therefore are represented in the distributions, varies.

(C) Total number of OS voxels (top) and distribution of OSI values (bottom) using progressively higher R^2 values in *tenm3*$^+$ ACs and RGCs (black, $R^2 > 0$). Boxplots indicate interquartile ranges (boxes), medians (lines in boxes) and 10-90 percentiles (whiskers).

(D) Cumulative distribution (left) and relative frequency (right) of circular variance values across all voxels in *tenm3*$^+$ ACs (blue, n = 20 larvae) and RGCs (black, n = 23 larvae). The black dotted lines indicate the circular variance threshold used to identify OS responses (circular variance < 0.4). Boxplots in the right inset indicate interquartile ranges (boxes), medians (lines in boxes) and 5-95 percentiles (whiskers).

(E) Selective recording of BC ribbon synapses through two-photon calcium imaging in 4-dpf *Tg(-1.8ctbp2:SyGCaMP6)* larvae.

(F) Cumulative distribution (left) and relative frequency (right) of orientation selectivity index (OSI) values across voxels with DSI < 0.5 in BCs (n = 20 larvae), *tenm3*$^+$ ACs (grey, n = 20 larvae) and RGCs (black, n = 23 larvae; data from Figure 1H). The colour code indicates the progressively higher goodness of fit (R^2 of the fitted von-Mises profiles) criteria applied to the data in order to identify voxels characterised by high orientation selectivity. The black dotted lines indicate the OSI threshold used to identify OS responses (OSI > 0.5). Depending on the R^2 value chosen as threshold, the number of voxels that satisfy the criteria, and therefore are represented in the distributions, varies.

(G) Total number of OS voxels (top) and distribution of OSI values (bottom) using progressively higher R^2 values in BCs, *tenm3*$^+$ ACs (grey, $R^2 > 0$) and RGCs (black, $R^2 > 0$). Boxplots indicate interquartile ranges (boxes), medians (lines in boxes) and 10-90 percentiles (whiskers).

(H) Cumulative distribution (left) and relative frequency (right) of circular variance values across all voxels in BCs (green, n = 20 larvae), *tenm3*$^+$ ACs (grey, n = 20 larvae) and RGCs (black, n = 23 larvae). The black dotted lines indicate the circular variance threshold used to identify OS responses (circular variance < 0.4). Boxplots in the right inset indicate interquartile ranges (boxes), medians (lines in boxes) and 5-95 percentiles (whiskers).

Figure S7

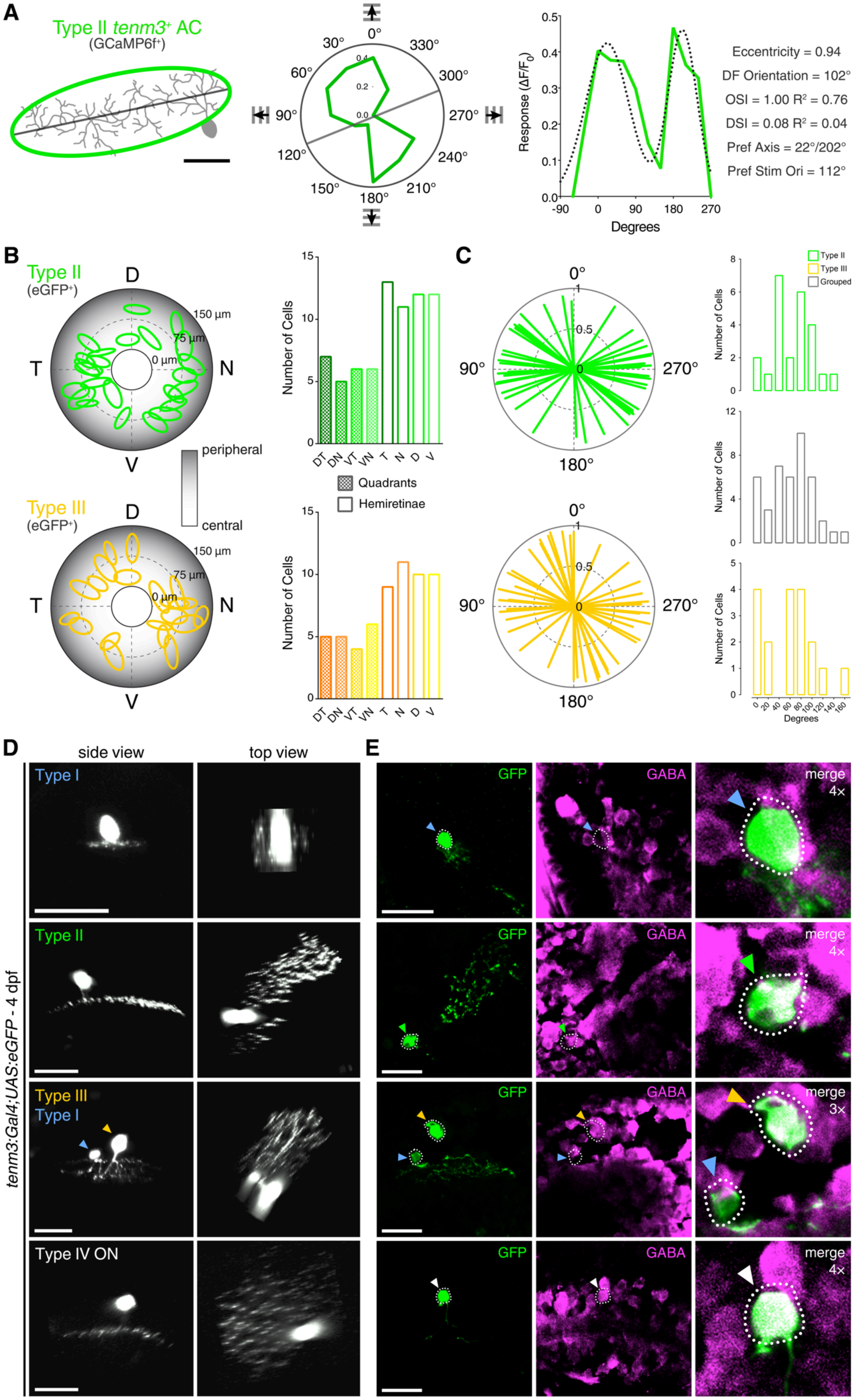

A
Type II tenm3+ AC
(GCaMP6f+)
0°
30°
330°
60°
300°
90°
270°
120°
240°
150°
210°
180°
Response (ΔF/F0)
Degrees
Eccentricity = 0.94
DF Orientation = 102°
OSI = 1.00 R² = 0.76
DSI = 0.08 R² = 0.04
Pref Axis = 22°/202°
Pref Stim Ori = 112°
B
Type II
(eGFP+)
D
T
N
V
150 µm
75 µm
0 µm
peripheral
central
Type III
(eGFP+)
Number of Cells
DT
DN
VT
VN
T
N
D
V
Quadrants
Hemiretinae
C
Type II
Type III
Grouped
Number of Cells
Degrees
D
side view
top view
Type I
Type II
Type III
Type IV ON
tenm3:Gal4;UAS:eGFP - 4 dpf
E
GFP
GABA
merge
4×
3×

Figure S7. Functional Imaging, Dendritic Field Orientation and Neurotransmitter Identity of Single Tenm3$^+$ ACs, Related to Figure 6

(A) Summary of morphological and functional analyses of single GCaMP6f-labelled *tenm3*$^+$ ACs in 4-dpf *UAS:GCaMP6f*-injected *Tg(tenm3:Gal4)* larvae. An example of a type II *tenm3*$^+$ AC that showed stimulus-locked responses and high orientation selectivity is displayed. Left: morphological reconstruction of the cell (grey, top view) with corresponding fitted ellipse profile (green) and dendritic field (DF) orientation (black line). Scale bar is 20 μm. Middle: Polar plot representing the tuning profile of the cell (green, obtained by calculating the integral response to each stimulus) in response to gratings moving in different directions. Grey line indicates preferred stimulus orientation (Pref Stim Ori). Radial axis scale shows $\Delta F/F_0$ of integral calcium responses. Right: Tuning profile (green) with fitted sum of two Gaussians (dotted black line). Measurements of morphological and functional characteristics of the example cell are reported on the right. R^2, goodness of fit of the sum of two Gaussians distribution.

(B) Retinal location mapping of individual eGFP-CAAX-labelled type II (green, top) and type III (yellow, bottom) *tenm3*$^+$ ACs in 4-dpf *UAS:eGFP-CAAX*-injected *Tg(tenm3:Gal4)* larvae (n = 24 and 20 cells in 39 larvae, respectively). Frequencies of type II and III *tenm3*$^+$ ACs in number of cells per retinal quadrant or hemiretina are reported on the right. D, dorsal; V, ventral; N, nasal; T, temporal.

(C) Grouped data summarising dendritic field orientation and elongation (quantified by the eccentricity of dendritic fields; radial axis) of individual eGFP-CAAX-labelled type II (green, top) and type III (yellow, bottom) *tenm3*$^+$ ACs (n = 24 and 20 cells in 39 larvae, respectively). Radial axis scale of the polar plots shows the eccentricity of dendritic fields. Frequencies of type II and III *tenm3*$^+$ ACs in number of cells per orientation (20° bin width; 0°-180° orientation space) are reported on the right.

(D) Mosaically labelled *tenm3*$^+$ ACs expressing eGFP in 4-dpf *UAS:eGFP*-injected *Tg(tenm3:Gal4)* larvae. Both side views (left) and top views (right) are shown. Scale bars are 20 μm.

(E) Immunostaining showing the expression of γ-aminobutyric acid (GABA; magenta) in the identified eGFP-expressing *tenm3*$^+$ ACs (green) reported in (D). Arrowheads are colour-coded according to the *tenm3*$^+$ AC type each cell is classified into. Note that both type II (green arrowheads) and type III (yellow arrowheads) *tenm3*$^+$ ACs express the neurotransmitter GABA. Interestingly, type I *tenm3*$^+$ ACs (cyan arrowheads) do not appear to express GABA. Note that the merged images (right) have been enlarged by the reported magnification factors to help visualisation. Scale bars are 20 μm.

SUPPLEMENTAL EXPERIMENTAL PROCEDURES

Animals

Zebrafish were maintained at 28.5°C on a 14 hr ON/10 hr OFF light cycle in Danieau solution [58 mM NaCl, 0.7 mM KCl, 0.4 mM $MgSO_4$, 0.6 mM $Ca(NO_3)_2$ 5.0 mM HEPES, pH 7.6]. Transgenic lines used in this study include *Tg(isl2b:Gal4)* [S2], *Tg(UAS:SyGCaMP3)* [S3], *Tg(UAS:tagRFP-CAAX)* [S4], *Tg(UAS:KillerRed)* [S5], *Tg(elavl3:GCaMP5G)* [S6], *Tg(UAS:GCaMP5)* (gift from Dr. Elena Dreosti, Department of Cell and Developmental Biology, University College London, London, UK), and *Tg(-1.8ctbp2:SyGCaMP6)* [S7]. The *Tg(tenm3:Gal4)* zebrafish line was generated through BAC transgenic insertion (*kg302Tg*) by the authors (see *BAC Transgenesis*). The $tenm3^{KO}$ mutant line was generated through transcription activator-like effector nuclease (TALEN)-mediated genome editing ($tenm3^{kg69/kg69}$) from Ekkwill zebrafish by the authors (see *TALEN-mediated tenm3 Knock-out*). WT retinae used in Figures S1C-S1F and S2B were obtained from Ekkwill larvae.

Confocal functional imaging experiments were performed in the pigmentation mutant, *nacre*, which lacks all neural crest-derived melanophores [S8]. Two-photon functional imaging experiments in the retina were performed in a triple pigmentation mutant ($alb^{b4/b4}$;$nacre^{w2/w2}$;$roy^{a9/a9}$) which is characterized by a general decrease in melanin synthesis ($alb^{b4/b4}$) [S9] and lacks all neural crest-derived melanophores ($nacre^{w2/w2}$) [S8] and iridophores ($roy^{a9/a9}$) [S10]. The rationale behind this strategy was to have an unobstructed optical access of the retina without raising fish in 200 µM phenylthiourea (PTU) [S11], which has been shown to impair both the normal development of zebrafish [S12, S13] and the retinal responses to light stimuli [S14]. Importantly, $alb^{b4/b4}$;$nacre^{w2/w2}$;$roy^{a9/a9}$ larvae and adult zebrafish are viable and do not display evident morphological, functional or behavioural abnormalities. Larvae used for *in vivo* and *ex vivo* imaging of morphological and molecular features (not involving functional experiments) were raised in 200 µM PTU (Sigma) in Danieau solution to avoid pigment formation. This work was approved by the local Animal Care and Use Committee (King's College London), and was carried out in accordance with the Animals (Experimental Procedures) Act, 1986, under license from the United Kingdom Home Office.

Genome Editing

TALEN-mediated tenm3 Knock-out

Zebrafish *tenm*3 knock-out mutants ($tenm3^{KO}$) were generated by TALEN-mediated genome editing. The exon encoding the transmembrane domain of the Tenm3 protein was targeted. The DNA sequences targeted by the TALEN constructs are the following (5'>3'): left, TCAAAGTACTGTTCATGG; right, GTGCCATGGCCGTCTCCA. TALEN constructs were designed using ZiFiT targeter version 4.2 [S15], and assembled following the REAL assembly protocol [S16]. The T7 transcription mMessage mMachine kit (Life Technologies) was used to produce the 5' capped TALEN mRNA, 200 pg of which was injected into Ekkwill zebrafish embryos at one-cell stage [S17]. Founders were identified using the Surveyor nuclease mismatch assay

(Transgenomic) and outcrossed to generate heterozygous F1 offspring, which were subsequently fin-clipped and genotyped by PCR amplifying and sequencing a 400 bp amplicon using primers that flanked the target site. The primers used for genotyping are the following (5'>3'): tenm3_fw CAGGCACTTTCTCTTTAAGACAGG; tenm3_rev CTCACGTGATCCTCTCTCAGC. Zebrafish were selected where we detected indels leading to frame shifts and consequent premature stop codons causing truncations in the transmembrane domain of Tenm3. The *tenm3*^{KO} mutant zebrafish line (*tenm3*$^{kg69/kg69}$) used for all subsequent experiments is characterized by a 14-bp deletion (5'-ACAGCCCTCAGTGC-3') in the following genomic location of *tenm3*: chromosome 1, 38,921,612-38,921,625.

BAC Transgenesis

The *Tg(tenm3:Gal4)* transgenic zebrafish line was generated through bacterial artificial chromosome (BAC) transgenic insertion (*kg302Tg*) following previously described methods [S18]. In summary, the recombineering procedure used to generate *tenm3:Gal4* BAC constructs consists of three steps. First, three different BAC clones (CH211-58M24, 200 kb; CH211-130I22, 155 kb; CH211-169K10, 150 kb; BACPAC Resources, CHORI) containing partially overlapping regions of the *tenm3* genomic sequence were transformed with the *pRedFlp4* plasmid. Second, the *iTol2* cassette with 50-bp homologies on each end (*iTol2-amp*) was inserted into *tenm3* BAC vector backbones. Third, the transcriptional activator Gal4FF gene together with a kanamycin resistance cassette possessing 50-bp homology arms (*Gal4FF-kan*) was inserted at the start ATG site of the *tenm3* gene (the exon containing the start ATG codon is in chromosome 1: 38,879,739-38,879,945).

To create first transient and subsequently stable transgenic zebrafish lines, isolated BAC DNA constructs (120 ng/µl) were co-injected with *Tol2* transposase mRNA (100 ng/µl) into *Tg(UAS:GCaMP5)* zebrafish embryos at the early one-cell stage. Transient expression, visible as GCaMP5 fluorescence, already gave a reliable indication of the final expression in the stable transgenic line. All three BAC constructs produced analogous transient expression patterns. The CH211-58M24 (200 kb) BAC construct was used to generate the *Tg(tenm3:Gal4)* zebrafish line because of its higher level of Gal4FF expression compared to the other two BAC constructs. The CH211-58M24 BAC construct contains genomic regions 100 kb upstream and 100 kb downstream of the *tenm3* start codon site, but does not cover the entire *tenm3* genomic locus (~400 kb). The BAC DNA preparation was obtained using the HiPure Midiprep kit (Invitrogen), with modifications for BAC DNA isolation as described by the manufacturer. *tol2* transposase mRNA was prepared by *in vitro* transcription from *Xba*I-linearized *pDB600* plasmid [S19] using the T3 transcription mMessage mMachine kit (Life Technologies). RNA was purified using the RNeasy MinElute Cleanup kit (Qiagen). Germ line transmission was identified by mating sexually mature adult fish to wild-type fish and, subsequently, examining their progeny for GCaMP5 fluorescence. Positive embryos were raised to adulthood and outcrossed to generate the *Tg(tenm3:Gal4)* single transgenic line, which was then used as driver line.

This is the list of primers used for recombineering (5’>3’).

**itol2 cassette amplification**	
ptarBac_itol2_fw	GCGTAAGCGGGGCACATTTCATTACCTCTTTCTCCGCACCCGACATAGATCCCTGCTCGAGCCGGGCCCAAGTG
ptarBac_itol2_rev	CGCGGGGCATGACTATTGGCGCGCCGGATCGATCCTTAATTAAGTCTACTAATTATGATCCTCTAGATCAGATCT
**Gal4FF-Kan cassette amplification**	
tenm3BAC_Gal4FF_fw	ATTTTATTTTCTTCCTTTGGTTTAAAGTGATTTTTCTTTCTATAGGTTCTACCATGAAGCTACTGTCTTCTATCGAAC
tenm3BAC_KanR_rev	GGTTGAGAATGGGAATGCTCAGTGACTGATGGACTGGAGAGAGAGGATGGTCAGAAGAACTCGTCAAGAAGGCG
**itol2 cassette insertion control**	
pTarBAC_HA1_control_fw	CTGTCAAACATGAGAATTGGTC
amp_HA1_control_rev	ACATTTCCCCGAAAAGTGG
amp_HA2_control_fw	CTGAGATAGGTGCCTCACTG
pTarBAC_HA2_control_rev	GAGAGCCTTCAACCCAGTC
**Gal4FF-Kan cassette insertion control**	
tenm3BAC_control_fw	GCTACTCACCATTGCTGTGGCC
Gal4FF_HA1_control_rev	AGTAGCGACACTCCCAGTTG
kanR_HA2_control_fw	TCCTCGTGCTTTACGGTATC
tenm3BAC_control_rev	CGCCTGTGTGGTGGCATTAC

Imaging

Confocal Imaging

Imaging was performed using an LSM 710 confocal microscope equipped with a spectral detection scan head and a 20×/1.0 NA water-immersion objective (Carl Zeiss). Optical sections were obtained at 1 µm intervals through the Z axis. Maximum intensity projections and 3D rotated images were generated using either ImageJ [S20] or ZEN (Carl Zeiss).

In Vivo Calcium Imaging

Confocal

Functional time-series of visually evoked calcium responses in RGCs were acquired at a rate of 4.1 Hz and 0.415×0.415 µm resolution (256×256 pixels) and 1 AU pinhole aperture, using the confocal setup described above. The average diameter of a presynaptic bouton in zebrafish RGCs is ~0.8 µm. Thus, the physical X-Y dimensions of voxels are below that of a typical presynaptic bouton. Excitation was provided by 488 nm multi-line laser. Non-anaesthetised *Tg(isl2b:Gal4; UAS:SyGCaMP3)*, *Tg(tenm3:Gal4;UAS:KillerRed;elavl3:GCaMP5G)* and *UAS:GCaMP6f*-injected *Tg(isl2b:Gal4)* larvae were immobilized in 2% low melting point agarose (Sigma) prepared in Danieau solution and mounted dorsal side up on a raised glass platform that was placed in a custom-made Danieau-filled chamber. The agarose was sufficient to restrain the larvae so that anaesthesia was not required. Imaging was performed in the afternoon (1-8 pm).

Two-photon

Two-photon functional imaging in the retina was performed using an A1R MP microscope equipped with a 4-channel GaAsP NDD and an Apochromat 25×/1.1 NA water-immersion objective (Nikon). Excitation was provided by a Chameleon Ultra II Mode-locked titanium-sapphire laser (Coherent) tuned to 930 nm. Time-series of visually evoked calcium responses in *tenm3*$^+$ ACs and BCs were acquired at a rate of 7.8 Hz and 0.397×0.397 μm resolution (256×128 pixels). Following activation of the laser scanning, we waited 60 seconds before starting the visual stimulation to ensure the retina adapted to the background light level caused by the multi-photon laser. 4-dpf *Tg(tenm3:Gal4;UAS:SyGCaMP3)*, *Tg(-1.8ctbp2:SyGCaMP6)* and *UAS:GCaMP6f*-injected *Tg(tenm3:Gal4)* larvae were first paralysed for 10-15 minutes in α-bungarotoxin (1 mg/ml; Biotium) prepared in Danieau solution. Subsequently, larvae were immobilized in 2% low melting point agarose (Sigma) and mounted on a raised custom-made glass platform with the dorsal side up (45° angle) and one eye facing an LCD screen (see Visual Stimulation) that was placed underneath a custom-made Danieau-filled chamber. Imaging was performed in the afternoon (1-8 pm).

Visual Stimulation

Moving Bars and Moving vs. Static Gratings in Confocal Preparation

Moving bar stimuli were generated as previously described [S1, S21]. A diffusion filter (3026, Rosco) was bonded to one side of the chamber to serve as a projection screen. The agarose in front of the eye facing the projection screen was removed, allowing an unobstructed view of the projected image on the side of the chamber. Larvae were positioned 3 cm away from the screen, and the projected image filled a visual field of ~97° x 63°. Visual stimuli consisted of light (56 cd/m^2) or dark bars (8 cd/m^2) (175% and 25% of mean luminance, respectively) on a mean gray background (32 cd/m^2). As no qualitative differences between light and dark bars were noted, data obtained using the two stimuli were combined. Each bar was 10° in width moving at a speed of 20°/s and separated from the preceding bar by 30°, enabling more than one bar on the screen at any one time. The long axes of the bars were orthogonal to the direction of motion. For the moving vs. static gratings stimuli used to obtain data displayed in Figures S5G-S5J, either moving square-wave gratings (75% contrast, spatial frequency 0.05 cycles/degree equivalent to 10° bar width, temporal frequency 1 cycles/s) or static square-wave gratings with alternating contrast (75% contrast, spatial frequency 0.05 cycles/degree equivalent to 10° bar width) were used. Each of the 12 directions of motion was presented once (3 seconds) in a pseudo-random order unique to each slice in every animal imaged. Each inter-epoch interval was 10 seconds to enable SyGCaMP3 or GCaMP6f signals to return to baseline. A blank-screen null condition of 2 seconds was also interleaved. Visual experiments were generated and controlled using custom-written Labview and MATLAB code (MathWorks), implemented on a ViSaGe stimulus presenter (Cambridge Research Systems) and delivered via a DLP pico projector (Optoma).

Moving Gratings in Two-photon Preparation

Moving gratings stimuli in the two-photon preparation were generated and controlled using PsychoPy [S22], and delivered through an LCD screen (SKD5VA-3, GoodWell Technology) positioned underneath a custom-made Perspex chamber. A long-pass red glass filter (FGL610, Thorlabs) was positioned between the LCD screen and the chamber to allow for simultaneous imaging and visual stimulation. Larvae were positioned 2 cm away from the screen and the image on the LCD screen filled a visual field of ~140° x 100° (mean background luminance 30.4 cd/m^2). Visual stimuli consisted of square-wave gratings (100% contrast, spatial frequency 1.66 cycles/cm, temporal frequency 1 cycles/s). Each grating bar was 8.5° in width and the long axes of the bars were orthogonal to the direction of motion. Each of the 12 directions of motion was presented once (6 seconds) with and inter-epoch interval of 10 seconds to enable SyGCaMP3 or SyGCaMP6 signals to return to baseline. A blank-screen null condition of 6 seconds was also interleaved. TTL triggers (0-5-0 Volts) to record epoch time events where generated through a LabJack USB DAQ device (U3-LV, LabJack Corporation). Following activation of the laser scanning, we waited 60 seconds before starting the visual stimulation to ensure the retina adapted to the background light level caused by the multi-photon laser.

Single Cell Labelling

Labelling of Individual RGCs

To label individual RGCs, *UAS:GCaMP6f* DNA constructs [S23, S24] were injected into 1-4 cell-stage *Tg(isl2b:Gal4)* embryos. The *pGP-CMV-GCaMP6f* plasmid used to clone the *UAS:GCaMP6f* DNA construct [S24] was a gift from Douglas Kim (Addgene plasmid # 40755). Plasmid DNA was prepared using midiprep kits (Qiagen) and injected at a concentration of 30 ng/µl in Danieau solution. Only larvae showing single GCaMP6f-expressing RGC axons in the optic tectum were used for functional analyses. After functional identification of orientation-selective RGCs (OSGCs), larvae were fixed, cryosectioned and immunostained (see *Immunohistochemistry*) to visualize the morphology of GCaMP6f$^+$ dendritic arbours of OSGCs.

Mosaic Labelling of Tenm3$^+$ ACs

To mosaically label *tenm3*$^+$ ACs, *UAS:eGFP-CAAX* (gift from Dr. Andy Symonds, MRC Centre for Developmental Neurobiology, King's College London, London, UK), *UAS:eGFP* [S2] or *UAS:GCaMP6f* [S24] DNA constructs were injected into 1-4 cell-stage *Tg(tenm3:Gal4)* embryos. Plasmid DNA was prepared using midiprep kits (Qiagen) and injected at a concentration of 40-50 ng/µl in Danieau solution.

Optogenetic Ablation *of Tenm3$^+$ ACs*

The optogenetic ablation of *tenm3*$^+$ ACs was performed using the genetically encoded photosensitizer KillerRed [S5, S25] following previously described guidelines [S26, S27]. At 2 dpf, the eyes of KillerRed$^+$ *Tg(tenm3:Gal4;UAS:KillerRed;elavl3:GCaMP5G)* and *Tg(tenm3:Gal4;*

UAS:KillerRed) larvae, and KillerRed$^-$ *Tg(tenm3:Gal4;elavl3:GCaMP5G)* and *Tg(tenm3:Gal4)* control larvae were illuminated with continuous intense green light (540-552 nm; filter set 15, BP 546/12 nm) for 40 minutes using a wide-field fluorescence microscope (Zeiss Axioskop) equipped with a 40× objective and a 100 Watts mercury lamp employed at maximal light intensity and objective aperture. In KillerRed$^+$ larvae, strong photobleaching (~90% decrease in relative fluorescence) was observed after green light illumination, indicating effective KillerRed phototoxicity. To detect cell death, 3-4 hours following illumination the larvae were fixed in 4% paraformaldehyde (PFA) at 4°C overnight. Subsequently, larvae were cryosectioned and fluorometric terminal deoxynucleotidyl transferase dUTP nick end labeling (TUNEL; DeadEnd, Promega) assays were performed on sections following standard protocols.

Pharmacology

Block of Glutamate Receptors in the Optic Tectum

To isolate RGC axonal calcium responses from tectal cell dendritic responses in 4-dpf *Tg(tenm3:Gal4;UAS:KillerRed;elavl3:GCaMP5G)* and *Tg(tenm3:Gal4;elavl3:GCaMP5G)* larvae, we pressure-injected the NMDA and AMPA/kainate glutamate receptor antagonists D-2-amino-5-phosphonovaleric acid (APV; Sigma) and 2,3-dihydroxy-6-nitro-7-sulfamoyl-benzo[f]quinoxaline-2,3-dione (NBQX; Sigma), 100 μM and 20 μM in Danieau solution respectively, into one tectal hemisphere as previously described [S4]. Larvae were imaged immediately post-injection. Animals were only included in the analyses if visual responses were not detected in tectal cell bodies following pharmacological treatment.

GABA$_A$ Receptors Block

To block γ-aminobutyric acid (GABA)-mediated inhibition from amacrine cells, we used the GABA$_A$ receptor chloride channel blocker picrotoxin (Sigma) at a final concentration of 100 μM, as previously described [S28]. Functional calcium imaging was first performed in 4-dpf *Tg(isl2b:Gal4;UAS:SyGCaMP3)* larvae in Danieau solution with no drug. Subsequently, larvae were mounted in 2% low melting point agarose (Sigma) in Danieau solution with freshly made 100 μM picrotoxin and then imaged again. Importantly, also the Danieau solution in the imaging chamber contained 100 μM picrotoxin.

Glycine Receptors Block

To block glycine-mediated inhibition from amacrine cells, we used the glycine receptor antagonist strychnine (Sigma) at a final concentration of 70 μM [S29]. Functional calcium imaging was first performed in 5-dpf *Tg(isl2b:Gal4;UAS:SyGCaMP3)* larvae in Danieau solution with no drug. Subsequently, larvae were mounted in 2% low melting point agarose (Sigma) in Danieau solution with freshly made 70 μM strychnine and then imaged again. Importantly, also the Danieau solution in the imaging chamber contained 70 μM strychnine.

Immunohistochemistry

The primary antibodies used in the study were diluted in blocking solution as follows: chicken anti-GFP (1:500; Abcam, AB13970), rabbit anti-GABA (1:100; Sigma, A2052), guinea pig anti-GABA (1:500; Abcam, AB17413), mouse anti-Parvalbumin (1:300; Millipore, MAB1572), rabbit anti-TH (1:500; Millipore, AB152), goat anti-ChAT (1:100; Millipore, AB144P), rabbit anti-glycine (1:50; Abcam, AB9442). The primary antibody used to detect the Tenm3 protein in Figures S1C-S1F is a custom-made rabbit anti-tenm3 antibody (1:150 dilution in blocking solution; Eurogentec) designed to recognise the following protein sequence, 445-TIDTGEVDVGRRAVQ-459, which is located in the extracellular domain of the zebrafish Tenm3 protein. The secondary antibodies used in the study were goat or donkey anti-chicken, anti-mouse, anti-rabbit, anti-guinea pig and anti-goat conjugated to Alexa Fluor 488 or 568 fluorophores (1:1000 dilution in blocking solution; Life Technologies). Nuclei were counterstained with TO-PRO-3 Iodide (1:1000 dilution in blocking solution; Life Technologies, T3605).

Immunostainings of zebrafish larvae on cryosections were performed following standard procedures. Briefly, larvae were fixed in 4% PFA overnight, rehydrated with PBS for 3×10 minutes, cryoprotected in 15%, 30% sucrose at room temperature for 2 hours and, finally, 40% sucrose at 4°C overnight. Larvae used to immunostain Tenm3 in the retina were subjected to the following antigen retrieval procedure [S30]: the cryoprotected larvae were incubated in 150 mM Tris-HCl pH 9.0 at room temperature for 5 minutes, followed by heating at 70°C for 15 minutes and, finally, re-cryoprotected in 40% sucrose at 4°C overnight. The cryoprotected larvae were then embedded in molds using O.C.T. compound (VWR International) and cryosectioned (18 µm thickness). Cryosections were mounted on glass slides (Superfrost plus, Thermo Scientific) and dried overnight at room temperature. Subsequently, slides were equilibrated 3×5 minutes in PBST (0.1% Tween-20 in PBS) and then blocked with 3% goat or donkey serum + 1% BSA in PBST for 1 hour at room temperature. After blocking, the slides were incubated with the respective primary antibodies at 4°C overnight. Finally, slides were washed 3×30 minutes in PBST, incubated with the respective secondary antibodies at room temperature for 2-3 hours, and then washed 3×30 minutes in PBST. To preserve the immunostained cryosections, Fluoromount-G (eBioscience) was used. Slides were then covered with glass coverslips and stored at 4°C until use.

Analysis

Functional Analyses

In vivo calcium imaging data were analysed as previously described [S1, S21]. In summary, functional time-series were processed before analysis as follows: time-series images from each experiment were corrected for motion with a rigid-body algorithm (SPM12; http://www.fil.ion.ucl.ac.uk/spm/), median filtered with a kernel size of 1 voxel to remove dark and shot noise, and spatially smoothed with a 2D Gaussian kernel = 2 voxels to improve signal-to-noise. A baseline (B) that corrects for low-frequency drifts was determined using a cubic-spline algorithm extrapolating

between knots averaged from 5 s of the inter-epoch interval data. Both relative signal intensity changes (ΔF = F-B; where F = raw fluorescence) and normalised signal intensity changes [$\%\Delta F/F_0$ = (F-B)/B] were calculated at each voxel. ΔF was used for population functional data (voxel-wise analysis), whereas $\%\Delta F/F_0$ was used for single cell functional data (manually defined regions of interest, ROIs, of dendritic or axonal arbours of GCaMP6f-labelled cells). For each voxel or ROI the integral response over the epoch-interval was calculated to provide a single response metric of each presented direction of stimulus motion. The integral within each epoch window is a summary metric more resistant to saturation effects of the calcium probe than maximum signal change. A threshold for each voxel within an acquisition image sequence was determined from the variance of ΔF changes during the inter-epoch intervals and null condition, threshold = 5×SDs. All voxels that were supra-threshold within at least two visual presentation epochs were regarded as visually responsive and subjected to further characterization.

To analyse the direction and orientation selectivity of visually responsive voxels or ROIs, direction- and orientation-selective indices (DSI and OSI) [S31], based on fitted von-Mises or Gaussian profiles [S32], were calculated together with an estimate for their goodness of fit, R^2. The DSI was defined as $(R_{pref}-R_{null})/(R_{pref}+R_{null})$, where R_{pref}, the response to the preferred direction, was the integral response over the preferred direction epoch-interval. R_{null} was similarly calculated as the integral response evoked by the direction opposite to the preferred direction (180° angular distance). The OSI was defined as $(R_{pref}-R_{orth})/(R_{pref}+R_{orth})$, where R_{pref}, the response to the preferred orientation, was the integral response over the preferred orientation epoch-interval. R_{orth} was similarly calculated as the integral response evoked by the orientation orthogonal to the preferred orientation (90° angular distance). To minimize cross talk and over-fitting associated with DSI and OSI metrics, a stringent approach was undertaken. For a voxel or ROI to be regarded as direction-selective (DS) or orientation-selective (OS), mutually exclusive criteria were used: DS if DSI > 0.5 and OSI < 0.5; and OS if OSI > 0.5 and DSI < 0.5. In both cases, the goodness of fit (R^2) for DSI and OSI, respectively, had to be > 0.8; thus, the fitted curves explained at least 80% of the integral responses. A single von Mises distribution was used to fit responses of DS voxels and estimate their preferred direction of motion angle from the centre of the fitted curve, respectively. The sum of two von Mises or Gaussian distributions (180° angular distance apart) was used to fit responses of OS voxels or OS ROIs, respectively, and estimate their preferred orientation of motion angles from the centres of the fitted curves. Grouped cumulative histograms of the preferred direction or orientation of motion angles were generated from the calculated preferred angles of identified DS or OS voxel. The distribution of preferred angles for each identified DS or OS subpopulation of voxels was fitted by a Gaussian distribution with peak centre (mean), height (amplitude) and standard deviation being free dimensions. Circular variance was also calculated for comparison as an alternative metric of orientation selectivity (Circular variance < 0.4) [S33].

Morphological Analyses

To determine the IPL stratification profile of *tenm3*$^+$ AC neurites and OSGC dendrites, rectangular 10 µm-wide ROIs were drawn across the IPL. The Plot Profile function in ImageJ [S20] was applied to the ROIs to calculate fluorescence intensity traces across the IPL depth. The traces obtained from multiple larvae were then normalised and averaged using SigmaPlot (Systat Software). The dendritic field area and orientation angle of individual eGFP-CAAX- or GCaMP6f-labelled *tenm3*$^+$ ACs were measured using ImageJ [S20] in 3D rotated images generated through ZEN (Carl Zeiss). To quantify the dendritic field elongation of individual *tenm3*$^+$ ACs, we calculated the eccentricity [$e = \sqrt{(1-b^2/a^2)}$; a = length of the ellipse semi-major axis, b = length of the ellipse semi-minor axis] of dendritic arbour profiles following ellipse fitting in ImageJ [S20]. 3D morphological reconstructions of individual *tenm3*$^+$ ACs were obtained using the Simple Neurite Tracer plugin in FIJI [S34]. To estimate the total number of type I-IV *tenm3*$^+$ ACs required to tile the retina with a coverage factor of 1, the mean IPL area (49,903 ± 3,707 µm^2, mean ± SD; n = 14 retinae from 14 larvae at 4 dpf) was divided by the observed mean dendritic field area of each *tenm3*$^+$ AC type and, subsequently, the obtained values were used to estimate the respective relative frequency.

Statistical Analyses

Statistical analyses and tests were carried out using Prism 6 (GraphPad), SigmaPlot 11 (Systat Software) or MATLAB R2014b (MathWorks). A comprehensive description of the statistical analyses and tests performed in this study can be found in Table S1. Before performing statistical tests, descriptive statistics (e.g., normality tests to see whether values come from a Gaussian distribution or F-test to compare variances) were used to choose the appropriate statistical test (reported in Table S1). The criterion for statistical significance was set at $p < 0.05$. In order to quantitatively measure and assess the effects of treatments or genetic manipulations between animal groups, the effect size (Cohen's *d*) and its 95% confidence interval were also calculated (see Table S1). No statistical method was used to predetermine sample sizes, but our sample sizes are similar to those generally employed in the field and in most cases provided a high statistical power (i.e., power > 0.8).

Simulation of OSGC Output

To simulate the tuning profile of orientation-selective ganglion cells (OSGCs) we used the following algorithm:

$$O_{hOSGC} = \sum_{i=m}^{n} (\beta_i + w\alpha_i)$$

The tuning profile of OSGCs tuned to horizontal bars moving along the vertical axis (0°; hence, O_{hOSGC}) was simulated by summating normalised bipolar cell (BC) excitatory input (β) and OS *tenm3*$^+$ amacrine cell (AC) inhibitory input (α) for each stimulus orientation (i) across the whole orientation space. The orientation space ranges from 'm' to 'n', which are negative (-90°) and

positive (90°) angles orthogonal to the preferred orientation (0°), respectively. Values used for 'α' are the experimentally observed average responses of orthogonal orientation-tuned *tenm3*$^+$ ACs (i.e., *tenm3*$^+$ ACs tuned to vertical bars moving along the horizontal axis; OSI > 0.5, DSI < 0.5, R^2 > 0.8; n = 20 larvae). Note that, since the OS *tenm3*$^+$ AC input is inhibitory, a negative synaptic weight factor (w) is used in the algorithm. As a consequence, the OS inhibitory input provided by *tenm3*$^+$ ACs has a subtractive effect on OSGC output. Values used for 'β' are the experimentally observed average responses of BC terminals (n = 20 larvae). Three different orientation-tuning levels of excitatory BC input were tested, namely: a) untuned BC input (average responses of all visually responsive BC voxels), (b) weakly OS (OSI > 0, DSI < 0.5, R^2 > 0) preferred orientation-tuned BC input; c) highly OS (OSI > 0.5, DSI < 0.5, R^2 > 0.8) preferred orientation-tuned BC input. The experimentally observed average response profile of OSGCs tuned to horizontal bars moving along the vertical axis (n = 23 larvae) was used as a comparison to evaluate the accuracy of the simulated OSGC response profile.

SUPPLEMENTAL REFERENCES

S1. Antinucci, P., Nikolaou, N., Meyer, M.P., and Hindges, R. (2013). Teneurin-3 specifies morphological and functional connectivity of retinal ganglion cells in the vertebrate visual system. Cell Rep *5*, 582-592.

S2. Ben Fredj, N., Hammond, S., Otsuna, H., Chien, C.B., Burrone, J., and Meyer, M.P. (2010). Synaptic activity and activity-dependent competition regulates axon arbor maturation, growth arrest, and territory in the retinotectal projection. J Neurosci *30*, 10939-10951.

S3. Nikolaou, N., Lowe, A.S., Walker, A.S., Abbas, F., Hunter, P.R., Thompson, I.D., and Meyer, M.P. (2012). Parametric functional maps of visual inputs to the tectum. Neuron *76*, 317-324.

S4. Hunter, P.R., Lowe, A.S., Thompson, I.D., and Meyer, M.P. (2013). Emergent properties of the optic tectum revealed by population analysis of direction and orientation selectivity. J Neurosci *33*, 13940-13945.

S5. Del Bene, F., Wyart, C., Robles, E., Tran, A., Looger, L., Scott, E.K., Isacoff, E.Y., and Baier, H. (2010). Filtering of visual information in the tectum by an identified neural circuit. Science *330*, 669-673.

S6. Ahrens, M.B., Orger, M.B., Robson, D.N., Li, J.M., and Keller, P.J. (2013). Whole-brain functional imaging at cellular resolution using light-sheet microscopy. Nat Methods *10*, 413-420.

S7. Johnston, J., Ding, H., Seibel, S.H., Esposti, F., and Lagnado, L. (2014). Rapid mapping of visual receptive fields by filtered back projection: application to multi-neuronal electrophysiology and imaging. J Physiol *592*, 4839-4854.

S8. Lister, J.A., Robertson, C.P., Lepage, T., Johnson, S.L., and Raible, D.W. (1999). nacre encodes a zebrafish microphthalmia-related protein that regulates neural-crest-derived pigment cell fate. Development *126*, 3757-3767.

S9. Streisinger, G., Singer, F., Walker, C., Knauber, D., and Dower, N. (1986). Segregation analyses and gene-centromere distances in zebrafish. Genetics *112*, 311-319.

S10. White, R.M., Sessa, A., Burke, C., Bowman, T., LeBlanc, J., Ceol, C., Bourque, C., Dovey, M., Goessling, W., Burns, C.E., et al. (2008). Transparent adult zebrafish as a tool for in vivo transplantation analysis. Cell Stem Cell *2*, 183-189.

S11. Karlsson, J., von Hofsten, J., and Olsson, P.E. (2001). Generating transparent zebrafish: a refined method to improve detection of gene expression during embryonic development. Mar Biotechnol (NY) *3*, 522-527.

S12. Elsalini, O.A., and Rohr, K.B. (2003). Phenylthiourea disrupts thyroid function in developing zebrafish. Dev Genes Evol *212*, 593-598.

S13. Li, Z., Ptak, D., Zhang, L., Walls, E.K., Zhong, W., and Leung, Y.F. (2012). Phenylthiourea specifically reduces zebrafish eye size. PLoS One *7*, e40132.

S14. Page-McCaw, P.S., Chung, S.C., Muto, A., Roeser, T., Staub, W., Finger-Baier, K.C., Korenbrot, J.I., and Baier, H. (2004). Retinal network adaptation to bright light requires tyrosinase. Nat Neurosci *7*, 1329-1336.

S15. Sander, J.D., Maeder, M.L., Reyon, D., Voytas, D.F., Joung, J.K., and Dobbs, D. (2010). ZiFiT (Zinc Finger Targeter): an updated zinc finger engineering tool. Nucleic Acids Res *38*, W462-468.

S16. Reyon, D., Khayter, C., Regan, M.R., Joung, J.K., and Sander, J.D. (2012). Engineering designer transcription activator-like effector nucleases (TALENs) by REAL or REAL-Fast assembly. Curr Protoc Mol Biol *Chapter 12*, Unit 12.15.

S17. Sander, J.D., Cade, L., Khayter, C., Reyon, D., Peterson, R.T., Joung, J.K., and Yeh, J.R. (2011). Targeted gene disruption in somatic zebrafish cells using engineered TALENs. Nat Biotechnol *29*, 697-698.

S18. Bussmann, J., and Schulte-Merker, S. (2011). Rapid BAC selection for tol2-mediated transgenesis in zebrafish. Development *138*, 4327-4332.

S19. Balciunas, D., Wangensteen, K.J., Wilber, A., Bell, J., Geurts, A., Sivasubbu, S., Wang, X., Hackett, P.B., Largaespada, D.A., McIvor, R.S., et al. (2006). Harnessing a high cargo-capacity transposon for genetic applications in vertebrates. PLoS Genet *2*, e169.

S20. Schneider, C.A., Rasband, W.S., and Eliceiri, K.W. (2012). NIH Image to ImageJ: 25 years of image analysis. Nat Methods *9*, 671-675.

S21. Lowe, A.S., Nikolaou, N., Hunter, P.R., Thompson, I.D., and Meyer, M.P. (2013). A systems-based dissection of retinal inputs to the zebrafish tectum reveals different rules for different functional classes during development. J Neurosci *33*, 13946-13956.

S22. Peirce, J.W. (2008). Generating Stimuli for Neuroscience Using PsychoPy. Front Neuroinform *2*, 10.

S23. Chen, T.W., Wardill, T.J., Sun, Y., Pulver, S.R., Renninger, S.L., Baohan, A., Schreiter, E.R., Kerr, R.A., Orger, M.B., Jayaraman, V., et al. (2013). Ultrasensitive fluorescent proteins for imaging neuronal activity. Nature *499*, 295-300.

S24. Nikolaou, N., and Meyer, M.P. (2015). Lamination Speeds the Functional Development of Visual Circuits. Neuron *88*, 999-1013.

S25. Bulina, M.E., Chudakov, D.M., Britanova, O.V., Yanushevich, Y.G., Staroverov, D.B., Chepurnykh, T.V., Merzlyak, E.M., Shkrob, M.A., Lukyanov, S., and Lukyanov, K.A. (2006). A genetically encoded photosensitizer. Nat Biotechnol *24*, 95-99.

S26. Teh, C., Chudakov, D.M., Poon, K.L., Mamedov, I.Z., Sek, J.Y., Shidlovsky, K., Lukyanov, S., and Korzh, V. (2010). Optogenetic in vivo cell manipulation in KillerRed-expressing zebrafish transgenics. BMC Dev Biol *10*, 110.

S27. Bulina, M.E., Lukyanov, K.A., Britanova, O.V., Onichtchouk, D., Lukyanov, S., and Chudakov, D.M. (2006). Chromophore-assisted light inactivation (CALI) using the phototoxic fluorescent protein KillerRed. Nat Protoc *1*, 947-953.

S28. Nikolaev, A., Leung, K.M., Odermatt, B., and Lagnado, L. (2013). Synaptic mechanisms of adaptation and sensitization in the retina. Nat Neurosci *16*, 934-941.

S29. Hirata, H., Ogino, K., Yamada, K., Leacock, S., and Harvey, R.J. (2013). Defective escape behavior in DEAH-box RNA helicase mutants improved by restoring glycine receptor expression. J Neurosci *33*, 14638-14644.

S30. Inoue, D., and Wittbrodt, J. (2011). One for all--a highly efficient and versatile method for fluorescent immunostaining in fish embryos. PLoS One *6*, e19713.

S31. Niell, C.M., and Stryker, M.P. (2008). Highly selective receptive fields in mouse visual cortex. J Neurosci *28*, 7520-7536.

S32. Swindale, N.V. (1998). Orientation tuning curves: empirical description and estimation of parameters. Biol Cybern *78*, 45-56.

S33. Batschelet, E. (1981). Circular statistics in biology (London: Academic Press).

S34. Schindelin, J., Arganda-Carreras, I., Frise, E., Kaynig, V., Longair, M., Pietzsch, T., Preibisch, S., Rueden, C., Saalfeld, S., Schmid, B., et al. (2012). Fiji: an open-source platform for biological-image analysis. Nat Methods *9*, 676-682.
